# The Efficacy of Nutritional Interventions in Reducing Childhood/Youth Aggressive and Antisocial Behavior: A Mixed‐Methods Systematic Review and Meta‐Analysis

**DOI:** 10.1002/cl2.70059

**Published:** 2025-08-10

**Authors:** Barna Konkolÿ Thege, Chaz Robitaille, Lujayn Mahmoud, Eden A. Kinzel, Rameen Qamar, Jamie Hartmann‐Boyce, Olivia Choy

**Affiliations:** ^1^ Waypoint Centre for Mental Health Care Waypoint Research Institute Penetanguishene Ontario Canada; ^2^ Department of Psychiatry University of Toronto Toronto Ontario Canada; ^3^ Gerstein Science Information Centre University of Toronto Toronto Ontario Canada; ^4^ Department of Health Promotion and Policy University of Massachusetts Amherst Amherst Massachusetts USA; ^5^ Department of Psychology, School of Social Sciences Nanyang Technological University Singapore Singapore

## Abstract

Aggressive/antisocial behaviors in children and youth may result in impairments in family, social, or academic functioning and lead to long‐term negative consequences for both the individual and society as a whole. The potential of healthy diet and nutritional supplements to reduce aggression and antisocial behavior is an active area of study in nutritional mental health sciences. The goal of this systematic review is to (1) investigate the effectiveness/efficacy of nutritional interventions (dietary manipulation, fortification or supplementation) in reducing excessive aggression, antisocial behaviors, and criminal offending in children/youth (systematic review and meta‐analysis); and (2) provide an overview of implementation barriers and facilitators regarding nutritional interventions in children/youth (qualitative/narrative synthesis). After consulting the Campbell Collaboration's methodological guidelines, a comprehensive search for published and unpublished papers on controlled intervention studies was performed (up to February 26, 2024) using both electronic databases (MEDLINE, Embase, Cochrane Library, APA PsycInfo, Scopus, and the Allied and Complementary Medicine Database) and other resources (e.g., Google Scholar, reference list of included studies and other reviews, websites of public health agencies). This study focuses on children and youth (up to the age of 24) presenting with an above‐average level of aggression/antisocial behavior. In terms of the intervention, we considered both dietary manipulation and nutritional supplementation with a duration long enough (minimum of 1 week) that a significant change in the individual's nutritional status could be expected. We included studies with a controlled design if, for outcomes, they reported on (1) behavioral‐level violence/aggression toward others in real‐life (non‐simulated) settings, (2) antisocial behaviors, or (3) criminal offending. Initial screening, checking for eligibility criteria, data extraction from, and risk of bias assessment for each eligible study were conducted independently by two reviewers. To perform the meta‐analysis, data from each original report were standardized (transformed into Hedges' *g*) so that results across studies could be meaningfully combined and interpreted. Data conversions, computation of pooled effect sizes, and estimation of publication bias were conducted using the Comprehensive Meta‐analysis software (Version 4). Altogether, 51 reports (describing 50 individual studies) met our inclusion criteria, and 72 effect sizes were extracted from these reports. Nutritional interventions with a broad target (e.g., broad‐spectrum micronutrient supplementation or general improvement in diet quality) had the most consistent and largest intervention benefit across all outcomes (aggression: *k* = 7, *N* = 797, *g* = −0.31, 95% CI = −0.50 to −0.12, *p* = 0.001; antisocial behavior: *k* = 13, *N* = 2109, *g* = −0.49, 95% CI = −0.73 to −0.24, *p* < 0.001; criminal offending: *k* = 2, *N* = 117, *g* = −1.25, 95% CI = −2.39 to −0.11, *p* = 0.031). These intervention benefits range from small (aggression) through medium (antisocial behavior) to large (offending), and for each outcome, dietary change was considerably more effective than supplementation. Eliminating studies with high risk of bias reduced the treatment benefit to close to zero regarding aggression and small in relation to antisocial behaviors. The most commonly studied narrow‐focused nutritional intervention was omega‐3 fatty acid supplementation with a small, beneficial effect and with less consistency in statistical reliability across outcomes (aggression: *k* = 9, *N* = 706, *g* = −0.33, 95% CI = −0.87 to 0.22, *p* = 0.240; antisocial behavior: *k* = 21, *N* = 2,081, *g* = −0.15, 95% CI = −0.26 to −0.03, *p* = 0.013). Eliminating studies with a high risk of bias did not change the small effect size related to aggression, but further reduced the negligibly small effect size regarding antisocial behavior. Pooled effect size for vitamin D supplementation showed small‐to‐moderate treatment benefit in terms of antisocial behaviors (*k* = 4, *N* = 226, *g* = −0.48, 95% CI = −0.74 to −0.22, *p* < 0.001). Elimination of studies with a high risk of bias reduced the effect size from moderate to small. The overall number of and sample size in some of the studies limit our confidence in the validity of the above meta‐analytic results. Further, prediction intervals always included the possibility of no effect, suggesting considerable heterogeneity in the data. While there were several studies on other narrow‐focused nutritional interventions, these were not studied frequently enough to allow the generation of even preliminary meta‐analytic conclusions. A large set of barriers and facilitators to the implementation of nutritional interventions was also identified and narratively summarized. While the evidence on nutritional interventions to reduce aggression/antisocial behaviors is not conclusive, these interventions are safe (especially compared to psychotropic medications), easy‐to‐implement (especially nutritional supplements), and cheap (especially on the societal level). These characteristics make such interventions feasible and desirable, especially as their benefits are not aggression‐specific: good nutritional status is the basis for both physical and mental health in general; and therefore, investment in – especially broad‐spectrum – nutritional interventions seems warranted.

## 1 Plain Language Summary

### 1.1 Certain Nutritional Interventions May Be Effective in Reducing Childhood/Youth Aggressive and Antisocial Behavior as Well as Criminal Offending

### 1.2 The Review in Brief

Nutritional interventions targeting a large number of nutrients (e.g., general improvement in diet or provision of food supplements with a wide array of vitamins and minerals) may be effective in reducing aggression (small effect), antisocial behavior (moderate effect), and offending (large effect). Omega‐3 fatty acid supplementation has a small effect in reducing both aggression and antisocial behavior. Vitamin D supplementation has a moderate effect in reducing antisocial behaviors.

### 1.3 What Is This Review About?

Aggressive or antisocial behaviors in children and young people can cause problems at home, with friends, at school, as well as in other settings, and may lead to long‐term challenges for both the individual and society. Researchers are exploring whether changes in nutrition might help reduce these behaviors. A nutritional intervention refers to any action taken to improve the availability of nutrients in the body, and it can involve three main approaches: (1) making changes in what foods a person regularly eats, like eating more vitamin D‐rich foods; (2) fortifying common foods by adding extra nutrients, like drinking milk with added vitamin D; and (3) taking supplements, which are pills or other products that provide specific (group of) nutrients, like a multivitamin supplement. This review looked at whether any nutritional changes could help reduce aggression, antisocial behaviors (like theft, vandalism, or other misbehavior), or crimes in children and young adults.

### 1.4 What Is the Aim of This Review?

This Campbell systematic review examines the effects of nutritional changes on aggression, antisocial, or criminal behaviors in children and young adults (up to the age of 24). This article summarizes previously collected data from 50 individual studies completed by other researchers.

### 1.5 What Are the Main Findings of This Review?

#### 1.5.1 What Studies Are Included?

All 50 studies compared an intervention group and a control group (participants not getting the active nutritional intervention). The studies spanned the period from 1978 to 2023 and were mostly carried out in the United States, Europe, and Asia. Sixteen percent of the studies were conducted on an exclusively male sample, while 82% were conducted on mixed samples of males and females, but the average proportion of males across all studies was quite high (79%). The number of studies on offending and using vitamin D supplementation was quite low; therefore, the results regarding these are much more uncertain.

#### 1.5.2 Do Nutritional Changes Help Reduce Aggression, Antisocial Behaviors, and Offending?

Nutritional interventions targeting a large number of nutrients are effective in reducing aggression (7 studies, small effect), antisocial behavior (13 studies, moderate effect), and offending (2 studies, large effect). For all three outcomes, diet change was considerably more effective than supplementation. Omega‐3 fatty acid supplementation has a small effect in reducing both aggression (9 studies) and antisocial behavior (21 studies). Vitamin D supplementation has a moderately large effect in reducing antisocial behaviors (4 studies). Eliminating studies with lower methodological quality decreases intervention effectiveness in some cases (broad‐range nutritional interventions for aggression and antisocial behavior, omega‐3 fatty acid or vitamin D supplementation for antisocial behavior), while it does not result in considerable changes regarding others (broad‐range nutritional interventions for offending, omega‐3 fatty acid supplementation for aggression). The data also suggest that the results may not be consistent across different populations or interventions. This means that in future research or implementation efforts, nutritional interventions in certain populations will not be effective in reducing aggression, antisocial behaviors, or offending. While there are several studies on nutritional interventions other than those mentioned above, all seven of them are examined in a single study only, thus not allowing any conclusions beyond those of the original authors.

#### 1.5.3 How Has This Intervention Worked?

Some of the studies found that the increased nutrient levels in participants' blood were associated with the improvement in behavior, which provides support for the assumption that a good nutrient supply in the body supports a better functioning of the nervous system, which in turn leads to better adaptation to the social environment (less antisocial behaviors).

This review also describes a large number of factors that can support or hinder the successful use of nutritional interventions (e.g., support vs. resistance from family members regarding dietary changes, costs vs. health insurance coverage for food supplements, etc.).

### 1.6 What Do the Findings of This Review Mean?

The results of this review should not be considered complete or definitive. Instead, they should be seen as one of the preliminary attempts to characterize a new research– and clinical field (i.e., nutritional‐behavioral sciences) still in its infancy. While many further questions remain to be answered regarding the effectiveness of nutritional interventions in reducing aggression/antisocial behavior/offending, these interventions are safe, relatively easy‐to‐implement, and cheap. Given that better nutrition not only has the potential to reduce antisocial behaviors but is the basis for both physical and mental health in general, investment in nutritional interventions on all levels of society seems warranted.

More research is needed in more similar samples and using more similar nutritional approaches (e.g., several studies investigating the effects of the Mediterranean diet in young adult offenders) so clearer conclusions can be drawn on who and what type of nutritional interventions are effective in reducing aggressive and antisocial behaviors or criminal offending.

### 1.7 How Up‐to‐Date Is This Review?

The review authors searched for studies up to February 26, 2024.

## 2 Background

### 2.1 The Problem, Condition, or Issue

Aggressive behaviors are common in children and youth and may, in some cases, be developmentally appropriate. However, above‐normal levels of aggressive behavior may result in impairments in family life (e.g., deteriorated relationships with siblings) as well as social or academic functioning. Further, it may have acute safety risks and lead to long‐term negative consequences both in the internalizing (e.g., depression) and externalizing problem domains (e.g., antisocial or delinquent behavior resulting in incarceration) (Adesanya et al. 2022; Cleverley et al. 2012). Individuals with long‐term, excessive levels of aggression as children and young adults are reported to have a wide range of difficulties, including more rule‐breaking behaviors, substance abuse, marital problems, and lower educational as well as occupational attainment (Huesmann et al. 2009). Other data reveal that persistent aggressive behaviors in minors are associated with early sexual activity, early pregnancy, school dropout, and unemployment (Bradshaw et al. 2010).

When aggressive behavior becomes severe and persistent, it can also be the manifestation or correlate of a psychiatric disorder, such as antisocial personality disorder, oppositional defiant disorder, conduct disorder, attention deficit hyperactivity disorder, autism spectrum disorder, trauma‐related disorders, and others (Ford et al. 2012).

First‐line treatment recommendations for excessive aggression in children and youth include psychosocial interventions according to clinical practice guidelines (e.g., T‐MAY guidelines) (Scotto Rosato et al. 2012). These interventions include parent training (supported by the strongest evidence base and largest effect size [Chorpita et al. 2011; Pietro et al. 2018]) as well as cognitive and cognitive‐behavioral approaches (Lee and DiGiuseppe 2018), among others. Such psychosocial interventions, however, are often suboptimally accessible (e.g., due to costs or the unavailability of professionals to deliver them). Pharmacotherapy may be considered once psychosocial interventions have been shown to be inadequate or unfeasible; treatment guidelines support medications from a variety of classes to treat excessive aggression in children and youth (Gorman et al. 2015). However, the side effect burden is significant (Gracious et al. 2015), and many of these pharmacotherapeutic options are largely not recommended by some more recent guidelines (Gorman et al. 2015). As such, several limitations exist among available psychosocial and psychopharmacological treatment options for excessive aggression in terms of access, safety, efficacy, as well as patient and family preferences (Barzman and Findling 2008; Magalotti et al. 2019; Pisano and Masi 2020).

### 2.2 The Intervention

An individual‐level nutritional intervention is a set of actions designed to improve the nutritional status of the individual using one of the following three activities: (1) dietary manipulation, which aims to modify the individual's natural eating habits (e.g., consuming more food naturally rich in vitamin D); (2) fortification, which is the addition of nutrients to the basic foods the individual consumes (e.g., drinking milk with added vitamin D), and (3) supplementation, which entails administering a specific (set of) nutrient(s) separate from the components of the individual's default diet (e.g., taking a vitamin D supplement) (Martínez‐López et al. 2022).

While dietary manipulation should be the first choice when trying to improve the nutritional status of individuals, such attempts often fail due to a variety of reasons. It is well‐documented, for example, that difficulties with adherence to dietary modifications hinder the effectiveness of such interventions (World Health Organization 2003). Further, above‐average nutritional needs due to inherited metabolic characteristics, chronic stress, use of certain medications, or poor gut health as well as reduced nutrient content of natural food sources are all potential factors that can prevent dietary modifications from reaching their intended purpose (Rucklidge et al. 2021).

Nutritional supplements are widely accepted and commonly used. According to a Canadian general population survey, 46.9% of women and 33.5% of men reported taking at least one nutritional supplement in the month preceding the survey (Vatanparast et al. 2010). The same values in a study conducted in the United States were 53% and 44%, respectively (Bailey et al. 2011), while yet another study reported that 33% of children and adolescents used dietary supplements in the years before the survey (Qato et al. 2018). Supplementation is also common for the specific purpose of alleviating certain mental health disorder symptoms (e.g., 31%–33% for attention deficit hyperactivity disorder [Chan et al. 2003; Gardiner et al. 2008]).

In addition, nutritional interventions can be relevant to a criminal justice‐involved population because the prevalence of nutritional deficiency in incarcerated samples is high. For example, one recent meta‐analysis estimated the prevalence of vitamin D deficiency in individuals in prisons worldwide to be 55% (Tripathy et al. 2023). This finding may be attributed not only to challenges incarcerated individuals face in accessing healthy diets (Smoyer 2019), but also to food choices people who have offended make that undermine the nutritional balance of institutionally provided meals as well as other environmental factors such as limited sunlight exposure. This is evidenced by the finding in imprisoned young people in the United Kingdom that despite being given a diet with nutrient contents close to recommended guidelines, the intakes of some vitamins and minerals, such as vitamin D and selenium, fell below recommendations (Eves and Gesch 2003). Failure to achieve recommended nutrient intakes in criminal justice facilities has similarly been documented in other countries such as Australia and the United States (e.g., Hannan‐Jones and Capra 2016; Jacobs and Mullany 2015). These findings suggest that it would be apt to consider the impact of dietary modifications in youths in these institutional settings. Furthermore, the applicability of nutritional interventions to a criminal justice‐involved population of youths is strengthened by the fact that, unlike individuals in other institutional settings, prisoners are entirely dependent on food provided in the prison for their nutritional well‐being. Such interventions also align with overarching aims to promote health within prison contexts including in the United Kingdom (Woodall 2016).

### 2.3 How the Intervention Might Work

The role of diet, especially the negative behavioral consequences of consuming large amounts of ultraprocessed foods (Monteiro et al. 2019), has been of considerable interest in recent years (Mesirow et al. 2017; Prescott, Logan, D'Adamo, et al. 2024; Prescott, Logan, LaFata, et al. 2024). The potential of nutrients such as polyunsaturated fatty acids (PUFAs), vitamins, minerals, and amino acids to reduce aggression and violence is also an active area of study within the field of nutritional psychiatry (Rucklidge et al. 2015).

A body of literature documents links between nutritional status and a variety of antisocial behaviors. For instance, in a US study of 127 male prisoners, 35% of the variance in violent infractions and 52% of the variance in total infractions of the inmates was explained by differences in macro‐ and micro‐nutritional status (estimated using dietary records) (Bier 2001). In a cross‐sectional study of 1324 Australian adolescents, delinquent/aggressive behaviors were significantly associated with the Western dietary pattern (Oddy et al. 2009), while in a US study of adolescents, sugary soft drink consumption was positively associated with carrying a weapon and violence against peers and family members and the relationship with aggression remained significant even after controlling for numerous sociodemographic and lifestyle variables (Solnick and Hemenway 2012). A study of 670 Iranian adolescent girls indicated that dietary intakes of fiber, α‐carotene, and β‐carotene were negatively associated with self‐reported aggression (Khayyatzadeh et al. 2019).

In prospective longitudinal studies of children and young adults, there is evidence that children with malnutrition in the first years of life exhibited more conduct problems in adolescence, even after controlling for psychosocial adversity (e.g., Galler et al. 2012; Liu et al. 2004). Moreover, a higher intake of processed foods, red meat, high‐fat dairy products, and high‐sugar foods at age 11 years was associated with increased externalizing behaviors at age 14 in a large prospective study of Australian youth (Trapp et al. 2016). Another longitudinal study from a lower‐income country (Colombia) showed that a diet richer in dairy products and higher quality meat – in contrast to diets rich in carbohydrates or lower quality meat without dairy products – predicted a lower level of aggression 5–9 years later, even when controlling for numerous sociodemographic confounders (Robinson et al. 2021). A large, representative study of Brazilian students revealed a significant association between an unhealthy diet (containing more processed food and refined carbohydrates, and less fruits, vegetables, and legumes) and bullying perpetration, including sexual harassment and physical aggression, again after controlling for numerous sociodemographic confounders (Okada et al. 2024).

Another strand of evidence for the association between nutritional status and offending/antisocial behavior stems from research on food insecurity. For example, poor nutrition during pregnancy due to war‐related famine has been found to predict adult antisocial behavior in the offspring in a Dutch retrospective cohort study (Neugebauer 1999). In an assessment of delinquent behaviors that included not only aggressive acts but also non‐aggressive ones such as substance use, truancy, and vandalism, children who were raised in households with limited or uncertain abilities to acquire nutritionally adequate foods exhibited higher levels of delinquency (Jackson et al. 2018). The link between poor nutritional status and antisocial behavior may be exacerbated by the fact that food preferences and choices are rooted in factors such as socioeconomic status that are also risk factors for offending. Additional support for the nutrition‐antisocial behavior relationship comes from evidence that improvements in early childhood nutrition can lead to reductions in offending at age 24 years, with each additional year of a nutritional assistance program reducing the likelihood of a criminal conviction in young adulthood by 2.5% (Barr and Smith 2023). Notably, the significant and larger reductions in convictions following nutritional assistance were observed for violent crimes, but not for property crime (Barr and Smith 2023).

Finally, intervention studies with a single‐group pre–post design also provide additional support for the notion that aggressive/antisocial behaviors are related to nutritional status. For instance, in an open‐label study of 31 Australian male children and adolescents, parents reported medium‐to‐large decrease in violent behaviors after 16 weeks of supplementation with a broad range of micronutrients (Hambly et al. 2017). In contrast to the prior, small study, a multistate US study with the inclusion of 10 correctional institutions and over 5000 institutionalized juveniles concluded that a reduced‐sugar, higher‐fruit‐and‐vegetable diet resulted in 21%–54% (depending on institution) reduction in antisocial behaviors (Schoenthaler 1983b).

While the above‐mentioned studies do not provide definitive evidence for a causal relationship due to their observational nature, they point in the direction that certain dietary patterns may lead to an increase in the occurrence of antisocial/violent behaviors and consequently, improving nutritional status may reduce aggressive behaviors. The neurobiology of excessive aggression is complex and poorly understood. Neurochemical systems can impact aggression in at least two ways: by influencing central nervous system development during critical periods and modulating neuronal functioning of the already developed nervous system throughout life (Rosell and Siever 2015). Both the serotonin and dopamine systems have been shown to play a role in modulating aggression in addition to GABA, oxytocin, testosterone, and cortisol (Siever 2008).

Adaptive aggression regulation is dependent on both the healthy development of the nervous system in childhood and adolescence as well as an ongoing balance between bottom‐up subcortical processes and top‐down cortical modulation (Siever 2008). All of these processes are reliant on – among other factors – the adequate level of nutrients available in the body to support the development and optimal functioning of the central nervous system (Roberts et al. 2022). For instance, omega‐3 fatty acids are necessary for general neurodevelopment as they are building blocks of brain cell membranes including neuronal synapses (Gajos and Beaver 2016).

**Figure 1:**
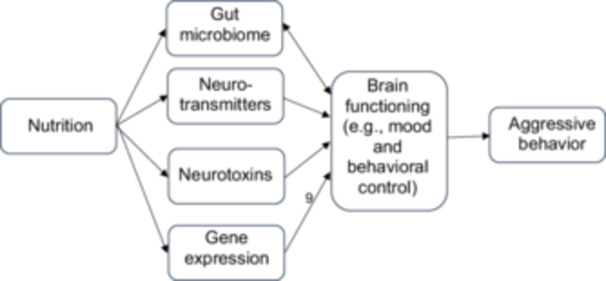
Proposed mechanisms underlying the association between nutritional status and aggressive/antisocial behavior.

In addition to playing a more direct role in brain cell growth and development, there are several proposed mechanisms by which nutrients may affect brain function which, in turn, influences behavior (Figure 1). First, micronutrients (vitamins and minerals) serve as co‐factors in the synthesis of neurotransmitters (e.g., serotonin, dopamine, GABA) (Rucklidge et al. 2021). Other nutritional factors such as omega‐3 fatty acid deficiency can also lead to dysfunctional serotonin synthesis, activation, and function (Patrick and Ames 2015), while a high protein intake can impact the metabolism of tyrosine which is a precursor of dopamine (Muth and Park 2021), highlighting the effect of nutrition on changes in neurochemistry. Second, as nutrition is an important modulator of toxicity from environmental chemicals, malnutrition can affect the brain by exacerbating neurotoxins, while intake of nutrients associated with neuroprotective effects can help to retain brain structural integrity (Muth and Park 2021). Third, micronutrients are also involved in the methylation‐folate cycle which can affect genetic expression (Rucklidge et al. 2021). A fourth mechanism involves the gut microbiome, which is also influenced by diet. Signals sent along the gut‐brain axis make their way to the brain, contributing to the regulation of behavior (Dinan et al. 2015; Tcherni‐Buzzeo 2023). Although direct empirical evidence on the link between the gut‐brain axis and human aggression is currently lacking, this pathway from microbiome to aggression has been proposed based on indirect evidence regarding the association of the microbiome with mental health outcomes and psychological factors related to aggressive behavior. For instance, some significant differences in the gut microbiota were found between children and adolescents with attention‐deficit/hyperactivity disorder and control groups (Soltysova et al. 2022), while gut microbial structure was found to be associated with temperament in young children (Christian et al. 2015). Consequently, adaptive aggression regulation is also dependent on nutritional status, which if suboptimal, can be improved by nutritional interventions.

### 2.4 Why It Is Important to Do This Review

Among the psychosocial interventions that have the potential to decrease aggression in children and youth, many are relatively difficult to access (e.g., require highly trained professionals for whom the demand highly exceeds supply) or punitively expensive either to service users directly or society as a whole. In contrast, nutritional interventions cost less (e.g., nutritional supplements vs. psychiatric medications [Kaplan et al. 2017]) or nothing (e.g., elimination diets) and so are generally more accessible. If the present synthesis of the evidence can confirm the effectiveness of (certain) nutritional interventions in reducing aggression/violence in children and youth, stakeholders and policy makers will have tools, which are readily employable potentially even on larger scales given current Western societies' high level of interest in healthy nutrition.

Finally, many dietary interventions investigated to reduce aggression are not just helpful in reducing aggression in an isolated manner; instead, they often have a broader beneficial effect on mental health (Kaplan and Rucklidge 2021; Zhang et al. 2024), given the importance of healthier nutrition in relation to (brain) health in general.

The present review is needed as prior reviews on this topic are vastly outdated (Benton 2007), specific to a single nutritional intervention (Gajos and Beaver 2016; Hibbeln and Gow 2014; Raine and Brodrick 2024) or a subgroup of them (Rucklidge and Kaplan 2013), do not contain quantitative synthesis of the data (Qamar et al. 2023; Qureshi et al. 2021) or focus on supplementation but not dietary modifications. They also do not cover a broader conceptualization of problem behaviors (cf. additional focus on antisocial behaviors and offending in the present review) but largely focus on other‐directed aggression only. Finally, prior reviews do not explore implementation challenges and potential solutions to these issues.

## 3 Objectives

The goal of this systematic review is to answer the following questions based on the available empirical evidence:

1. Are there nutritional interventions (dietary manipulation, fortification or supplementation) that can reduce excessive aggression towards others in children/youth? If yes, how strong is their effect and is there a difference among the intervention types?
2. Are there nutritional interventions that can reduce antisocial behaviors in children/youth? If yes, how strong is their effect and is there a difference among the intervention types?
3. Are there nutritional interventions that can reduce criminal offending in children/youth? If yes, how strong is their effect and is there a difference among the intervention types?
4. What implementation barriers and solutions to these exist in relation to the above nutritional interventions in children/youth?

## 4 Methods

This review followed the methodological expectations of intervention reviews published by the Campbell Collaboration (Methods Coordinating Group of the Campbell Collaboration 2019). We also closely followed the protocol developed for this systematic review (Konkolÿ Thege et al. 2024); in the small number of cases where we deviated from this protocol, we comment on our rationale explicitly in the methodological section below.

### 4.1 Criteria for Considering Studies for This Review

#### 4.1.1 Types of Studies

Considering the relatively novel nature of the field of nutritional psychiatry (Adan et al. 2019; Dinan 2023; Marx et al. 2017; Sarris et al. 2015) and accordingly, the limited amount of data accumulated to date, we considered any prospective study employing a controlled design (having an intervention and a comparison group) for the purposes of the quantitative evidence synthesis. Consequently, we did not only consider randomized (or quasi‐randomized) controlled trials but non‐randomized (but controlled) studies as well.

We did not consider epidemiological studies cross‐sectionally describing the association between nutritional status/dietary patterns and the selected outcome variables as this review focuses on the efficacy of *interventions*. We did not consider uncontrolled studies with a simple pretest–posttest design either due to concerns of internal validity.

All types of studies (theoretical, quantitative with any designs, qualitative) were considered in the qualitative/narrative synthesis of the present review aiming to support the better understanding of the barriers and facilitators of the successful implementation of nutritional interventions.

#### 4.1.2 Types of Participants

This study focuses on children and youth presenting with elevated levels of aggression. From the age perspective, relevant study participants were defined as individuals up to the age of 24 (regardless of sex/gender). This particular age as the exit from youth is somewhat arbitrary and debatable; however, individuals aged 18–24 are often considered as transitional‐aged youth with characteristics and needs somewhat different from those of minors or adults. Further, in the context of nutrition and brain functioning, the early twenties is the age when maturation of the brain becomes mostly complete (Arain et al. 2013); in contrast to the age of 18, which could be used as an alternative threshold for adulthood, but more from the legal than the neurodevelopmental or psychological perspective. When a study included participants whose age was more than 24 years and there was no way to separate those at or under this age, the study was included in the review if the mean age was 24 years or less (i.e., Gast et al. 2023; Zaalberg et al. 2010). When the mean age of the sample was more than 24 years and we could not separate out data from those 24 and younger, the study was excluded even if it contained some participants who would have been relevant for this review (see list of such studies with the exclusion reason of “Non‐eligible age group” in the Supporting Information S1: “Excluded studies”).

In terms of excessive aggression, it is important to note that a certain level of aggression, especially with younger children, can be age‐appropriate. This review considers data in relation to *elevated*/*maladaptive* levels of aggression, for which we sought at least one indicator. These indicators were (1) mental health care utilization for/diagnosis of/reliably assessed and above‐threshold level of symptomatology of mental disorders often co‐occurring with aggression such as attention‐deficit/hyperactivity disorder, conduct disorder or autism; (2) being in the criminal justice system for any reason either in a prison, probational, or special educational setting; or (3) being characterized by above‐normal, self‐rated or observer‐rated scale scores measuring aggression/antisocial traits. Studies on participants with no – even indirect as described above – indication of excessive level of aggression (either at baseline or intervention end) were excluded as no effect is anticipated from a nutritional intervention in relation to normative level of aggression (in most such studies, aggression‐related variables were secondary outcomes only and therefore, excessive level of aggression did not play a role in participant selection). The list of studies on samples with a normal level of aggression can be found in the Supporting Information S1: “Excluded studies” with the designation “No excess aggression in sample.”

#### 4.1.3 Types of Interventions

We considered two main types of nutritional interventions; namely, dietary manipulation and nutritional supplementation (including fortification or the use of supplements), both of which should be long enough in duration (minimum of 1 week) so that a significant change in the individual's nutritional status could be expected.

Dietary manipulation is an attempt to intentionally change an individual's natural food consumption patterns to improve nutritional status. The aim of such interventions is to either (1) increase the consumption of certain foods rich in nutrients which are not readily available in the individual's system in the required amount; or (2) decrease the consumption of or completely eliminate certain foods containing substances which are (a) necessary and helpful up to a certain amount but harmful in excess (e.g., carbohydrates) or; (b) unnecessary or directly harmful for the human body (e.g., certain food additives that get into food through industrial food production).

In contrast, nutritional supplementation/fortification exclusively aims to increase the availability of nutrients in the individual's system. In the case of nutritional supplements, this happens through the consumption of manufactured products (in the form of pills, capsules, tablets, or liquids) that are regulated as dietary supplements (and not medications, thus not requiring prescription by a physician). Both nutritional supplementation and fortification intend to supplement the diet with substances that have been confirmed as being essential to life, which can be micronutrients such as vitamins (vitamin A, vitamin B, etc.) and minerals (calcium, magnesium, zinc, etc.), macronutrients (e.g., fatty or amino acids) or a combination of these. Phytoceuticals, that is, plant‐based natural products were also considered but not found. Supplementation was defined as the consumption of manufactured supplements in addition to diet.

A list of identified studies with nutritional interventions not satisfying the above criteria (e.g., one‐time, high‐sugar drink intake in a laboratory environment to test the negative effects of excess sugar) can be found in the Supporting Information S1: “Excluded studies” with the designation “Non‐eligible intervention.”

The comparators in the included studies were quite diverse, ranging from placebo (e.g., tablets/capsules with identical outer characteristics as the supplement) through simple healthy food advice to waitlist control or “treatment as usual.” While we were planning to consider both active and passive comparators (e.g., placebo, treatment as usual), we found a single eligible study with an active comparator (even there, the comparator, relatively large amount of olive oil, had been conceptualized by the study authors as passive but proved to be active both in light of the results of the given study and the evidence accumulated since then [Stevens et al. 2003]).

#### 4.1.4 Types of Outcomes

The primary outcomes in this review were (1) behavioral‐level violence/aggression toward others (people or objects) in real‐life (non‐simulated) settings; (2) antisocial behaviors; and (3) criminal offending.

Violent/hetero‐aggressive behavior was defined as intentionally causing or attempting to cause emotional or physical harm or damage to somebody other than the acting person. We considered both reactive aggression, that is, impulsive violence or threat‐driven aggression, as well as proactive aggression or violence committed with the purpose of increasing one's dominance or to obtain property (Fossati et al. 2009). In accordance with the above considerations, studies were excluded if they investigated: (1) aggressive/angry/hostile emotions or thoughts without observable behaviors; or (2) aggressive tendencies presented in simulated environments (e.g., level of aggression expressed in a video game play situation, which is thought to be qualitatively different from real‐life situations). The list of identified studies with such outcomes can be found in the Supporting Information S1: “Excluded studies” with the designation “No eligible outcome.”

As behavioral‐level violence and aggression in children and youth is often not studied in isolation but in addition to or as part of an amalgamation of antisocial behaviors, this more heterogeneous conceptualization – including, for example, disobedience, theft, lying, and intentional property damage – was also considered.

As a more severe form of hetero‐aggressive or – more broadly – antisocial behavior, criminal offending was considered as a separate outcome due to the high societal importance of its reduction. While we originally planned to differentiate between violent and non‐violent offending, the sparsity of the data (one study on violent offending, one on non‐violent offending, and one on non‐specified offending) did not allow us to meaningfully do so; therefore, we collapsed all criminal offending outcomes. A violent offense was defined as actual, attempted, or threatened harm directed toward another person (including non‐consensual sexual contact between the perpetrator and another person) sanctioned by the law of the jurisdiction of the perpetrator.

#### 4.1.5 Outcome Measures

Hetero‐aggression is typically operationalized by observer‐rated scales, but occasionally, self‐reported questionnaires are also used. An example of the former is the Aggression Subscale of the Child Behavior Checklist (Achenbach and Ruffle 2000), while examples of the latter include the Aggressive Behavior Scale of the Youth Self Report (Ebesutani et al. 2011), the Buss‐Perry Aggression Questionnaire (Buss and Perry 1992), and the Reactive‐Proactive Aggression Questionnaire (Raine et al. 2006). Data produced by either of these methods were considered.

Similar to aggressive behaviors, antisocial behaviors are typically operationalized by observer‐reported scales or institutional records, but occasionally other quantitative approaches (e.g., self‐reported scales) are also used. Examples of the former include the Conduct Subscale of the Strengths and Difficulties Questionnaire (Goodman 2001), the Rule‐breaking Subscale of the Child Behavior Checklist (Achenbach and Ruffle 2000), or records of institutional misconduct, while an example of the latter is the Rule‐breaking Behavior Subscale of the Youth Self‐Report (Ebesutani et al. 2011).

Offending is most often operationalized by some ratio of offending or recidivism as documented by criminal/legal records. Occasionally, self‐reports on the same are also used; such outcomes would have been considered but not found in this review.

Sometimes outcome variables are labeled differently in studies than what they actually measure, and including such studies in meta‐analyses introduces bias. Therefore, we did not automatically accept the original authors' terminology. Instead, we made significant efforts to verify that the reported data indeed related to the above outcomes of interest. For instance, a study using the “Aggression Subscale” of the Leiden Index of Depression Sensitivity–Revised was not included into our review as in contrast to its name, this subscale assesses impulsivity or angry feelings, rather than aggression (this study was included in a recent meta‐analysis on omega‐3 fatty acids and aggressive *behavior* [Raine and Brodrick 2024]).

#### 4.1.6 Duration of Follow‐Up

If multiple data collection sessions occurred during the active intervention period, end‐of‐intervention data were considered only, as we assume that the fullest intervention effect can be observed at that point (especially given the typically short intervention duration [median of 3 months] used in the extant literature; cf. Qamar et al. 2023). According to the hypothesized mechanisms of action of the intervention (cf. Section 2.3), the consistent availability of nutrients is needed on a long‐term basis for the healthy development and functioning of the central nervous system. Therefore, we did not anticipate that improved behavioral outcomes could be observed after the discontinuation of the intervention. To investigate this latter hypothesis, we also extracted follow‐up data from the first time point closest to 3‐month post‐intervention from the small number of studies where such data were available. We contrasted the intervention‐end and follow‐up data to better understand whether it is indeed unwarranted to expect effects after the discontinuation of a nutritional intervention.

#### 4.1.7 Types of Settings

While research on aggression and violence is common in clinical populations (i.e., among children and youth with a mental health disorder diagnosed by a mental health care professional), both aggression/violence and antisocial behaviors in general are commonly occurring and frequently studied phenomena in non‐clinical populations as well (e.g., in educational or criminal justice settings). We argue that the non‐clinical setting or the non‐existence of a psychiatric diagnosis is not necessarily an indication of the lack of a relevant disorder (e.g., conduct disorder, antisocial personality disorder, ADHD), but rather the lack of resources to identify and treat those mental disorders – regardless of the setting. Therefore, we did not restrict our interest to any particular setting or diagnostic category but instead, collected and synthesized the evidence on children and youth in general who present with observable, maladaptive levels of aggression or antisocial behaviors.

### 4.2 Search Methods for Identification of Studies

After consulting the Campbell Collaboration's search‐specific methodological guidelines (Kugley et al. 2017), a comprehensive search for published and unpublished studies and reports was performed to reduce the risk of publication bias and identify the best available evidence. No date or language (regarding the full text) restrictions were applied when attempting to identify relevant studies; however, the search terms were used in English only; therefore, only studies with an English‐language title and/or abstract were considered as potentially eligible.

#### 4.2.1 Electronic Searches

Comprehensive database searches were designed in collaboration with a health sciences librarian (E. A. K.), and the initial Ovid MEDLINE search was peer‐reviewed by another health sciences librarian following the PRESS protocol (McGowan et al. 2016). The search was modified slightly for each database but generally consisted of a combination of keyword terms and controlled vocabulary (when available) for each of the following concepts: (1) aggression, violence, antisocial behaviors, and offending; (2) diet, nutrition, vitamins, minerals, nutritional supplements, and dietary interventions; and (3) youth under the age of 25. The nutritional supplements concept was adapted from the Foods, Herbs, and Supplements listing from NatMed Pro, and the youth concept was adapted from the search for another review (NatMed Pro; Romano et al. 2023). Based on trial searches, several terms were added or modified compared to the published review protocol to increase the comprehensiveness of the search. The primary Ovid MEDLINE search string was run in Ovid MEDLINE (Epub ahead of print, in‐process, and other non‐indexed citations – 1946 to present) and then translated and also run in Embase (1947 to present), APA PsycINFO (1806 to present), and Allied and Complementary Medicine (1985 to present) all via Ovid; Ovid Embase, Cochrane via Wiley and Ovid APA PsycInfo, Scopus and the Allied and Complementary Medicine Database. These database searches were executed on February 26, 2024. The exact and final search strings for each of these databases are provided in the Supporting Information S2: “Electronic database search strings.”

#### 4.2.2 Searching Other Resources

The reference lists of all relevant systematic or narrative reviews identified through the database searches were checked for additional relevant primary studies. The reference list of each eligible original study was also checked to identify any additional studies missed by the database search. In addition, the corresponding author of each eligible study and further experts were contacted to (1) identify unpublished or otherwise missed but relevant primary studies and (2) seek out direct information (and sources of such information) on implementation barriers and potential solutions regarding the nutritional interventions studied.

The clinical trials register of the (US) National Library of Medicine (https://clinicaltrials.gov) and the International Clinical Trials Registry Platform (https://www.who.int/clinical-trials-registry-platform), as well as preprint databases (Research Square: https://www.researchsquare.com; medrXiv: medrxiv.org; Open Science Foundation: https://osf.io/preprints/) were searched for unpublished studies. ProQuest Dissertations & Theses and EBSCO Open Dissertations were searched for potentially relevant dissertation and thesis work. Google Scholar was also used (screening continued for the first 50 hits after the last relevant record) to make the searches as comprehensive as possible. Searches in these databases were conducted using a simplified algorithm employing different combinations of the most central search terms (i.e., nutrition, diet, aggression, violence, antisocial behavior, and offending). Additionally, we searched for relevant information on the websites of (federal‐level) public health agencies of Australia, Canada, the United Kingdom, and the United States, the e‐Library of Evidence for Nutrition Actions, the USDA Nutrition Evidence Systematic Review, and the What Works Network. Finally, the journal Evidence‐based Complementary and Alternative Medicine was hand‐searched to comply with the suggestion of an anonymous reviewer of the protocol. These searches were also completed by February 26, 2024.

### 4.3 Data Collection and Analysis

#### 4.3.1 Description of Methods Used in Primary Research

The vast majority of the eligible studies employed a randomized controlled design; however, some studies used an observational cohort design. In this latter case, we investigated – as part of the risk of bias assessment process – whether the study groups were matched on the most important participant characteristics (i.e., mental disorder diagnosis, criminal justice system involvement, age, sex, pre‐intervention diet/nutritional deficiency, psychiatric medication use, and psychosocial interventions parallel to the nutritional intervention).

#### 4.3.2 Selection of Studies

After completing the searches, all identified citations were uploaded into the EndNote 21 reference management system (https://endnote.com). Records then were transferred to the Covidence systematic review platform (https://www.covidence.org), which conducted the removal of duplicates. Titles and abstracts were screened by two independent evaluators (C.R. and L.M.), both of whom had prior experience with completing initial screening for a systematic review. The full text of all records deemed potentially relevant by either reviewer was retrieved and checked for inclusion and exclusion criteria by both reviewers. Discrepancies between reviewers in terms of eligibility assessments were resolved by consulting a third reviewer, the lead author of the study (B.K.T.).

#### 4.3.3 Data Extraction and Management

Data were extracted for the following variables: bibliographic information, paper peer‐reviewed or not, study design, general sample characteristics, study setting, whether any interventions were received by participants beyond the intervention in the study's focus (e.g., medication), pre‐intervention nutritional deficiency/diet in the sample, sample size, proportion of males, mean age as well as ethnicity and/or race of participants, details of comparator intervention (e.g., content of placebo), details of intervention (e.g., nutrient content of the supplement with dosages), intervention duration, implementation‐related information, side effects of intervention, type and assessment of outcome (if multiple, rationale for the choice for inclusion in the quantitative analyses), list of alternative (non‐selected) outcome indicators, outcome rater, drop‐out rate for nutritional intervention (to assess compliance) and the study in general (to characterize study quality), risk of bias, summary of findings according to study authors, whether results were presented in a sex/race‐stratified way, and the quantitative data to compute effect sizes in the meta‐analysis. All extracted data for each of the included studies are available in the Supporting Information S1 for this article.

Data extraction for each study satisfying eligibility criteria was conducted independently by two reviewers (C.R. and L.M.). Disagreements were resolved through consensus involving the lead author of the review (B.K.T.), who also validated all coding by cross‐checking the extracted data with the original research report. While we planned to conduct the data extraction in Covidence, we ultimately decided to complete this process using Excel, given our need to conduct outcome‐level (instead of study‐level) risk of bias assessments, which is a requirement when using the 2nd version of the Cochrane Risk of Bias Tool (see below).

#### 4.3.4 Assessment of Risk of Bias in Included Studies

The risk of bias for each study was assessed by two independent reviewers (C.R. and L.M.). Disagreements were resolved through consensus involving the lead author of the review (B.K.T.), who also validated all ratings by cross‐checking the extracted data with the original research report. As part of this process, studies were scored across several domains, resulting in an overall rating of high, moderate, or low risk of bias using the appropriate (i.e., parallel‐ vs. cross‐over) version of the revised Cochrane Risk of Bias tool for randomized studies (RoB‐2; Eldridge et al. 2021; Higgins et al. 2021; Higgins et al. 2019) and the Cochrane Risk of Bias in Non‐randomized Studies of Interventions (ROBINS‐I; Sterne et al. 2016).

#### 4.3.5 Measures of Treatment Effect

To perform the meta‐analyses, data from each original report were standardized (Hedges' *g* was computed) so that results across studies could be meaningfully combined. Hedges' *g* is preferred over Cohen's *d* for small samples, which was the case for most studies included in this review. The default and most often used source of information was absolute means and standard deviations at both baseline and intervention‐end but in situations where these were not provided, alternative data were extracted (e.g., change scores and their standard deviation, *F*‐value, mean and standard deviation at intervention‐end only, number of violent events as event counts etc.). Such alternative data were converted to Hedges' *g* by Comprehensive Meta‐analysis (version 4.0).

#### 4.3.6 Unit of Analysis Issues

If a study arm combined a nutritional and another type of intervention (e.g., psychotherapy plus nutritional supplementation), this arm was compared to the study arm where the non‐nutritional intervention was studied separately (psychotherapy only) (e.g., Raine et al. 2016). If a study included more than two study arms, no subgroup was considered twice in the same meta‐analysis (including the control group).

Originally, we planned to combine the results of cross‐over and parallel‐group randomized controlled trials only if outcome data were available from the first phase of the cross‐over study separately, that is, up to the point of cross‐over (e.g., Gast et al. 2023; Richardson and Montgomery 2005). In the absence of such data, we planned to analyze cross‐over studies in separate analyses from the parallel‐group randomized controlled trials. However, we found only a very small number of cross‐over studies per outcome with combined phase one and phase two data (one for omega fatty acid supplementation for aggression and three for omega fatty acid supplementation for antisocial behavior), which would have made such separate analyses unfeasible. Therefore, the small number of cross‐over studies were analyzed together with the rest of the studies. We did conduct, however, subgroup analyses to investigate the effect of study design on the results.

#### 4.3.7 Criteria for Determination of Independent Findings

When several indicators were reported for the same individuals and the same outcome of interest (i.e., aggression towards others, antisocial behaviors, or criminal offending), a single effect size indicator was selected for the purposes of the meta‐analysis using the following decision rules (López‐López et al. 2018):

1. Observer‐reported outcomes were prioritized over self‐reported outcomes due to the risk of social desirability effect with self‐reported data.
2. When data on reactive and proactive aggression were provided separately, an aggregated effect size based on both indicators was calculated given the equal relevance of both constructs for the purposes of this review. However, this was the case in one eligible study only (Raine et al. 2019); therefore, in contrast to our original plans laid out in the protocol (Konkolÿ Thege et al. 2024), we were not able to test in sensitivity analyses whether type of aggression (reactive or proactive) moderated treatment effect.
3. More comprehensive conceptualizations or measurements of aggression were prioritized over subscores (e.g., a total scale score was prioritized over a subscale score if all subscales measured different aspects of the same outcome).
4. If only subscale scores were reported, more severe forms of aggression were prioritized over less severe forms of aggression (e.g., physical violence over verbal threats).
5. Indicators based on assessment tools used more often in the included studies were prioritized over ad hoc or only rarely used measures to increase comparability.
6. If multiple interventions were examined within the same study while measuring the same outcome, and the only difference between the intervention variants was in dosage, we prioritized the intervention with the dosage that is closer to the current Recommended Dietary Allowances of the given nutrient(s) according to the U.S. Food and Drug Administration (Food and Drug Administration 2016). This situation was presented in two studies (Arnold et al. 2011; Schoenthaler et al. 2023); in both cases, the study arm with the lower dosage of the supplement was considered. If even after considering these principles, no clear priority could be established, the outcome to include in the given meta‐analysis was selected randomly from those outcomes satisfying the above criteria. This occurred in a single 3‐phase cross‐over study (Milte et al. 2013), where both active phases used a fatty acid supplement of similar dosages, in which case the phase with eicosapentaenoic acid supplementation was randomly selected for inclusion.

Information on all non‐selected but relevant outcomes, study arms (e.g., different dosages of the same nutritional supplement), or study periods (in case of cross‐over studies) from each eligible study were extracted to facilitate later, more nuanced analyses using correlated‐hierarchical effect models appropriately studying dependent data (which was not part of this study due to resource limitations). This information can be found in the Supporting Information S1 along with the justification for our choice regarding inclusion in the quantitative analyses.

#### 4.3.8 Dealing With Missing Data

If the required data (e.g., absolute means and standard deviations) were not available in the published original reports, we used alternative raw data available within the included studies (e.g., change scores) to generate an effect size metric. If no data were available in the original reports that would have allowed the calculation of the pooled effect size, the authors of the original reports were contacted, and the data were requested.

#### 4.3.9 Assessment of Heterogeneity

Heterogeneity amongst original studies was characterized using the *Q* and *I* ^2^ statistics (Higgins 2003). We also calculated and reported 95% prediction intervals to quantify heterogeneity wherever the number of studies allowed us to do so.

#### 4.3.10 Assessment of Reporting Biases

We assessed small‐study effect (publication bias) if there were 10 or more studies included in a given meta‐analysis. We visually analyzed funnel plots (Sterne et al. 2011) and formally examined funnel plot asymmetry using Egger's test. The trim and fill method was also used when applicable.

#### 4.3.11 Data Synthesis

First, studies were grouped according to outcomes, that is, (1) aggression toward other people or property, (2) antisocial behavior, and (3) criminal offending. Studies were then grouped according to the nutritional target of the intervention: (1) broad nutrient target (e.g., broad‐range micronutrient supplement, complete diet change), (2) omega‐3 PUFAs, (3) vitamin D, and (4) other (all single‐nutrient supplementation).

As our analyses combined data based on different scales or measures, we ensured that higher scores or odds indicated higher level of aggression/antisocial behavior. We used Comprehensive Meta‐analysis Version 4 for the statistical analyses. The random effect model was used and beyond the point estimates, 95% confidence intervals were calculated. The random‐effects model was employed (in contrast to the fixed‐effect model) as our intention was to generalize the results to populations comparable to those in the studies included in the analyses. A graphical representation of the results (forest plot) was also provided for each outcome separately.

#### 4.3.12 Subgroup Analysis and Investigation of Heterogeneity

Analyses were conducted separately for each outcome variable (hetero‐aggression, antisocial behavior, and criminal offending) and stratified by the above‐described nutritional intervention categories. Additional subgroups were also formed (if enough data were available) based on:

1. Study design (parallel‐group randomized controlled, cross‐over, or observational cohort design);
2. Study population (diagnosed with a disorder not primarily defined by aggression/antisocial behavior, such as autism or ADHD, diagnosed with a disorder primarily defined by aggression/antisocial behavior, such as conduct disorder or oppositional defiant disorder, or criminal offending);
3. Proportion of males in the sample (less vs. equal to/more than the mean proportion of males across studies, i.e., 80%);
4. Age group (mean age of participants less than 12 years old, 12–17 years old, 18–24 years old);
5. Intervention duration (less or more than 15 weeks, which was the mean intervention duration in a review with a similar focus [Qamar et al. 2023]); and
6. Intervention type (diet change vs. nutritional supplementation/fortification) in the case of interventions with a broad nutrient target, which was the only intervention group where diet change and nutritional supplementation/fortification were collapsed.

#### 4.3.13 Sensitivity Analysis

The generalizability and robustness of the results were examined by sensitivity analyses conducted after removing studies judged to be at high risk of bias.

#### 4.3.14 Treatment of Qualitative Research and Mixed‐Method Analysis

Considering the need for practical usability of the findings, beyond the standard quantitative data collection and synthesis on intervention efficacy, we also collected and synthesized data on implementation barriers and facilitators. For this purpose, we considered non‐empirical (e.g., expert opinion) as well as quantitative and qualitative empirical data. We organized these data considering the different stages of implementation and sought for and organized implementation‐related information corresponding to these stages. They include: (1) awareness of the relevance of and interest in the intervention among stakeholders, (2) access to nutritional interventions, (3) characteristics of nutritional interventions, (4) compliance with nutritional interventions, and (5) treatment‐interfering behaviors or physiological processes.

Data on these domains were extracted from all studies eligible for the quantitative analyses. We also approached the authors of eligible studies to inquire about implementation barriers and potential solutions. In addition, we searched information on implementation barriers and facilitators on websites of relevant governmental and international agencies, professional organizations, and in regular scientific articles through Google Scholar. Regarding this search process, we used the methodology of traditional narrative reviews.

## 5 Results

### 5.1 Description of Studies Included in the Quantitative Synthesis

#### 5.1.1 Results of the Search

**Figure 2:**
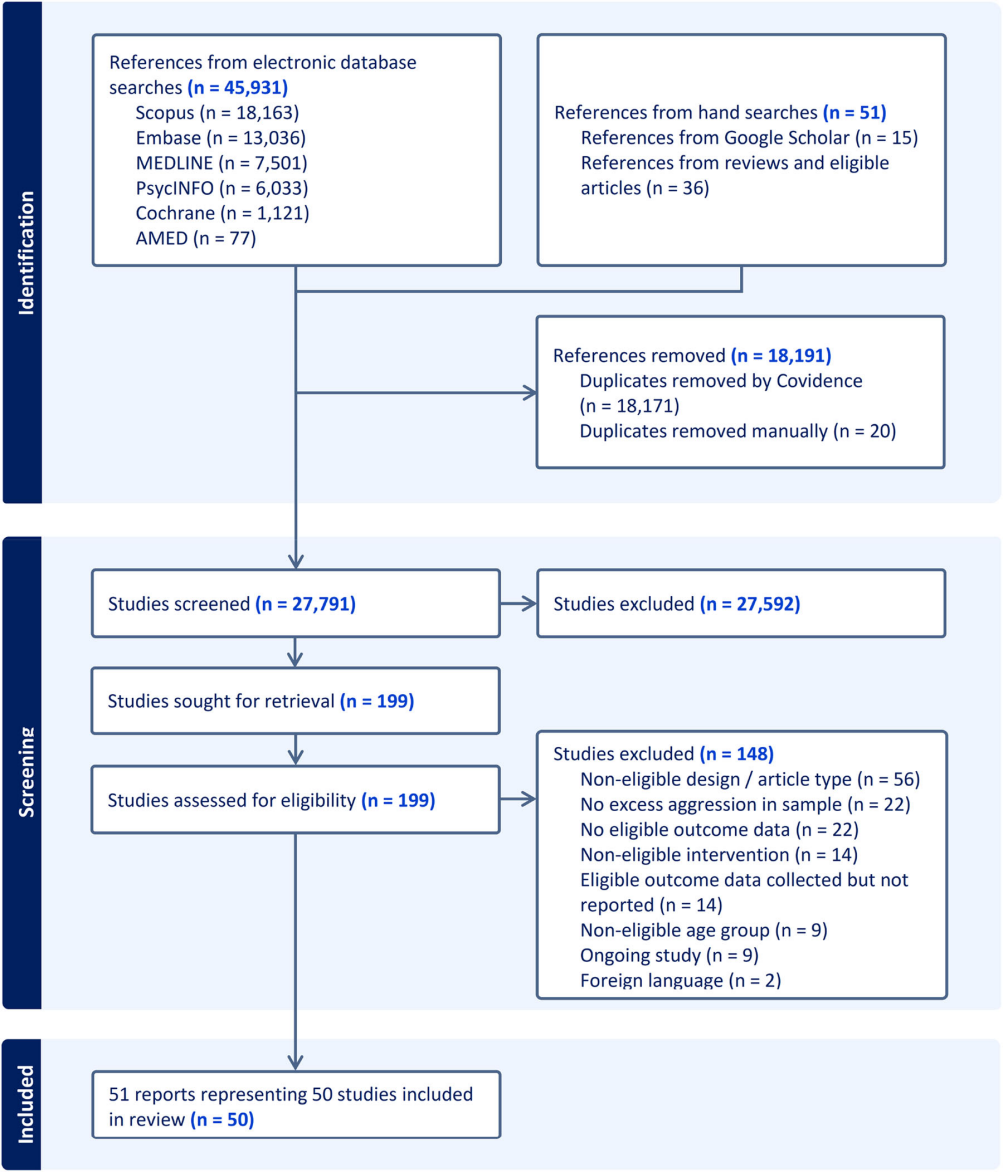
PRISMA chart of the search process.

Details of the search process are described in Figure 2. The database searches resulted in 45,931 records, while 51 additional records were identified by Google Scholar and hand‐searching the reference lists of reviews and eligible articles. Altogether, 18,191 duplicates were removed by Covidence, resulting in 27,791 records to screen. This process led to the elimination of 27,592 records, due to their irrelevance judged based on their title and abstract. After checking eligibility of the remaining 199 records, 148 studies were excluded and 51 were included.

#### 5.1.2 Included Studies

Altogether, 51 papers describing 50 studies met our inclusion criteria (the two articles of Hemamy and colleagues described two different indicators for the same outcome from the same sample [Hemamy et al. 2020, 2021]). Bibliographic details of each included study can be found in the Supporting Information S1. Of the 51 articles, 72 effect sizes were extracted/calculated due to some studies providing more than one outcome, more than two study groups for the same outcome, or more than one relevant time point. All effect size data/subgroup‐defining variable entered into the meta‐analyses can be found in the Supporting Information S1 (Excel sheet entitled “Data used in meta‐analysis”).

Of the 50 included studies, 38 (76%) used a parallel‐group randomized‐controlled design, 6 (12%) were observational cohort studies, and 4 (8%) were cross‐over randomized‐controlled trials. Although two other studies (4%) were originally designed as cross‐over, we only used data from their pre‐cross‐over phase.^1^ The first and last group were collapsed in the analyses (and for risk of bias assessment) due to no differences existing between them from a practical perspective.

Altogether, 28 (56%) studies were conducted in a non‐specified community or school setting, 8 each (16%) in an outpatient psychiatric and correctional setting, and 2 (4%) in an inpatient/residential setting, while authors of 4 (8%) studies did not provide information on the setting of their study. In terms of the geographical location of the studied samples, 18 (36%) studies were conducted in North America, 15 (30%) in Europe, 6 (12%) in the Middle East, 6 (12%) in East Asia, and 5 (10%) in Australia or New Zealand. Of all included studies, 35 (70%) included participants with a medical diagnosis not primarily defined by aggression/antisocial behavior (e.g., ADHD, autism), 10 (20%) included prisoners/detainees/probationers, while 5 (10%) included participants with a medical diagnosis primarily defined by aggression/antisocial behavior (e.g., conduct disorder) or a non‐diagnostic but direct indication of elevated level of aggression/antisocial behavior.

Of the 50 studies, 8 (16%) were conducted on an exclusively male sample, while 41 (82%) were conducted on mixed samples of males and females. In the remaining one study, the sex composition of participants was not reported (Johnson et al. 2010). The average proportion of males across all studies was high (mean = 79.0%, SD = 14.3). Averaging the mean age of participants reported in the studies resulted in a value of 10.4 years (SD = 4.1) with a range of 3.3–22.8 years. The majority of studies (*n* = 34, 68%) did not specify the race/ethnicity of their participants. In the studies where authors did so, the proportion of Caucasian participants was 63.4% on average (SD = 33.8), ranging from zero to 100%. The vast majority of the studies did not provide sex or race/ethnicity‐specific analyses. Only a single study reported on formally race‐stratified results (Schoenthaler 1983a), indicating no difference in intervention effectiveness between the White versus Black participants, while another study investigated the moderator effect of sex, again indicating no difference in intervention efficacy between boys and girls (Johnstone et al. 2022).

Out of the 50 studies, 12 (24%) investigated dietary manipulation and 38 (76%) examined the effects of supplementation. Among the studies on diet change, 10 intended to improve nutritional status considering a large number of nutrients, 1 focused on reducing sugar content, and 1 aimed primarily to increase the amino acid content (histidine) of diet. In terms of supplementation, 22 studies investigated PUFA supplements (typically omega‐3 fatty acids), 9 studied broad‐range supplements (e.g., a large number of vitamins and minerals combined), 4 investigated vitamin D, 1 studied zinc, 1 studied an amino acid (l‐tryptophan), and 1 studied magnesium supplementation.

Intervention duration was 100.8 days on average (SD = 63.5, range = 11–365) with 34 (68%) studies using an intervention length less than or equal to 105 days, and the remaining 16 (32%) studies employing a longer intervention duration. Comparators were always hypothesized to be passive; however, in one case, it (olive oil) proved to be active (Stevens et al. 2003).

#### 5.1.3 Excluded Studies

Altogether, 148 studies were excluded when checking for eligibility criteria. The full list of these studies, together with the reason for their exclusion, can be found in the Supporting Information S1 to this article. Out of the 148 studies, 56 had an ineligible design/article type (e.g., review, commentary), and 22 did not collect outcome data relevant for the purposes of this review. In 22 cases, the sample was not characterized by above‐normal aggression/antisocial behavior either at baseline or at intervention‐end. Further, authors of 14 studies collected but did not report eligible outcome data (e.g., reported only the total score of a scale, while only a subscale score would have been relevant), and we were not able to contact them or the authors were unable/unwilling to provide the data we needed for the purposes of this review. Fourteen studies used an intervention not eligible for our purposes (e.g., intervention with a challenge paradigm – such as a one‐time, high‐sugar food intake – instead of aiming to optimize the nutritional status of participants). In addition, nine studies were ongoing/unpublished at the time of data synthesis, while an additional nine studies employed a sample with a mean age of 25 years or above. Finally, two studies were published in a language not spoken by the study team (one paper in Russian and another one in Dutch).

### 5.2 Risk of Bias in Studies Included in the Quantitative Synthesis

As required by the revised Cochrane Risk of Bias tool, risk of bias was assessed on the outcome and not on the study‐level resulting in a larger number of assessments than the number of included studies. Only a small number (*n* = 12, 16.7%) of effect sizes was associated with low risk of bias, the majority was characterized by moderate (*n* = 34, 47.2%) or high (*n* = 26, 36.1%) risk of bias. Detailed (domain‐level) risk of bias assessment results for each considered effect size metric can be found in the Supporting Information S1 (see Worksheet “All extracted data”).

### 5.3 Quantitative Synthesis Regarding the Effects of Interventions

#### 5.3.1 Effects on Aggression

##### 5.3.1.1 Broad‐Spectrum Nutritional Interventions

**Figure 3:**
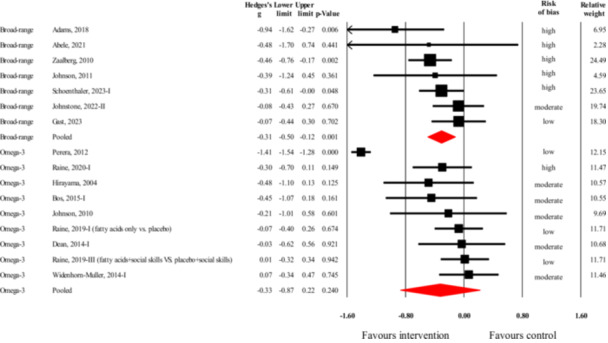
Forest plot of the effect sizes from studies investigating the efficacy of broad‐spectrum nutritional interventions or omega‐3 fatty acid supplementation in reducing aggression (black rectangles represent individual studies, while red diamonds represent pooled effects for the two different types of interventions).

Of the 18 studies on aggression as the outcome, seven studies (Figure 3) investigated an intervention with a broad nutritional target (i.e., broad‐range nutrient supplement or significant changes in overall nutritional composition of diet). The overall number of participants in these studies was 797. The data in these studies showed a small but statistically significant treatment benefit (*g* = −0.31, 95% CI = −0.50 to −0.12, *z* = −3.22, *p* = 0.001). Sensitivity analysis removing outcomes judged to be at high risk of bias left only two studies to pool (Gast et al. 2023; Johnstone et al. 2022). Based on this analysis, the treatment effect became negligibly small and statistically non‐significant (*g* = −0.07, 95% CI = −0.33 to 0.18, *z* = −0.57, *p* = 0.567),^2^ but still favored the intervention.

For the analyses including all seven studies, the *Q*‐value was 7.9 with six degrees of freedom (*p* = 0.249), suggesting no significant heterogeneity across these studies. The *I* ^2^ was 23.6% indicating that about three‐fourths of the variance in observed effects reflects sampling error rather than variance in true effects. The 95% prediction interval for these studies was wide (−0.71 to 0.09) and encompassed a moderately large treatment benefit, the possibility of no effect, and negligible treatment harm. Publication bias was not analyzed due to the low number of studies in this group. Subgroup analyses showed no between‐study differences across study design (*Q* = 0.07, *p* = 0.792), population (*Q* = 0.56, *p* = 0.456), age group (*Q* = 0.06, *p* = 0.804), or intervention duration (*Q* = 0.02, *p* = 0.903). Detailed, subgroup‐level results of these analyses can be found in the Supporting Information S1. Effect sizes did differ though between studies with different proportions of males (*Q* = 4.53, *p* = 0.033): treatment effect was larger in studies with 80+% of males (*g* = −0.44, 95% CI = −0.63 to −0.24, *z* = −4.33, *p* < 0.001) than in studies where the proportion of males was less than 80% (*g* = −0.09, 95% CI = −0.34 to 0.16, *z* = −0.72, *p* = 0.473). Finally, dietary modification (*g* = −0.69, 95% CI = −1.17 to −0.21, *z* = −2.81, *p* = 0.005) was remarkably^3^ more effective (*Q* = 2.78, *p* = 0.096) than nutritional supplementation (*g* = −0.25, 95% CI = −0.44 to −0.06, *z* = −2.63, *p* = 0.009).

##### 5.3.1.2 Omega‐3 Fatty Acid Supplementation

Of the 18 studies on aggression as the outcome, nine studies (Figure 3) investigated supplementation with omega‐3 PUFAs (total *N* = 706). The meta‐analysis of these studies showed a small and statistically non‐significant effect (*g* = −0.33, 95% CI = −0.87 to 0.22, *z* = −1.18, *p* = 0.240) favoring the intervention. Sensitivity analysis by removing one study with high risk of bias (Raine et al. 2020) resulted in no change in these results (*g* = −0.33, 95% CI = −0.93 to 0.27, *z* = −1.07, *p* = 0.284).^4^

For the analyses including all nine studies, the *Q*‐value was 156.0 with eight degrees of freedom (*p* < 0.001), suggesting significant heterogeneity across these studies. The *I* ^2^ was 95% indicating that the vast majority of the variance in observed effects reflects variance in true effects and not sampling error. The 95% prediction interval for these studies was quite wide (−2.31 to 1.66; encompassing large treatment benefit, no effect, or large treatment harm), warranting further subgroup analyses. Subgroup analyses showed no between‐study differences across study design (*Q* = 0.13, *p* = 0.714), population (*Q* = 0.13, *p* = 0.938), proportion of males (*Q* = 1.15, *p* = 0.564), age group (*Q* < 0.01, *p* = 0.971), or intervention duration (*Q* = 0.05, *p* = 0.831) suggesting that the source of heterogeneity was caused by other, non‐investigated variables. Detailed, subgroup‐level results of these analyses can be found in the Supporting Information S1.

We were also interested in the efficacy of nutritional interventions at follow‐up (about 3 months after discontinuing intervention). In this group of studies, there were three outcome indicators where both an intervention‐end and a follow‐up value were considered. In these cases, the effect size was negligible and non‐significant both at intervention‐end (*g* = −0.10, 95% CI = −0.30 to 0.11, *z* = −0.93, *p* = 0.351) and at follow‐up (*g* = −0.13, 95% CI = −0.55 to 0.30, *z* = −0.57, *p* = 0.566) with no statistically significant difference between these effect sizes (*Q* = 0.02, *p* = 0.904).

##### 5.3.1.3 Amino Acid Supplementation

The remaining two studies (total *N* = 84) assessing aggression as the outcome investigated different amino acids; therefore, the decision was made not to pool them. One of the two studies investigated the effects of l‐tryptophan (Ghose 1983), showing zero effect (*g* = 0.00, 95% CI = −0.89 to 0.89, *z* = 0.00, *p* = 1.00). The other study investigated histidine supplementation (Nishijo et al. 2021), the results of which indicated large but statistically non‐significant treatment harm (*g* = 0.90, 95% CI = −0.27 to 2.07, *z* = 1.51, *p* = 0.131). Both of these outcomes were characterized by high risk of bias.

#### 5.3.2 Effects on Antisocial Behavior

##### 5.3.2.1 Broad‐Spectrum Nutritional Interventions

**Figure 4:**
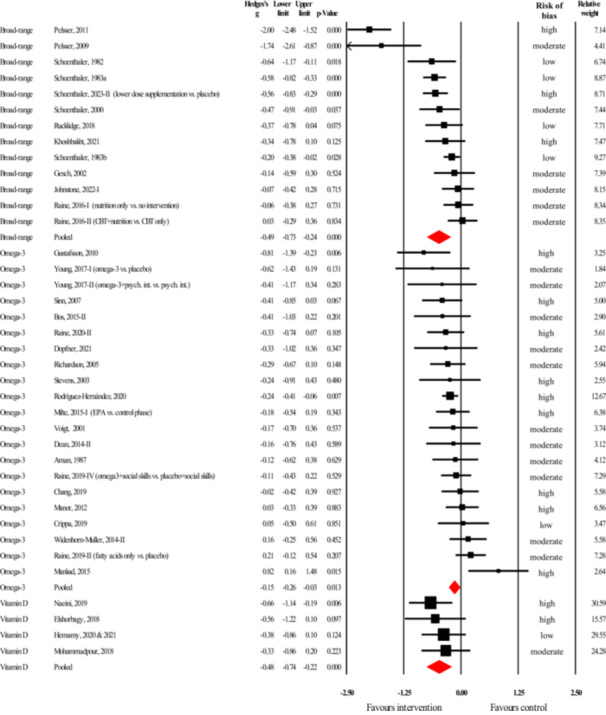
Forest plot of the effect sizes from studies investigating the efficacy of broad‐spectrum nutritional interventions, omega‐3 fatty acid, or vitamin D supplementation in reducing antisocial behaviors (black rectangles represent individual studies, while red diamonds represent pooled effects for the three different types of interventions).

Of the 43 studies on antisocial behavior as the outcome, 13 studies (total *N* = 2109) investigated an intervention with a broad nutritional target (i.e., broad‐range nutrient supplement or significant changes in overall nutritional composition of diet). The data in these studies (Figure 4) showed a small‐to‐moderate, statistically significant effect in favor of the intervention (*g* = −0.49, 95% CI = −0.73 to −0.24, *z* = −3.87, *p* < 0.001). Sensitivity analysis by removing the three outcomes with high risk of bias (Khoshbakht et al. 2021; Pelsser et al. 2011; Schoenthaler et al. 2023) resulted in a reduction in treatment effect, which became smaller but still statistically significant (*g* = −0.32, 95% CI = −0.51 to −0.13, *z* = −3.22, *p* < 0.001).^5^

For the analyses including all 13 studies, the *Q*‐value was 76.8 with 12 degrees of freedom (*p* < 0.001), suggesting significant heterogeneity across these studies. The *I* ^2^ was 84% indicating that the vast majority of the variance in observed effects reflects variance in true effects and not sampling error. The 95% prediction interval for these studies was wide (−1.42 to 0.44), incorporating the possibility of large treatment benefit, no difference, or moderate treatment harm, warranting further subgroup analyses. Subgroup analyses showed no between‐study differences across study design (*Q* = 0.14, *p* = 0.708), age group (*Q* = 0.38, *p* = 0.825), or intervention duration (*Q* = 1.00, *p* = 0.318). Detailed, subgroup‐level results for these analyses can be found in the Supporting Information S1. Effect sizes did differ though between studies with different proportions of males (*Q* = 8.20, *p* = 0.004): treatment effect was much larger in studies with 80 + % of males (*g* = −0.80, 95% CI = −1.22 to −0.39, *z* = −3.77, *p* < 0.001) than in studies where the proportion of males was less than 80% (*g* = −0.17, 95% CI = −0.29 to −0.04, *z* = −2.66, *p* = 0.008). Differences also emerged in relation to population (*Q* = 4.74, *p* = 0.093): treatment effect was substantially larger in intervention recipients with a diagnosis not primarily defined by aggression/antisocial behavior (*g* = −0.86, 95% CI = −1.60 to −0.12, *z* = −2.29, *p* = 0.022) than in offenders (*g* = −0.41, 95% CI = −0.62 to −0.20, *z* = −3.85, *p* < 0.001) or individuals with a diagnosis primarily characterized by aggression/antisocial behavior (*g* = −0.13, 95% CI = −0.40 to 0.14, *z* = −0.93, *p* = 0.352). Finally, the studies investigating dietary modification (*g* = −0.86, 95% CI = −1.36 to −0.35, *z* = −3.34, *p* = 0.001) showed remarkably larger effects (*Q* = 5.18, *p* = 0.023) than studies on nutritional supplementation (*g* = −0.23, 95% CI = −0.42 to −0.04, *z* = −2.40, *p* = 0.017).

We were also interested in the efficacy of nutritional interventions at follow‐up (about 3 months after discontinuing intervention). In this group of studies, there were again three outcome indicators where both an intervention‐end and follow‐up value could be calculated. In these cases, the effect size was negligible and non‐significant both at intervention‐end (*g* = −0.13, 95% CI = −0.40 to 0.14, *z* = −0.93, *p* = 0.352) and at follow‐up (*g* = −0.12, 95% CI = −0.35 to 0.10, *z* = −1.06, *p* = 0.289), with no statistically significant difference between these effect sizes (*Q* < 0.01, *p* = 0.972).

**Figure 5:**
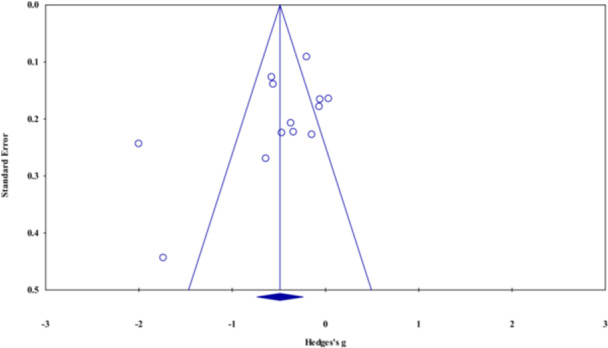
Funnel plot of effect size metrics from studies investigating the efficacy of broad‐spectrum nutritional interventions in reducing antisocial behaviors.

The funnel plot of the effect size metrics from studies investigating the efficacy of broad‐spectrum nutritional interventions in reducing antisocial behaviors is displayed in Figure 5. Visual inspection of this plot suggests some asymmetry, and more specifically, a potential small study effect. The Egger's test was conducted to investigate this further. This analysis did not provide a statistically significant indication of publication bias (intercept = −2.96, 95% CI = −7.02 to 1.10, *p* = 0.14) and using the trim and fill procedure left the effect size from the main analysis unchanged.

##### 5.3.2.2 Omega‐3 Fatty Acid Supplementation

Of the 43 studies on antisocial behavior as the outcome, 21 effect size metrics were related to the effects of omega‐3 PUFA supplementation (total *N* = 2081). The data in these studies (Figure 4) showed a very small but statistically significant treatment benefit (*g* = −0.15, 95% CI = −0.26 to −0.03, *z* = −2.47, *p* = 0.013). Sensitivity analysis by removing the 9 indicators with high risk of bias resulted in a further slight decrease in effect size (*g* = −0.09, 95% CI = −0.23 to 0.05, *z* = −1.29, *p* = 0.196),^6^ incorporating the possibility of no difference. For the analyses including all 21 effect sizes, the *Q*‐value was 28.5 with 20 degrees of freedom (*p* = 0.098) suggesting some but not statistically reliable heterogeneity across these studies. The *I* ^2^ was 29.8% indicating that only about one‐third of the variance in observed effects reflects variance in true effects and so the majority is sampling error. The 95% prediction interval for these studies was −0.46 to 0.17, incorporating the possibility of small‐to‐moderate treatment benefit, no difference, or small treatment harm.

Subgroup analyses showed no between‐study differences across study design (*Q* = 0.76, *p* = 0.682), population (*Q* = 1.60, *p* = 0.449), proportion of males (*Q* < 0.01, *p* = 0.979) or age group (*Q* = 0.86, *p* = 0.355). Detailed, subgroup level results of these analyses can be found in the Supporting Information S1. Effect sizes did differ though between studies by intervention length (*Q* = 4.65, *p* = 0.031): surprisingly, treatment effect was larger in cases of intervention lengths of 105 days or less (*g* = −0.24, 95% CI = −0.36 to −0.13, *z* = −4.19, *p* < 0.001) than in studies with longer intervention duration (*g* = −0.01, 95% CI = −0.19 to 0.17, *z* = −0.10, *p* = 0.922). It is of note though that the number of studies with high risk of bias was almost double (55%) in the latter group than among those with shorter intervention duration (30%), raising the possibility that this counterintuitive finding is an artifact, rather than a true pattern.

To examine the efficacy of nutritional interventions at follow‐up, three studies/study arms were considered. The effect size was negligible and non‐significant both at intervention‐end (*g* = −0.06, 95% CI = −0.36 to 0.24, *z* = −0.39, *p* = 0.697) and at follow‐up (*g* = −0.11, 95% CI = −0.43 to 0.21, *z* = −0.67, *p* = 0.501) with no statistically significant difference between these effect sizes (*Q* = 0.05, *p* = 0.829).

**Figure 6:**
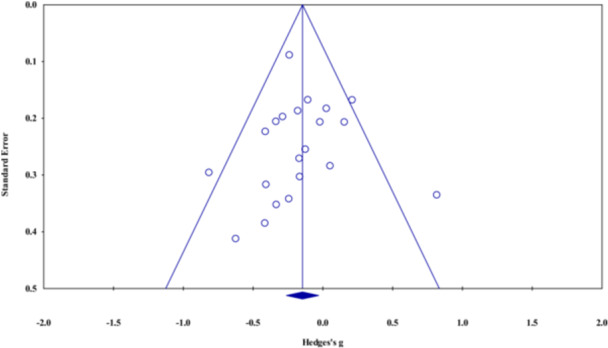
Funnel plot of the effect size metrics from studies investigating the efficacy of omega‐3 fatty acid supplementation in reducing antisocial behaviors.

The funnel plot of the effect size metrics from studies investigating the efficacy of omega‐3 fatty acid supplementation in reducing antisocial behaviors is displayed in Figure 6. Visual inspection indicated only very minor asymmetry. To further assess the small‐study effect and potential publication bias, the Egger's test was conducted. This analysis did not provide any indication for publication bias (intercept = −0.09, 95% CI = −1.52 to 1.34, *p* = 0.897). Using the trim and fill procedure, three studies were added to this pool of effect sizes, resulting in an effect size (*g* = −0.11, 95% CI = −0.23 to 0.01) somewhat smaller than in the main analyses (*g* = −0.15, 95% CI = −0.26 to −0.03).

##### 5.3.2.3 Vitamin D Supplementation

Of the 43 studies on antisocial behavior as the outcome, four studies (total *N* = 226) investigated the effects of vitamin D supplementation. The data in these studies (Figure 4) showed a small‐to‐moderate, statistically significant treatment benefit (*g* = −0.48, 95% CI = −0.74 to −0.22, *z* = −3.61, *p* < 0.001). Sensitivity analysis by removing the two indicators with high risk of bias (Elshorbagy et al. 2018; Naeini et al. 2019) resulted in a smaller but still significant treatment effect (*g* = −0.36, 95% CI = −0.7 to 0.00, *z* = −1.96, *p* = 0.05).^7^

The *Q*‐value for the entire group of four studies was 1.1 with three degrees of freedom (*p* = 0.774) suggesting no significant heterogeneity across these studies. The *I* ^2^ was estimated as zero indicating that all variance in observed effects reflect sampling error. The 95% prediction interval for these studies was −0.46 to 0.17, incorporating the possibility of small‐to‐moderate treatment benefit, no difference, or small treatment harm. Publication bias was not analyzed due to the low number of studies in this group. These studies were homogenous in terms of design, population, age group, and intervention duration; therefore, the only relevant subgrouping was based on proportion of males. This subgroup analysis showed no statistically significant difference between studies above versus below 80% of male participation (*Q* = 0.81, *p* = 0.368). Details of this analysis can also be found in the Supporting Information S1.

##### 5.3.2.4 Other Nutritional Interventions

**Figure 7:**
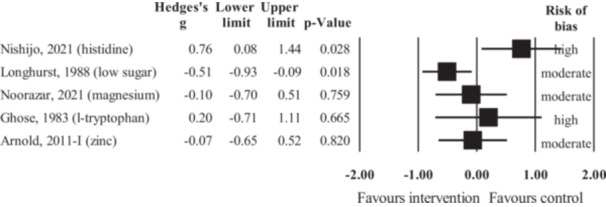
Forest plot of individual effect sizes from studies investigating the efficacy of different nutritional interventions (specified next to first author and publication year) in reducing antisocial behaviors.

The remaining five studies (total *N* = 308) that assessed antisocial behavior as the outcome investigated the effects of reduced sugar intake (Longhurst and Mazer 1988), or the effects of magnesium (Noorazar et al. 2021), zinc (Arnold et al. 2011), l‐tryptophan (Ghose 1983), and histidine (Nishijo et al. 2021) supplementation. Considering the differences across these interventions, the decision was made not to pool their results; instead, the outcomes were considered individually (Figure 7). Out of the five studies, only two showed a statistically significant treatment effect: the study on histidine supplementation indicated treatment harm (moderate‐to‐large effect), while the study on reduced‐sugar diet showed treatment benefit (moderate effect). The effect sizes for the other three studies were negligible.

#### 5.3.3 Effects on Offending

**Figure 8:**
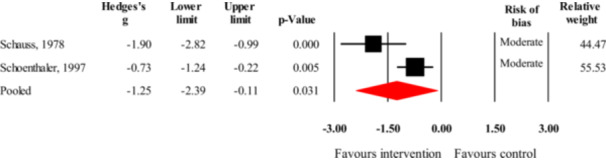
Forest plot of the effect sizes from studies investigating the efficacy of broad‐spectrum nutritional interventions in reducing criminal offending (rectangles represent the individual studies, while the diamond represents the pooled effect).

We were able to enter only two studies (total *N* = 117) on offending into the meta‐analysis (Figure 8). Both studies used a broad‐spectrum nutritional intervention: one employing complex dietary improvement in a probational setting (Schauss and Schauss 1978), while the other used broad‐range micronutrient supplementation in the prison setting (Schoenthaler et al. 1997). The results showed a strong and statistically significant treatment effect (*g* = −1.25, 95% CI = −2.39 to −0.11, *z* = −2.15, *p* = 0.031). The *Q*‐value was 4.79 with one degree of freedom (*p* = 0.029) suggesting significant heterogeneity across these two studies. The *I* ^2^ was 79% indicating that most of the variance in observed effects reflect a difference in true effects rather than sampling error; however, statistical heterogeneity was due to magnitude rather than direction of effect. Prediction interval was not calculated due to the low number of studies included in this analysis. While this is not a true subgroup analysis due to only one study being in each group, we compared the included studies' effect sizes as one of them investigated diet change, while the other studied nutritional supplementation. In line with the prior results (cf. subgroup analyses in 5.3.1.1 and 5.3.2.1), the study on diet change (*g* = −1.90, 95% CI = −2.82 to −0.99, *z* = −4.07, *p* < 0.001) reported a significantly larger effect (*Q* = 4.79, *p* = 0.029) than the one on nutritional supplementation (*g* = −0.73, 95% CI = −1.24 to −0.22, *z* = −2.82, *p* = 0.005). Publication bias was not analyzed either due to the low number of studies included here. Both of these indicators were characterized by moderate risk of bias; therefore, sensitivity analyses were not needed.

### 5.4 Qualitative Synthesis Regarding Implementation Barriers and Facilitators

Considering the need for practical usability of the findings, beyond the standard quantitative data collection and synthesis on intervention efficacy, we also collected data on implementation barriers and facilitators. We identified different stages of implementation and organized implementation‐related information corresponding to these five stages: (1) Awareness regarding the relevance of and interest in nutritional interventions among relevant decision makers and the target group; (2) Access to nutritional interventions; (3) Specific characteristics of nutritional interventions influencing implementation; (4) Consumer compliance with nutritional interventions; and (5) Intervention‐interfering behaviors or physiological processes. All these stages were considered separately for dietary manipulation versus nutritional supplementation/fortification due to the significant implementation‐related differences between these nutritional intervention types. A summary of these findings can be found in Table 1.

**Table 1:** Summary on the implementation barriers and facilitators of nutritional interventions.

	Dietary modification		Nutritional supplementation/fortification	
	Implementation barriers	Implementation facilitators	Implementation barriers	Implementation facilitators
Awareness and interest in the target population and other stakeholders	Negative consumer attitudes about healthy foods Heavy reliance on social media and internet for seeking nutrition‐related information Limited awareness on importance of nutrition regarding mental and behavioral health Negative attitudes and resistance from food providers and parents due to perceived burden of nutrition policies	Increased consumer demand for healthy foods through customer engagement and feedback on food choices Networking among organizations to remove knowledge gaps Seeking information from reputable sources Tailored education initiatives to increase awareness on nutrition policies Clear communication and collaboration between stakeholders and food providers	Limited awareness and inaccurate information in the target population Limited knowledge base and motivation to promote supplementation among professionals	Educational initiatives in the target population Obligatory (university) or incentivized (post‐gradual) educational initiatives for professionals Community engagement
Access to nutritional interventions	Physical and geographical limitations (limited storage capacity, food spoilage) Higher operational costs, contractual obligations Fear of profit loss at organizational and retailer level Voluntary or unofficial policies not being taken seriously Insufficient funding and human resources, limited infrastructure, lack of support	Alternative product storage and conserving methods Locally sourced fresh foods Collaboration between policymakers and food providers to minimize operational costs Allocation of funding for staff training and equipment Collaboration between suppliers and food providers to renegotiate contracts Consistent and clear mandatory policy endorsed by government or workplace management Community engagement and collaboration between suppliers and food providers	Distance and travels costs to supplement distribution points Costs of nutritional supplements Shortage of supplements in certain societies	Direct distribution by healthcare professionals Door‐to‐door distribution by organizations or online purchase by individual consumers Free/subsidized/health‐insurance‐covered provision of supplements
Nutritional intervention characteristics	Unclear definition of a “healthy diet” (influenced by individual characteristics, cultural variations, inconsistent guidelines) Unclear guidance regarding healthy quantities in a diet Difficulties assessing the right duration of interventions for long‐term benefits	Personalized nutritional interventions considering cultural variability, individual needs Use of visual dietary guides	Uncertainty regarding which nutrients to target in which proportions Uncertainty regarding optimal dosage to use Uncertainty regarding optimal duration of supplementation to reach maximal effect Regulation‐related challenges with higher‐dose supplements Medication interactions	Further research on optimal target nutrients, dosages, and duration of interventions Training and education for psychiatric medication prescribers on nutritional interventions Modified treatment approaches
User compliance	Low quality, unappealing taste and appearance of certain healthy foods Customer resistance and negative perceptions Competing priorities, lack of willingness to change behaviors related to unhealthy eating Difficulties with adjusting existing habits and lack of time to experiment with new ones Social pressure and lack of support (e.g., from family, friends, peers, or healthcare professionals) Picky eating behaviors in children Side‐effect burden from certain specific diets	Use of local and home‐made products with fewer chemicals Offering taste‐testing sessions and food samples before full implementation Customer engagement and informative messages to raise awareness of the importance of healthy foods Gradually changing the frequency of unhealthy foods to healthy foods Building community and social support structures (e.g., family, peers, friends, online communities, cooking classes) Building support network of health professionals Applied behavioral analysis for picky eating	Health status influencing compliance Health provider's lack of knowledge regarding intervention Inconvenience and undesired distribution settings Lack of social support (e.g., discouragement from family members) Participant forgetfulness Swallowability of capsules and taste‐related issues Challenges with dosages and frequency of intervention Minor but existing side‐effect burden from supplements	Use of community networks to convey proper nutrition‐related information Online purchase of supplements; flexibility in appointments, at‐home visits; use of school and community settings for intervention Support from healthcare providers; family and community engagement and support Use of incentives Development of a reminder system (phone app, text messages, regular check‐in with professionals) Pill‐swallowing techniques, alternative methods and flavoring of supplementation Easy‐to‐follow supplementation regime Feedback based on reliable measures of compliance (e.g., blood work)
Intervention‐interfering processes	Gut health issues (e.g., low microbiota diversity) Use of medications interfering with optimal absorption Low nutritional quality of foods Individual genetic and metabolic differences resulting in above‐average nutrient needs	Prebiotic and probiotic diet to improve gut health Supplementation with above‐average nutrient doses Emphasizing intake of higher quality foods and lowering use of highly processed ones Multidisciplinary approach to managing individual health conditions	Concurrent illnesses influencing intervention effectiveness Use of medications interfering with optimal absorption Genetic variations and polymorphisms interfering with optimal absorption Substance use interfering with the efficacy of supplementation	Refined supplements (e.g., mercury‐free fish oil) Balancing nutrient supplementation to overcome nutrient deficiencies Addressing substance use first/in parallel Education for clinicians about drug‐nutrient interactions

#### 5.4.1 Dietary Manipulation

##### 5.4.1.1 Stakeholder Awareness and Interest

###### 5.4.1.1.1 Implementation Barriers

One key area of consideration concerns awareness of healthy food and nutrition guidelines in schools and other institutions. A systematic review on school‐based food policies aiming to improve youths' food consumption through modifications to the food environment such as through the provision of nutritious foods and marketing restrictions for unhealthy ones indicated that stakeholders often lack knowledge and understanding of healthy food policy requirements due to the lack of clarity of the policy guidelines or lack of communication (Ronto et al. 2020). Such a barrier has been highlighted in school settings in low‐ and middle‐income countries as well (Meshkovska et al. 2023). In publicly funded institutions, it has been found that there was a disparity between individuals' understanding of what is considered “healthy” and what the organization's specific policy guidelines stated, while at the governance level, there was a lack of motivation to enforce guidelines or take them seriously because they were voluntary (Rosewarne et al. 2022).

The above barriers to implementation may be influenced by other individual‐level factors such as mental health status. For example, one study that investigated dietary habits and knowledge among adults with severe mental health issues found that they were less likely to consider diet and exercise as a relevant part of their lifestyles and that their lower levels of knowledge about nutrition were often associated with their poor nutritional habits and physical activity, compared to adults without a severe mental illness (Osborn et al. 2007).

Perceptions about dietary changes can also affect interest in adopting such interventions. A systematic review that focused on food‐environment policy implementation noted that at the individual level, consumers often had perceived notions that unhealthy foods are more popular and that healthy options were more expensive and thus less appealing (Middel et al. 2019). A review of healthy food policy in public sector workplaces reported that customers' perceived, expected, or actual negative reactions to removal of or restrictions on unhealthy items were disheartening to managers and food service staff at all levels and reinforced their negative perception of implementation (Rosin et al. 2024).

Food providers, on the other hand, often felt that the responsibility of managing consumer expectations was burdensome, and that changing behaviors or forcing individuals to eat healthily should not be placed on their shoulders (Rosin et al. 2024). The implementation is often experienced as being overwhelming or confusing by individual food providers, due to the stakeholders' limited nutritional background and lack of communication efforts (Rosin et al. 2024). This view is supported by findings that in schools, staff had negative perceptions of policies focused on healthy initiatives because they felt burdened about the additional responsibilities of having to implement them (Ronto et al. 2020). Schools also often prioritize academic performance over the implementation of nutritional policies for their students due to the concern that healthy policies might cause dissatisfaction among students and interfere with their learning outcomes, and parents may not agree with the policies restricting food choice options (Ronto et al. 2020). Additionally, a more prescriptive than collaborative approach towards healthy food policies can result in negative attitudes towards implementation in general (Rosin et al. 2024).

###### 5.4.1.1.2 Implementation Facilitators

At the individual level, the primary facilitators are consumer attitudes towards healthy products. This may be influenced by obtaining information related to nutrition from reliable sources. A study on nutrition and physical activity in young people attending mental health services reported that most young people found it preferable to obtain their nutrition‐related information from reputable healthcare providers such as general practitioners and dietitians (Mawer et al. 2022). An investigation of facilitators and barriers to implementation of nutritional interventions in early childcare settings further revealed that children's positive reactions to the intervention influenced educators' positive attitudes towards it, as well as their beliefs and motivations for engaging with the intervention, which constitute critical factors in adoption and implementation success (Asada et al. 2023). Positive consumer feedback, especially on initial changes to usual food offerings, can additionally increase food providers' willingness to implement a new policy. This can be done by conducting customer surveys before and during implementation to identify consumer preferences, such as desirable healthy options (Rosin et al. 2024).

Other facilitators that have been identified include (1) networking with organizations to help minimize the disparity between consumers' understanding of healthy foods and information provided in the nutrition guidelines (individual level), (2) an improvement in upper management's support (organizational level), (3) staff buy‐in/support towards initiatives (community level), (4) positive relationship with suppliers/retailers and having suppliers/retailers who are motivated to provide healthier foods (supplier level), and finally, (5) integrating policy/guidelines into contractual obligations with suppliers and retailers (governance level) (Rosewarne et al. 2022).

Furthermore, having tasty and acceptable food and drink in place before limiting or removing popular unhealthy options, especially in the early stages of implementation, can allow consumers to make their own food choices while gradually exposing them to healthier options using active engagement methods (Rosin et al. 2024). Multi‐component marketing strategies such as regular informative and promotional messages directed at consumers has also been suggested to improve awareness, support, and, ultimately, demand for healthier options (Rosin et al. 2024).

It has also been found in a school environment that (1) creating diverse menus that were conscious of dietary restrictions or specific preferences, (2) providing proper health education to children or incorporating programs dedicated to nutritional education as part of the overall academic health curriculum, (3) making easier‐to‐follow guidelines that are not burdensome to staff, and (4) giving information to teachers regarding the negative consequences of unhealthy foods such as damage that sugar‐sweetened beverages and processed foods cause all served as facilitators of nutrition‐related actions (Meshkovska et al. 2023; Ronto et al. 2020). The use of videos and diverse educational activities as part of these types of actions was also a potential facilitator (Meshkovska et al. 2023).

##### 5.4.1.2 Access

###### 5.4.1.2.1 Implementation Barriers

A study by Middel et al. (2019) found that fluctuating demands for healthier options, along with the geographical limitations of shipping goods, can potentially hinder the supply of healthy food items. The same authors noted that physical limitations such as limited storage capacity for fresh products, high operational costs, as well as a waste of fresh product due to spoilage were potential barriers to access. Furthermore, limited available space and impracticality in the physical arrangements of layouts in institutions often pose challenges in the implementation of healthy food policies, as suppliers may have to make significant changes to accommodate the healthy food offerings (e.g., making changes to shelving and equipment) (Rosin et al. 2024).

Another study focusing on the policy of implementing dietary interventions in publicly funded institutions determined that the existence of multiple site locations and methods of food provision were barriers that influenced implementation at the organizational level (Rosewarne et al. 2022). Insufficient funding given the high costs associated with healthier foods, limited infrastructure (e.g., a lack of canteen and kitchen facilities that hinder the ability to prepare healthy lunches), and limited administrative time, all posed significant obstacles to the development of healthier food environment policies (Meshkovska et al. 2023; Ng et al. 2022; Ronto et al. 2020). Additionally, due to minimum product order requirements, smaller food providers may not have enough buying authority to purchase healthier, less popular options, and there may be restrictions in contracts that limit product sourcing from various suppliers (Rosin et al. 2024).

###### 5.4.1.2.2 Implementation Facilitators

Strategies to increase community engagement (e.g., organizing cooking demonstrations), self‐sustaining fresh produce, and improvements in menu planning, can all help facilitate access to healthier food options (Ng et al. 2022). Moreover, practices utilizing methods of conserving products such as freezing products prone to spoilage and methods of product stocking, such as transforming near‐expiry products into daily offering items alleviated the problem of food waste due to product spoilage (Middel et al. 2019).

To overcome logistical challenges, governments could provide financial support, which could also support training programs for staff, which were shown to improve the implementation of healthy food policies in schools (Ng et al. 2022). Stakeholders should also consider taxing unhealthy food items and from the generated revenue, subsidize healthy but more costly food options in as many settings as possible (Mulder and Rucklidge 2023). Furthermore, implementation was more successful when in addition to the government, local farmers and cooperatives invested their funds in participating in food provision programs, such as purchasing trucks to deliver food to schools (Meshkovska et al. 2023). To overcome the challenges associated with contractual restrictions, food providers may collaborate and renegotiate their contracts with suppliers to stock healthier items. Lastly, a culture focused on innovation and experimentation is considered a potential facilitator: if a retailer has close ties to the community, its participation in efforts to test interventions involving dietary improvements is more likely (Middel et al. 2019).

##### 5.4.1.3 Intervention Characteristics

###### 5.4.1.3.1 Implementation Barriers

One important barrier to improving diet is that the definition of a “healthy diet” is often unclear (e.g., what exactly fits into it and what does not) and may vary across cultural contexts, individual needs, and even dietary guidelines (Nemec 2020). Dietary recommendations often do not consider these cultural variations, making it difficult for diverse populations to adhere to suggested diets. Another discrepancy is evident in that the US dietary guidelines describe a healthy diet as one that is comprised of whole grains, fruits and vegetables, fat‐free and low‐fat dairy products, lean meats, and foods low in fat, sodium, and added sugars. These guidelines are directed towards the general public (ages 2 years and above) (Branscum and Sharma 2020). In contrast, the World Health Organization (WHO) describes healthy diets as dietary patterns that balance energy to maintain a healthy weight, limit energy from fats (consuming more unsaturated fats), and reduce intake from sugars and salts (e.g., using iodized salts). If all foods can generally be part of a healthy diet when consumed in moderation, then identifying what dietary manipulations should include constitutes a challenge (Branscum and Sharma 2020).

###### 5.4.1.3.2 Implementation Facilitators

Culturally tailored dietary interventions may need to be considered. This includes placing a larger focus on cultural humility (i.e., understanding participants' cultural beliefs as well as cultural dimensions of diet) and integrating this into the administration of dietary interventions (Zeda 2024).

##### 5.4.1.4 User Compliance

###### 5.4.1.4.1 Implementation Barriers

The overall drop‐out rate from dietary manipulation in the studies included in this review was 11.62% on average (with a standard deviation of 16.42%). Of the data collected from our included studies, the healthy gluten‐free, casein‐free, soy‐free diet had the lowest compliance rate (Adams et al. 2018). Side effects may affect compliance; for instance, some participants reported temporary mild nausea, and one child had loose stools as a side‐effect of this specific diet. In other studies, side effects were similar and included irregular bowel movements, stomach aches, vomiting, night wakings, and decreased appetite (Johnson et al. 2011). These complaints were rare though (or completely missing, cf. Pelsser et al. 2011), and due to their non‐specific nature, it is hard to know whether they were indeed related to the intervention or other factors (e.g., mild infection). Further, when unwanted effects were monitored in both study arms, the control and intervention groups often did not differ significantly (e.g., Johnson et al. 2011). However, if these minor side effects are perceived by participants or caregivers as related to the dietary intervention, they may reduce compliance.

Besides side effects, a study investigating the challenges with implementation of nutrition‐related actions in school settings found that the quality of food was a barrier to compliance, where food was generally of low quality (Meshkovska et al. 2023). Furthermore, the unappealing taste and appearance of healthy food were common barriers to compliance. It has been suggested that many individuals do not want to accept healthier options and their dissatisfaction with restrictions on unhealthy items often leads food providers to justify non‐compliance (Rosin et al. 2024).

Another barrier to user compliance concerns competing priorities (e.g., family, work, or hobbies), and difficulties adjusting existing habits to ones that are favorable to a healthier diet (Domosławska‐Żylińska et al. 2024). Factors such as emotional eating, food cravings, and hunger have also been identified to hinder adherence to healthy diets in interventions (Deslippe et al. 2023), while unhealthy eating patterns in the family and difficulties in avoiding unhealthy foods in family gatherings and community settings can be barriers to implementing healthy eating (Domosławska‐Żylińska et al. 2024). In addition, women were more likely to indicate factors such as a lack of support from friends and family, and healthcare professionals (e.g., physicians) as a hindrance to practicing a healthy diet (Domosławska‐Żylińska et al. 2024). Moreover, negative attitudes towards interventions involving dietary manipulation such as previous failure to change eating habits (Deslippe et al. 2023) or selective, “picky” eating (aka Avoidant Restrictive Food Intake Disorder) especially in children with autism, can be major barriers to compliance with dietary changes.

###### 5.4.1.4.2 Implementation Facilitators

Findings from Meshkovska et al. (2023) suggest that a potential facilitator to the successful implementation of the various nutrition‐related initiatives could be the provision of fresh/healthy products of good quality. The use of home‐made food, local products, and products with fewer chemicals due to organic fertilizers being used was a solution to concerns of taste and agrochemicals. Participants could also be provided with healthy food samples and taste‐testing sessions before the intervention to gather feedback in the early stages of implementation and overcome the negative perceptions around the taste of healthy foods (Rosin et al. 2024). Using consistent, informative messages to engage customers in accepting more nutritious options and raise awareness may also improve compliance with dietary interventions (Rosin et al. 2024).

Strategies that validate the healthier options, such as the inclusion of a clear, consistent, and mandatory policy, promoted by the national or local government and senior workplace management, may help improve adherence, as it becomes a priority to implement. Support structures (both internal and external) throughout implementation, such as positive management attitudes, committed leadership teams, and personalized ongoing assistance from dietitians, are all potential facilitators to compliance (Rosin et al. 2024). Community support and the awareness of health benefits can also be important to help facilitate implementation.

An increased public understanding of the importance of healthy eating in terms of mental and behavioral health is likely to increase both the demand and adherence to healthy food options (Ng et al. 2022). Findings from Deslippe et al. (2023) similarly identified social support and social accountability as major facilitators in adhering to healthy diets; support systems included support from the intervention providers (e.g., by health experts such as physicians, dietitians, trainers, or intervention staff), within the home (e.g., by parents), and outside the home (e.g., by colleagues or peers). Being able to actively participate alongside someone aiming for the same goals may also facilitate healthy eating. With regard to social accountability, factors such as wanting to become a role model, and wanting to change others for the better (e.g., family and friends) also promoted adherence to a healthy diet (Deslippe et al. 2023).

##### 5.4.1.5 Intervention‐Interfering Processes

###### 5.4.1.5.1 Implementation Barriers

Many individual characteristics such as age, sex, weight, health conditions, and lifestyle behaviors, can also influence the effect of dietary interventions. Dietary manipulations have varied effects on behavior due to individual differences, medications used, gut health, and levels of stress and anxiety, which may result in above‐average nutritional needs in many individuals (Rucklidge et al. 2021). For example, various antipsychotic drugs, anticonvulsants, and antidepressants are known to inhibit the absorption of several micronutrients (e.g., vitamin B12, B2, B6, D, K, calcium, etc.), as well as cause depletion in minerals such as magnesium, zinc, or selenium (Pelton et al. 2001). Certain mood stabilizers can also deplete the levels of folic acid, vitamin D, vitamin B6, and vitamin B12, as well as minerals such as copper, selenium, and zinc (Rucklidge et al. 2021). Another factor to note is the possible untreated issues related to an illness that can influence the effectiveness of dietary interventions. For example, many studies suggest that individuals with autism might also have poor digestive health, which can lead to symptoms like diarrhea, constipation, and abdominal pain (Chaidez et al. 2014) and the effectiveness of a dietary intervention could be diminished by such underlying issues if they are not treated first.

Another issue to consider is the nutritional depletion of the foods themselves. Environmental factors (such as climate change, depletion of minerals in soil, or the increasing levels of CO_2_ in the atmosphere) may decrease the density of nutrients found in our foods. This can result in crops that are higher in carbohydrates and lower in proteins and micronutrients (Rucklidge et al. 2021).

###### 5.4.1.5.2 Implementation Facilitators

A diet rich in plant‐based and fermented foods is known to support microbial biodiversity and restore gut health, providing the basis for other dietary interventions aiming to improve mental and behavioral health. For example, one study found that people with an increased consumption of natural sources of pro‐ and prebiotics showed significantly lower levels of anxiety, stress, and neuroticism (Johnson 2020). Another study reported that the higher consumption of natural probiotics through fermented foods related to fewer symptoms of social anxiety in females (Hilimire et al. 2015). Assessing gut health as a factor when implementing dietary interventions can help improve their overall effectiveness. Working on providing foods of higher quality and reducing the intake of highly processed foods is key, when tackling the challenges of poor gut health (Rucklidge et al. 2021).

To resolve the challenges of nutrient deficiencies that arise from certain medications (even with reasonable dietary intake), nutritional supplements with above‐average dosages can be considered. Addressing underlying health issues and individual factors by engaging professionals such as physicians, dietitians, gastroenterologists can help improve the overall effectiveness of dietary interventions.

#### 5.4.2 Nutritional Supplementation

##### 5.4.2.1 Stakeholder Awareness and Interest

###### 5.4.2.1.1 Implementation Barriers

One barrier to implementing nutritional supplementation is the lack of accessible information about nutritional interventions, which is evident across countries. For example, a study in Iran found that only 28% of female high school students received educational information about the iron supplements provided to them as part of a program aimed at addressing iron deficiency anemia (Jafari et al. 2023). Most of the students also did not use the iron supplements despite their free provision, due to their belief that because they did not have a disease, the iron supplementation was completely unnecessary. Similarly, in a South African study, participants aged 18–28 years shared that they were unaware of the purpose of receiving multiple micronutrient supplementation. This lack of understanding resulted in suspicions of the supplementation program among family members, which also contributed to women discontinuing their participation (Silubonde et al. 2022). This was consistent with findings from a study on vitamin A supplementation in Sub‐Saharan African children aged 6–59 months where one of the major barriers to supplementation was a lack of awareness about its availability (Janmohamed et al. 2017).

From the data collected from our included studies in this review, one study particularly highlighted limited nutritional literacy: some prisoners did not have even the most basic knowledge of choosing a healthy diet and some were not aware of what vitamins were at all (Gesch et al. 2002). Moreover, skepticism among health and educational professionals, limited knowledge about vitamin intake recommendations, heavy workloads, and infrequent appointments with the target population contributed to the minimal promotion of vitamin supplement use in a sample of parents and professionals recruited in the United Kingdom (Jessiman et al. 2013).

The level of awareness and knowledge of the use and effectiveness of nutritional supplementation is, in part, influenced by where individuals obtain the information from. A US study on dietary supplement use in children found that most supplements used by children are not suggested by a healthcare professional. Rather, they are suggested by other parents, families, and friends (Bailey et al. 2013). Many adolescents also obtain their information about dietary supplements from internet searches, which are often inaccurate or insufficient (Kärkkäinen et al. 2019). This is confirmed by findings showing that due to obtaining information through unofficial means, such as relying heavily on parents' opinions when addressing their nutritional needs, adolescents end up having several misconceptions about the health benefits of nutritional supplements (O'Dea 2003).

###### 5.4.2.1.2 Implementation Facilitators

Nutrition education initiatives foster a greater understanding of the importance of supplementation and nutrition services (Handiso 2023). One example of this approach is the use of the socio‐scientific issue method (Kärkkäinen et al. 2019). Developers of this method evaluated its effectiveness in improving 7th grade students' knowledge of dietary supplements. The 7‐h‐long health education intervention was delivered through an e‐learning environment and involved three stages: the scenario, the inquiry, and the decision‐making stage, where students acquainted themselves with an imaginary person's nutritional status, looked for information on dietary supplement needs, and applied their knowledge by counseling the imaginary adolescent. This method increased students' knowledge of the rationale for the use of dietary supplements, engaged them in decision‐making, challenged their social and emotional skills, and was positively received by students (Kärkkäinen et al. 2019).

Engaging adolescents in active bidirectional problem‐solving discussions, such as involving facilitators and family members regarding any issue that may have arisen during the implementation of nutrition services further increased adolescents' interest in the intervention (Handiso 2023). Social and community networks were also found to motivate adolescent girls to access nutrition services (Handiso 2023). Improving the general knowledge base of educational and correctional service workers regarding nutrition and supplementation (as part of their university studies and/or postgraduate/continuing education) would also be helpful. Such training for health professionals in the United Kingdom, for example, is required to enhance professionals' understanding of nutritional supplements and to develop strategies for effectively promoting interventions to eligible families (Jessiman et al. 2013).

##### 5.4.2.2 Access

###### 5.4.2.2.1 Implementation Barriers

One study in England collected data from health and children's care professionals, as well as parents to determine why few eligible families were accessing the free vitamin supplements provided by the Healthy Start Scheme (Jessiman et al. 2013). Less than 10% of those eligible used the vitamin supplements. Approximately 20% of eligible families could not access vitamins as they were not formally registered with the specific program. Shedding light on other barriers to supplementation, some parents cited inconvenience and distance from distribution sites. Although the vitamin supplements were often distributed in health and children's centers, many health professionals were unaware of this fact, which contributed to confusion among parents about where to go to collect the vitamins. Delays in processing eligibility applications also prevented mothers from receiving early access to free vitamins for themselves and their young children in this study (Jessiman et al. 2013).

The unavailability of products as a reason for not taking nutritional supplements is also documented in many countries. In Southern Ethiopia, for example, the shortage of iron‐folate supplements meant that the demand for these tablets among adolescent girls was unmet (Handiso 2023). In addition, the economic status of adolescent girls' families impacted their access to nutritional supplements, as tablets were provided at school, which prevented adolescent girls, with a low socioeconomic status, not attending school from receiving the supplementation (Handiso 2023). These findings were corroborated in a study in Sub‐Saharan Africa where although distance to distribution sites was not a significant barrier, household wealth was a potential factor affecting access due to costs associated with traveling to distribution sites (Janmohamed et al. 2017). School‐based supplementation programs may be challenged by the fact that in many societies, individuals who do not regularly attend school may be the same children who are in the deepest need for supplements due to their poor diet resulting from very low socioeconomic status.

Financial barriers also significantly contribute to the target populations' lack of access to nutritional supplements. Although psychiatric medications are typically subsidized in many countries, this is not the case for nutritional supplements (Darling et al. 2019). As a result, the cost of nutritional supplements is one of the greatest barriers to longer‐term, consistent micronutrient use. In Portugal, 53% of interviewed pharmacists cited the high cost of oral nutritional supplements as the main barrier to access (Gregório et al. 2023).

Of note is that almost all studies on micronutrient supplementation included in this review excluded participants who were taking psychiatric medications at the time of the interventions (see study‐level details on that in the Supporting Information S1). One of the reasons for this is that – according to anecdotal evidence – effective micronutrient supplements (especially broad‐range ones) seem to enhance the effect of psychiatric medications (potentiation) leading to a need for medication dose reduction. However, prescribing clinicians typically do not decrease the medication dosage when this happens due to lack of knowledge regarding medication‐supplement interactions or fear of relapse. Excluding people on medication however proves to be a significant barrier to both recruitment for research purposes as well as general use of supplements among people taking psychiatric medications. It is also worth mentioning though that in comparison to the few studies where medicated participants were actually included, trials that excluded participants using psychotropic medications tended to show a larger beneficial effect (Gast et al. 2023). At this point, it is unclear if that is because psychotropic medications interfere with the beneficial effects of nutritional supplements [cf. in a study of psychiatric inpatients, those individuals not on antipsychotics experienced larger reduction in aggression than those on antipsychotics; (de Bles et al. 2022)] or this phenomenon is related to selection bias: that is, in people with more severe mental health challenges (who more likely take antipsychotics), nutritional supplements are less effective (regardless of whether they take antipsychotics or not).

###### 5.4.2.2.2 Implementation Facilitators

To increase the accessibility of nutritional supplements, some provisioning and innovative distribution approaches are potential facilitators to the logistical and economic challenges. The free provision of iron folate supplements to a population of adolescent girls was shown to facilitate access to nutrition services (Handiso 2023). The use of supplements from produce readily available in the given region is another potential solution to the unavailability of supplements (Lentjes et al. 2014).

Having distribution sites, where supplements are handled directly by a professional, is known to facilitate access and increase their usage. In distribution sites that tested the universal provisioning of free vitamins and where information and training for health professionals were offered, parents were more likely to use vitamins (Jessiman et al. 2013). Health professionals were also more likely to support and advise parents in using vitamin supplements at these locations. Additionally, door‐to‐door distribution has been shown to be more effective than the fixed‐site plus outreach approach, which required caregivers to be aware of the supplementation program and to attend outreach sites with their children (Janmohamed et al. 2017). Door‐to‐door distribution also alleviates the issues of inconvenience to reach a site due to distance and lack of knowledge of their location. Reducing administrative burden for schools, which sometimes serve as distribution sites, can also improve compliance with an intervention (Bommer et al. 2020).

Collaboration across sectors to expand supplementation programs beyond schools and recruit trained experts to reach more people in the target population, regardless of socioeconomic status, can help overcome the socioeconomic barriers to access (Handiso 2023). Addressing constraints in financial resources is key to ensuring that nutritional supplementation programs effectively promote child health and development. Recognizing that cost is a significant barrier to continuing micronutrient treatment, the public healthcare system and private insurers may consider improving access by providing government‐funded or extended‐health‐insurance‐based coverage for the costs of supplements (Darling et al. 2019; Rucklidge et al. 2021). With the contribution of private health insurers, supplement costs could be on par with medication costs to families. To enhance government engagement, it may be important to convey the information that nutritional supplements cost far less than standard mental health treatments (especially if they can prevent hospitalization), as demonstrated through some Canadian case studies (Kaplan et al. 2017; Rodway et al. 2012).

A potential solution to prescribers' hesitancy to reduce psychiatric medication dosages taken parallel to nutritional supplementation is to start treatment with nutrients and add medications only thereafter if necessary. Even in this case, prescribers may want to begin with medication doses lower than what would be prescribed without nutritional supplementation to prevent adverse outcomes resulting from the potentiating effects of nutritional supplements. Psychiatric medication prescribers should seek and be offered reliable and in‐depth information on supplement‐medication interactions to provide them with the necessary knowledge base to serve their clients best. Some supplement producers offer such trainings (e.g., https://try.hardynutritionals.com/syllabus/) or written materials to prescribers (Hardy Nutritionals 2019).^8^

##### 5.4.2.3 Intervention Characteristics

###### 5.4.2.3.1 Implementation Barriers

The complexity of the evidence on the associations between nutritional factors and aggressive/antisocial behavior (Choy 2023) renders decisions for which nutrients to supplement challenging. An additional issue in nutritional supplementation concerns dosage, which can significantly influence effectiveness (a comparison of targeted nutrients and dosages can be found in the Appendix of the work of Gast et al. 2023). While “Daily Reference Values” (DRV) or “Daily Recommended Intake (DRI) Values” exist for the most important nutrients, these values were established with an eye on the prevention of severe somatic diseases (scurvy, iron‐deficiency anemia, osteoporosis, etc.), not on the optimization of mental health. Consequently, supplementation considering these thresholds may not be sufficient, and accordingly, many supplements used in the field of nutritional mental health contain much higher doses than suggested by the Daily Reference or Daily Recommended Intake values. Given that individuals' idiosyncratic nutrient needs may differ significantly, it is not easy to establish universally effective but still safe absolute and relative (balance of different nutrients with each other) dosages for supplementation. The relative imbalance of certain nutrients as a result of narrow‐range supplementation (e.g., omega‐3 vs. omega‐6 fatty acid ratio in relation to omega‐3 fatty acid supplementation) might be a factor in the background of below‐expected efficacy or even treatment harm (cf. data on supplementation with amino acids in Section 5.3.1.3; amino acid supplementation is now not recommended due to risks from toxic catabolic products resulting from increased neurotransmitter turnover [Hurt and Arnold 2014]).

A related issue is the duration of the intervention: that is, how long supplements should be taken to reach some or their maximum effect. While most studies in this field use an intervention duration of 3 months (Qamar et al. 2023), some studies indicate that additional, new improvements in mental health outcomes occur even after 12–15 months of supplementation (Mehl‐Madrona and Mainguy 2017). Of the data collected from our included studies, three studies noted that limitations were low dosages and the short duration of supplementation. In one of these studies, vitamin D (2000 IU/d) supplementation for 8 weeks did not correct vitamin D deficiency in about 30% of participants (Mohammadpour et al. 2018). On the other hand, the quantitative results of the present review regarding omega‐3 fatty acid supplementation to reduce antisocial behaviors indicated that shorter intervention duration was more effective than longer supplementation (see Section 5.3.2.2). Further research, therefore, is clearly needed to clarify the optimal duration and dosage of supplementation in different populations.

Additional insights from our authors revealed that country‐specific regulatory issues might also influence the use and especially the study of nutritional supplements, leading to suboptimal doses used in interventions. In some countries, supplements containing nutrients in doses above the daily recommended value need to undergo more rigorous approval processes than general food supplements. Their producers, however, may not have the intent or the financial means to do so; therefore, using higher‐dose supplements would be illegal in such contexts, leading to the use of suboptimally low dosages.

###### 5.4.2.3.2 Implementation Facilitators

We propose that continued research efforts in nutritional mental health sciences are needed to build an evidence base, which could inform the public, clinicians, and other stakeholders about the optimal nutritional targets, doses, and treatment durations in the case of various mental health concerns or behavioral problems, including aggression. Psychiatric medication prescribers should be provided with essential training and education on the benefits and management of nutritional interventions in general and supplements specifically, through the available guides for medication prescribers on supplementation or online training modules.

Of the data collected from our included studies, one study reported that the number of pills required per day was a concern for some families with younger children; splitting them up to 2–3 times/day made it manageable for most participants (Adams et al. 2018). Even though most of the vitamins were well‐absorbed, for some vitamins, it was beneficial to use larger doses and/or more bioavailable forms, to exert a significant effect on blood levels; larger doses/increased absorption may result in a greater therapeutic benefit (Adams et al. 2018).

##### 5.4.2.4 User Compliance

###### 5.4.2.4.1 Implementation Barriers

There are numerous practical challenges influencing compliance with nutritional supplementation. One concerns the inconvenience of managing the time to visit healthcare settings/pharmacies to purchase the product. An insight from the authors of one study included in the present review revealed that as capsules were taken three times a day, some schools had trouble allowing students to take these without a doctor's note, posing a barrier to taking multi‐nutrients.

Furthermore, a lack of social support also affects compliance. Among South African women, lack of family support, including discouragement from key family members acted as a barrier to nutritional supplementation adherence (Silubonde et al. 2022). Iranian students also listed influence from family and friends as reasons for non‐compliance with nutritional supplementation (Khammarnia et al. 2016).

Individual‐level factors can also serve as barriers to compliance. Forgetfulness has been identified as a challenge to complying with nutritional supplementation (Food Standards Agency 2018; Schultink et al. 1993). An original author we contacted for their expert opinion further noted that forgetting remained an issue despite families receiving regular reminders. This is tied to the issue that the large number of daily capsules was one of the leading factors behind poor adherence and discontinuation of micronutrient supplementation in a randomized trial of children with ADHD (Darling et al. 2019). Longer intervention durations can also pose a greater risk for non‐compliance (Chang et al. 2019).

The way nutritional supplements are administered can also affect compliance. In the United Kingdom, participants who took gels and sprays found it challenging to ensure that they were taking the recommended dosage (Food Standards Agency 2018). Furthermore, the large size of multivitamin and mineral capsules proved to be a challenge for swallowability (de Bles et al. 2022), especially for younger children. Similarly, a cross‐sectional study on adherence to oral nutrition supplements in Sweden cited swallowing difficulties, as well as taste and texture, as reasons why consuming supplements was sometimes challenging (Liljeberg et al. 2019).

In the current review, the overall drop‐out rate from nutritional supplementation in the included studies was 10.55% on average (with a standard deviation of 10.44%). In one of the studies, five families did not participate due to objections against pork‐gelatin‐containing capsule casings (Widenhorn‐Müller et al. 2014). Another study reported that approximately 20% of the participants had problems with the taste of the fish oil and/or the unflavored vitamin/mineral supplement (Adams et al. 2018). Additional insights from authors of our included studies also included unfavorable sensory characteristics of food supplements with high omega‐3 content (e.g., smell). Another author reflected on the link between low compliance and conduct problems, where non‐adherence with expectations is a defining feature of the disorder itself (cf. oppositional defiant disorder, antisocial personality disorder).

Although side effects are typically minor and transient (Rucklidge et al. 2023) and they differ from supplement to supplement (depending on nutrient, dosage, and supplementation format), their effect on compliance is also important to consider. Regarding omega‐3 fatty acid supplementation, most of the original studies included in the quantitative section of this review reported no adverse events or a side effect profile not significantly different from that of the placebo group (Crippa et al. 2019; Dean et al. 2014; Gustafsson et al. 2010; Johnson et al. 2010; Mankad et al. 2015; Richardson and Montgomery 2005; Widenhorn‐Müller et al. 2014). Those authors who did observe intervention‐specific side effects typically reported mild gastrointestinal symptoms or rarely nose bleeding (Manor et al. 2012; Milte et al. 2013; Sinn and Bryan 2007; Young et al. 2017). Similar trends emerged regarding broad‐range‐nutrient and vitamin D supplementation: most studies did not find evidence for intervention‐specific side effects (Gast et al. 2023; Gesch et al. 2002; Hemamy et al. 2021; Johnstone et al. 2022; Mohammadpour et al. 2018; Rucklidge et al. 2018), and investigators of those studies that did, primarily reported mild gastrointestinal symptoms (Elshorbagy et al. 2018; Raine et al. 2016). While these data overall suggest that the side effect burden of nutritional supplements is minor, occasionally they may act as a barrier towards the successful implementation of the intervention. For instance, authors of the only study on zinc supplementation reported that of the total number of individuals who dropped out of the study (12.5%), half did so due to gastrointestinal discomfort caused by zinc sulfate (Arnold et al. 2011).

Healthcare providers' lack of knowledge is also a known factor to influence overall intervention compliance. Pharmacists in Portugal, who considered themselves the main facilitators for patient follow‐up, believed that their lack of knowledge about the characteristics of nutritional supplements for children hindered counseling opportunities and acted as a barrier to adherence (Gregório et al. 2023).

###### 5.4.2.4.2 Implementation Facilitators

Health and nutrition professionals can utilize appropriate communication networks to convey nutrition information and health promotion to overcome the challenges of the misconceptions surrounding nutritional supplements (Zoellner et al. 2009). For example, combining the administration of oral nutrition supplements with dietary counseling is recommended and may increase adherence to these supplements (Liljeberg et al. 2019; Stratton and Elia 2010). To address concerns regarding the misconceptions among parents, increasing their understanding of nutritional supplements and their children's health by providing proper education and support may help reduce the impact of misguided beliefs on treatment compliance (López‐Flores et al. 2012). Addressing the concern that many people are not able to receive an adequate amount of nutrition from their diet alone, the reasons behind this fact, as well as sharing information on the health benefits of supplements, are a few suggestions for improving compliance (van der Wurff et al. 2017). It has been shown that inviting a healthcare professional to provide information and using audiovisual materials can increase students' engagement with nutritional supplements (Jafari et al. 2023).

Incorporating incentives into intervention design is one approach that may be used to address concerns with compliance in research studies (Bommer et al. 2020). One study showed that providing study participants with non‐monetary incentives such as cinema vouchers improved their response rates and increased their motivation to participate in studies (van der Wurff et al. 2017).

Nowadays, most supplements are readily available for purchase online, which may reduce the inconvenience related to the purchase/pick up of supplements. A study investigating the compliance of supplementation showed a higher compliance rate in studies conducted in community settings versus hospital settings (80.9% vs. 67.2%) (Hubbard et al. 2012). The use of school settings compared to hospital settings for interventions also showed a somewhat higher compliance rate (86% vs. 82.9%) (van der Wurff et al. 2017). This notion was supported by an author of one of our included studies, who indicated that the school setting (in the United States) seemed to result in excellent compliance and follow‐up rates (Schoenthaler and Bier 2000).

Support from treatment providers and the community can also facilitate user compliance. Higher compliance with nutritional supplementation has been found among children of indigenous origin in a semi‐rural community, most likely because the supplement distributors in the study were from within the community and were able to better address any concerns and hesitations among community members (López‐Flores et al. 2012). There is also evidence of a positive association between motivation and higher treatment compliance, where the healthcare providers used a more empathetic and affective approach to treatment, addressing the needs and concerns of the client (López‐Flores et al. 2012). Other studies have highlighted that adherence to supplementation was higher in children and adolescents who were supported by family and friends (van der Wurff et al. 2017). An author from our included studies suggested that many of their study participants had previously been patients at their institute, which acted as a significant facilitator due to the trust developed over the course of treatment. Thus, taking supplementation within the context of an existing collaborative or therapeutic relationship—such as one with a family physician, midwife, nurse practitioner, psychologist, or teacher—may enhance compliance.

Several studies also highlight that regularly communicating with parents and participants of research studies via phone calls, emails, or in‐person was important in increasing adherence (de Bles et al. 2022), which can be particularly important in addressing concerns about forgetfulness. In a study, between each visit, parents were contacted through email or phone calls at least 3 times by the researchers to reduce dropouts and informally check compliance (Milte et al. 2013). An additional potential facilitator for improving compliance rates is giving consumers feedback on the effect of their compliance based on biochemical analyses (e.g., blood or urine analysis) or mental health outcome data (e.g., if users regularly report their symptoms in a phone app/online portal, which also provides feedback on their health outcomes).

To increase convenience, keeping the supplementation regime simple and easy‐to‐follow is also an important facilitator. This may involve, for example, the use of one concentrated capsule instead of many throughout the day (van der Wurff et al. 2017). Potential strategies such as postural adjustment, the use of pill‐swallowing aids, and teaching pill‐swallowing techniques help improve compliance in clients with pill‐swallowing difficulties (de Bles et al. 2022; Kaplan et al. 2010). Several authors of our eligible studies specifically mentioned an easy‐to‐access (YouTube) training video from Professor Bonnie Kaplan (https://youtu.be/Zxqs7flHJQc) as a key and effective aid with pill‐swallowing difficulties. Authors of some studies reported that they had clinicians on their team who used a pill‐swallowing protocol to help children learn how to swallow pills.

The method of administration of nutritional supplements should also be considered. Alternative approaches, such as the modification of pills by crushing them and mixing them in foods or drinks, can also help with swallowing (de Bles et al. 2022). Particularly for younger children who cannot describe their symptoms, mothers have reported that when using drops to administer iron, 74% rejected the supplement (crying, spit drops, gestures), but this percentage went to only 16% when administered as a powder mixed with food (Galloway and McGuire 1994). Authors of a Japanese study on school children's aggression argued for fortification as a helpful way of increasing compliance: fish oil was added to bread, sausage, and spaghetti, which seemingly led to excellent compliance among the 9–12‐year‐old children (Itomura et al. 2005).

Some authors suggested the use of soft gel capsules to deliver the supplements as they proved to be easier to swallow, and this format also helped mask unpleasant tastes and odors (Benza and Munyendo 2011). An additional author insight revealed that to overcome the challenges of swallowing the fatty acid capsules in some participants, squeezing the oil out from the capsule directly into the mouth was a potential solution that worked effectively. Cultural, religious, or personal preferences also need to be considered; vegan versions of supplements are often available and thus should be offered to increase compliance (e.g., algae instead of fish‐oil‐based omega‐3 fatty acid supplements; capsule materials not containing animal ingredients).

If capsules still prove to be a challenge, other options such as chewable tablets with an appealing taste could be used to administer the supplementation (Benza and Munyendo 2011). Some producers offer various other child‐friendly forms of supplementation (e.g., lollipop stick for broad‐range micronutrients, “EMPowerplus Ultimate Sticks”^9^). Of the data collected from our included studies, two studies used a fruit‐flavored drink to supplement with omega‐3 fatty acids. The investigators argued that such products contain high enough doses of omega‐3 fatty acids in a relatively small amount of liquid, and they are better tolerated due to their fruit flavor, resulting in higher compliance in children than standard capsules (on average 6.1 drinks per week out of 7 offered) (Raine et al. 2016; Raine et al. 2020). In a study from Sweden, participants were given a choice of flavors for their nutritional supplement; therefore, many participants actually liked the taste of their supplement, which likely contributed to the high adherence rate (Liljeberg et al. 2019). One study from Ghana investigated the use of sprinkles as a means of supplementation; when added to the food, these do not alter taste or color (Zlotkin et al. 2001). Furthermore, when they are manufactured in single‐dose sachets, they reduce the likelihood of an accidental overdose from too much ingestion because each sachet would have to be opened separately to do so. Another advantage to using sprinkles over capsules or drops is that multiple micronutrients can be included in the sachet depending on the needs of specific individuals (Zlotkin et al. 2001).

While some studies have found that side effect burden can negatively impact compliance to multi‐nutrient supplements, some authors noted that participants who used supplements long‐term experienced symptom improvement, leading to improved compliance (Rucklidge et al. 2021; Silubonde et al. 2022). This finding highlights the importance of communicating the fact that it takes time for benefits to manifest to individuals receiving multi‐nutrient treatment (Rucklidge et al. 2021). With regard to supplement formulations, administering a controlled‐release format of certain supplements may decrease supplement side effects (Galloway and McGuire 1994). To reduce potential side effects, the authors of one included study reported that taking omega‐3 fatty acid supplements at bedtime or with meals reduced burping and improved adherence. Of the data collected from our included studies, one study noted that choosing lunchtime for broad‐range supplementation minimized possible side effects such as nausea and belching (Zaalberg et al. 2010). Another study determined that using zinc glycinate instead of zinc sulfate reduced side effects such as gastrointestinal discomfort (Arnold et al. 2011). These findings suggest that often there are supplement‐specific ways of reducing the chances of side effects, which are important to consider when planning a supplementation regimen.

##### 5.4.2.5 Intervention‐Interfering Processes

###### 5.4.2.5.1 Implementation Barriers

Illnesses that affect body weight or gut health may influence the effectiveness of treatment with nutritional supplements. For example, individuals with inflammatory bowel disease or Crohn's disease are shown to have deficiencies in a wide range of micronutrients, also due to malabsorption (Berger et al. 2019). Certain medications or conditions can also cause micronutrient deficiencies due to their interference with absorption or excretion. For instance, certain antidepressants influence gut motility or the gut microbiome, leading to changes in nutrient absorption, while stimulant ADHD medications increase the excretion of certain minerals. Further, some antibiotics can cause deficiency in magnesium and various B vitamins, while certain drugs containing sulfhydryl can cause zinc excretion disturbances (D'Alessandro et al. 2022). Antipsychotic medications can also cause depletion in vitamin B12, and deficiencies in vitamin D can be caused by antacids and certain anticonvulsants (D'Alessandro et al. 2022). Taking such medications in combination with a nutritional supplement may negatively influence the effectiveness of the nutritional intervention. Moreover, children with ADHD may have a zinc‐wasting metabolism such that zinc administered in the morning is excreted soon, but while in the system, the supplemented zinc may (temporarily) improve ADHD symptoms (acting as an immediate‐release stimulant) (Arnold et al. 2011).

Heterogeneity in response to omega‐3 supplementation has also been linked to genetic factors. A specific gene cluster (FADS) is shown, for example, to significantly impact omega‐3 biosynthesis and metabolism, and it has been proposed to be a major factor behind the heterogeneity in the results of clinical trials on omega‐3 supplementation and heart disease (Fernandez et al. 2021). It is possible, therefore, that specific genetic polymorphisms significantly influence the effectiveness of nutritional supplements more broadly.

The interaction of nutritional factors also warrants consideration. For example, in a study on omega‐3 fatty acid supplementation in ADHD, the authors noted that the arachidonic acid (an omega‐6 fatty acid) levels of the children were also low, interfering with beneficial treatment outcomes requiring an optimal balance of the two groups of fatty acids (Voigt et al. 2001). Therefore, aiming for an optimal ratio of supplemented nutrients should be a crucial consideration when planning interventions.

###### 5.4.2.5.2 Implementation Facilitators

For interventions using fish oil, it is important to ensure that it is refined to eliminate contaminants such as mercury, since heavy metals also tend to increase aggression (Yıldız et al. 2023). Combining DHA (an omega‐3 fatty acid) supplementation with arachidonic acid (an omega‐6 fatty acid) supplementation can account for the reduced level of arachidonic acid in certain populations (Voigt et al. 2001). The absorption of several nutrients requires the digestion of fat; consequently, taking supplements containing such nutrients is better in conjunction with meals of adequate fat content.

Developmental factors (pubertal development) and physical activity level should also be considered when managing nutritional disturbances in adolescents, especially with eating disorders (Nicholls and Viner 2005). Ongoing monitoring by a dietitian is a helpful part of any nutritional intervention, including supplementation, which should be obligatory in certain physical health conditions. Dietitians can help supplement users select the most appropriate nutrient amounts and balance nutrient and fluid intake, as well as counsel regarding food‐related behaviors and attitudes. Furthermore, it is helpful if dietitians and physicians are aware of the possible food‐drug interactions that could occur and should consider using adequate supplements and dosages when combining supplementation with certain pharmacological treatments.

## 6 Discussion

### 6.1 Summary of the Main Results

We identified a relatively large number of studies that investigated the effectiveness of nutritional interventions in reducing aggression, antisocial behaviors, or criminal offending in children/youth. However, the heterogeneity of this literature in many regards (e.g., target populations, specifics of nutritional intervention) makes it difficult to draw clear and firm conclusions. Nevertheless, it seems that nutritional interventions with a broad nutritional target have the most consistent and largest intervention benefit across all outcomes (aggression: *g* = −0.31, *p* = 0.001; antisocial behavior: *g* = −0.49, *p* < 0.001; criminal offending: *g* = −1.25, *p* = 0.031). These effect sizes range from small (aggression) to medium (antisocial behavior) to large (offending). Another consistent trend across outcomes (aggression, antisocial behavior, and criminal offending) was that dietary change seemed to be remarkably more effective than nutritional supplementation, even if both affected a broad range of nutrients (e.g., complete diet change vs. broad‐spectrum micronutrient supplementation). The difference between effect sizes was significant both in terms of absolute numeric value (e.g., Δ*g* = 1.17 for criminal offending) and categorization of the effect size (small for supplementation, while large for diet change in the case of antisocial behavior; small for supplementation but moderate for diet change in the case of aggression). Another moderator that emerged as significant regarding interventions with a broad nutritional target was the proportion of males: both for aggression (Δ*g* = 0.35) and antisocial behaviors (Δ*g* = 0.63), studies with a larger proportion of males showed superior effect sizes than those with fewer males in the sample. This may be related to both selection bias (e.g., male‐only samples have higher baseline aggression levels) or true differences in the efficacy of nutritional interventions across the sexes.

The most studied narrow‐focused nutritional intervention was omega‐3 fatty acid supplementation, with a small effect size and with less consistency in statistical reliability (aggression: *g* = −0.33, *p* = 0.240; antisocial behavior: *g* = −0.15, *p* = 0.013) than what we observed for broad‐focused nutritional interventions. The other narrow‐focused nutritional intervention that was studied enough to draw at least preliminary conclusions was vitamin D supplementation. There was a small‐to‐moderate treatment benefit in terms of antisocial behaviors across the four studies investigating it (*g* = −0.48, *p* < 0.001). While there were several other studies on narrow‐focused nutritional interventions, these were not studied frequently enough to allow the generation of even preliminary meta‐analytic conclusions.

Of note, prediction intervals – which take into account heterogeneity to provide a range within which we expect the true effect size of a future study to fall – always include the possibility of no effect. This confirms that heterogeneity in the data is considerable, that is, results may not be consistent across different contexts. This suggests that in future research/implementation efforts, nutritional interventions in certain populations will not be effective in reducing aggression, antisocial behaviors, or offending. Exploring the sources of this heterogeneity (e.g., dosages in the case of supplementation) is an important task for future research in nutritional (mental) health sciences.

### 6.2 Overall Completeness and Applicability of Evidence

The literature characterized in this review is quite diverse in many regards. For instance, it includes study populations from many countries (from different regions of the world), ranging from Japan to the United States and from Iran to New Zealand. However, as mentioned above, the vast heterogeneity in intervention characteristics and study populations means that several types of interventions or populations were only or mainly studied in a narrow context or by very few studies. For instance, all four studies on vitamin D were conducted in the Middle East (Egypt and Iran), where sun exposure (and so vitamin D deficiency) is most likely less of a challenge than in countries with less sunny hours (Cui et al. 2023). Similarly, both studies with offending as the outcome were conducted in the United States, where the general population has a generally less healthy diet than, for example, in Middle Eastern countries (Wang et al. 2020).

In addition, the original studies synthesized here are heavily focused on younger children with neurodevelopmental disorders (e.g., ADHD) and in the community setting; and consequently, much less research attention has been devoted to adolescents or young adults without a neurodevelopmental disorder diagnosis (which may or may not be an indication of a true absence of such a disorder), living in residential settings or who have been in contact with the criminal justice system.

Further, in the vast majority of cases, we extracted and considered intention‐to‐treat efficacy data,^10^ which is a conservative approach. This raises the possibility that treatment effects for individuals who actually adhere to a nutritional intervention may be somewhat larger than suggested by the results of the present review.

Given the vast heterogeneity in the potential target populations, intervention characteristics, and in the statistical results of some of our meta‐analyses, the results of this review should by no means be considered as complete or definitive. Instead, they should be seen as one of the preliminary attempts to characterize a new research– and clinical field (i.e., nutritional‐behavioral sciences) still in its infancy.

### 6.3 Quality of the Evidence

Beyond the above considerations regarding the heterogeneity and the resulting limitations regarding generalizability, the overall number, sample size, and risk of bias of the included studies also limit our confidence in the validity of certain results of this meta‐analysis.

While broad‐spectrum nutritional interventions were studied in a reasonably large number of studies with a considerable overall sample size regarding aggression (7 studies, total *N* = 797) and antisocial behaviors (13 studies, total *N* = 2109), only two studies were conducted in relation to offending (total *N* = 117), significantly limiting the generalizability of the findings in relation to this latest outcome. The fact that both studies on offending were published a long time (28 and 47 years) ago may also reduce our confidence somewhat that their results are valid for the current state of affairs (cf. change in nutrient content of natural food resources over time, potential shifts in general dietary habits, and institutional dietary policies).

Further, risk of bias and its effect on the results was considerable for broad‐spectrum nutritional interventions: eliminating studies with high risk of bias reduced treatment benefit to close to zero regarding aggression (after eliminating 71% of studies) and from moderate to small (but still statistically significant) in relation to antisocial behaviors (after eliminating 23% of studies). It is also worth mentioning, though, that studying dietary manipulation's effect on aggression is extremely difficult without a high risk of bias as per the assessment algorithm of the Cochrane Risk of Bias Tool (RoB 2). The RoB 2 assigns high risk of bias to any study where the outcome assessors are aware of the intervention (Higgins et al. 2019), which almost automatically results in high risk of bias regarding any study with dietary manipulation. In these studies, the outcome is typically assessed by study participants themselves (self‐reported measures of aggression/antisocial behaviors) or family members (who both “deliver the intervention” and evaluate the outcome). Even if the outcome is rated by others than the participants or family members (e.g., prison officers), chances are high that those who are close enough to observe and assess the participant's behavior are also close enough to notice changes in dietary patterns. Therefore, this literature most likely will always be characterized by a high risk of bias (with the exception of some specific contexts, such as probation, where re‐offending statistics can be obtained without the knowledge of an individual's dietary patterns). While a lower risk of bias is attainable in studies that investigate broad‐range *supplementation* (where the blinding of both participants and evaluators is more achievable with a placebo), from the implementation perspective, these are qualitatively different from studies introducing changes in *dietary patterns*.

Omega‐3 fatty acid supplementation was studied in nine studies (total *N* = 706) in relation to aggression as the outcome, and in 21 studies (total *N* = 2081) in relation to antisocial behavior as the outcome. While in the former case, eliminating indicators with a high risk of bias did not change the originally small effect size, this procedure further reduced the negligibly small effect size regarding antisocial behavior.

Finally, vitamin D supplementation's effect on antisocial behaviors was studied in four studies only (total *N* = 226), and the elimination of studies with high risk of bias reduced the pooled effect size from moderate to small (but still statistically significant).

The above patterns of the results are in line with the infant stage of this field (cf. numerous small and low‐quality studies) and calls attention to the preliminary nature of our findings and the conclusions to be drawn from them.

### 6.4 Potential Biases in the Review Process

Strengths of this review include the strong adherence to a publicly available a priori protocol (Konkolÿ Thege et al. 2024), involvement of an expert librarian to develop the search algorithm, and the use of two independent coders in all major stages of data collection (initial screening, eligibility checking, data extraction, and risk of bias assessment). Weaknesses include the uncertainty with identifying all relevant studies, given the large variability in whether or how authors name the constructs relevant for our purposes. For instance, conduct problems are often measured by subscales of more complex assessment tools, and authors may or may not refer to those subscales or subconstructs in their work if they are not interested in behavioral problems. This raises the possibility that we have missed studies on relevant outcomes and populations despite our dedicated effort to make the searches as comprehensive as possible.

Another limitation is that, due to resource constraints, we decided to extract one indicator per outcome, which is less ideal than extracting and analyzing all indicators via multi‐level modeling (Tipton et al. 2023). We identified numerous studies where multiple indicators were reported for the same outcome for the same study group. Even though we followed a transparent, protocol‐based algorithm to decide which indicator to extract and consider in the quantitative analyses, it is possible that the principles guiding the algorithm introduced bias. For instance, we prioritized observer over self‐reported indicators, although it is possible that individuals know and are willing to share more about their behavior than observers. This may be especially true outside the correctional/justice system and in older children/young adults whose social life often includes spheres not overseen by parents or teachers. In line with our protocol, however, we collected information on the indicators we did not consider and made this available in the Supporting Information S1, thus facilitating the consideration of these alternative indicators by researchers with more resources.

We also decided to focus on populations with elevated levels of aggression/antisocial behavior to avoid any floor effect, and for this, we considered both direct (e.g., elevated scores on a scale measuring aggression) and indirect (e.g., mental health diagnosis often co‐occurring with aggressive behaviors) indicators. It is unclear though how reliable our indirect indicators were; it is possible that some of the participants did not present with elevated level of aggression/antisocial behavior despite their ADHD diagnosis, for example, which – if occurred – could incorrectly lead to effect sizes lower than might be observed when restricting analyses to only those participants indeed exhibiting elevated aggression/antisocial behavior. In addition, there are several studies investigating the effectiveness of nutritional interventions to reduce aggression in the general population (with no indication of elevated levels of aggression/antisociality), indicating benefits even there. For instance, a French study of 195 adults from the general population observed a significantly larger reduction in aggression in the group consuming omega‐3 supplements than in the placebo group (Bègue et al. 2018). A Swedish trial of infants with low birth weight indicated that babies who received iron supplements from 6 weeks to 6 months of age showed less aggressive and rule‐breaking behaviors at 7 years of age than members of the placebo group (Berglund et al. 2018). A study on school children living in Mauritius also showed a statistically significant (and moderately strong) treatment benefit from omega‐3 fatty acid supplementation in terms of reduced levels of aggression (Raine et al. 2015). However, there is a larger group of studies conducted in the general population showing no intervention benefit (see list of these in the Supporting Information S1 among the excluded studies with a label “No excess aggression in sample”) at least partially confirming the validity of our original concern regarding floor effects.

Given the large heterogeneity in interventions (e.g., from vitamin D supplementation to complete diet change) and study populations (e.g., from preschool‐age children with ADHD to young adults with criminal offenses) as well as the multiple outcomes, we grouped studies together to be able to form at least preliminary judgements on the effectiveness of nutritional interventions in reducing aggression, antisocial behaviors and offending. Such groupings (e.g., broad‐range nutrient supplementation vs. general improvement in diet quality; prisoners vs. individuals living with autism in a residential setting; or preschool‐aged children vs. young adults) may not be optimal, can be debated, and should be avoided if, with time, more and more homogenous data become available. While we ran subgroup analyses to investigate the moderator effect of such grouping variables, the number of studies per subgroup was typically so low that the reliability of the resulting non‐significant effect of the grouping variables is highly questionable. Further, subgroup analyses are observational in nature; where subgroup analyses did detect significant differences, this could be due to other variables beyond those we chose to investigate.

In addition, and in accordance with the infancy stage of this particular field, many of the included studies' reporting standards were poor. This required us to use eight different types of data formats (e.g., absolute values at intervention‐end only, absolute values at both baseline and intervention‐end, change scores and their standard deviation, *F*‐value, event counts, etc.) through which we calculated our one single effect measure of Hedges' *g*. While this approach allowed us to pool more studies into a single analysis to provide better‐powered conclusions, this tactic is debatable and is sometimes considered suboptimal (e.g., collapsing absolute vs. change scores). Using absolute values at follow‐up can introduce spurious variation if groups are non‐equivalent at baseline; comparing aggregate absolute values at follow‐up with aggregate absolute values at baseline is also not a straightforward proxy of mean change without knowing the correlation between baseline and follow‐up values (Higgins et al. 2019), which were not available for our outcomes of interest.

Most high‐quality meta‐analyses consider original studies only if there is a control group in parallel to the intervention group, as such designs provide better chances to increase internal validity, and we followed this selection principle as well. However, other powerful designs do exist, which, even though they do not employ a control group, are still internally more valid than simple pre–post studies. For instance, a US study on the antisocial behavior of young people who had offended used an ABA design: the initial baseline phase was followed by the intervention phase (reduced‐sugar diet), after which participants returned to their baseline diet. While in the second phase the investigators observed a 45% decrease in antisocial behavior, it increased by 54% in the final phase of the study, making it much more likely that the observed positive changes during the intervention phase were indeed the result of the intervention (Logan and Schoenthaler 2023). With the even more complex ABAB design, even case series could be quite informative. In such a study, for instance, pre‐adolescent boys with mood lability and explosive rage were treated with a broad‐range micronutrient supplement. The boys' mood, angry outbursts, and other mental health symptoms improved when initially treated, returned when not taking the supplement, and again improved (and were sustained over 2 years) when the micronutrient supplement was reintroduced (Kaplan et al. 2002). Consideration of too many different study designs – including ones typically considered as lower quality – in a meta‐analysis may not be feasible. However, overreliance on controlled and especially randomized controlled studies may lead to inefficient use of our existing data and resources as well as ignorance of clinically relevant, alternative sources of reliable information (Kaplan et al. 2011).

Finally, although the present author team dedicated significant efforts to the identification of the most relevant information on implementation‐related barriers and facilitators, this section of the present report is not as comprehensive and reproducible as the main, quantitatively focused component. Instead, the implementation‐related section is to be seen as a narrative review reflecting the authors' current – most likely biased and incomplete – understanding of this field, even if we followed all information threads provided by the eligible studies of the systematic literature search (focused on efficacy).

### 6.5 Agreements and Disagreements With Other Reviews

There is a limited number of systematic reviews that present results that can be contrasted with and to the best of our knowledge, none of them applied the same eligibility criteria as ours, that is, they did not focus on children and young adults only, or did not focus on populations with above‐normal aggression levels. Further, all meta‐analyses to date have focused on the effect of omega‐3 supplementation on aggression/antisocial behaviors. No other nutritional intervention was considered for meta‐analytic purposes regarding these outcomes.

The first meta‐analytic finding was published in 2007 based on much less available evidence at the time and with a much bigger emphasis on qualitative elaboration on underlying mechanisms of action than actual meta‐analytic calculations. Such calculations, though, showed bigger effect sizes (standardized mean difference of −0.61) in relation to the effectiveness of omega‐3 fatty acid supplementation in reducing antisocial behaviors across all age groups (Benton 2007) than observed in the present study, where the standardized mean difference was −0.33 for aggression and −0.15 for antisocial behaviors. Authors of a more recent meta‐analysis (Gajos and Beaver 2016) focusing on aggression (including externalizing and oppositional behaviors) reported an effect size more similar to the one observed in our study. The standardized mean difference for all studies with a controlled design (regardless of participant age and specific fatty acid supplement) was −0.24 (95% CI = 0.12–0.36) in this 2016 meta‐analysis. Finally, the latest meta‐analysis (Raine and Brodrick 2024) with a comparable focus (still, not limited to children/youth or populations with above‐normal levels of aggression) reported that omega‐3 fatty acid supplementation reduced aggression with a small but statistically significant magnitude (*g* = −0.16, *p* = 0.006).

Overall, the above studies – with the exception of the first meta‐analysis relying on a much narrower evidence base that was available in 2006 – seem to consistently support the notion that omega‐3 fatty acid supplementation has a small but beneficial effect on reducing aggression/antisocial behaviors. Results of systematic reviews without a meta‐analysis were also in line with the findings of the present study in terms of preliminary support for the efficacy of broad‐range micronutrient supplementation in reducing aggression and antisocial behaviors (Qamar et al. 2023; Rucklidge and Kaplan 2013).

## 7 Authors' Conclusions

### 7.1 Implications for Practice and Policy

In the default analyses of the present review, several interventions (interventions with a broad nutrient focus and vitamin D supplementation) resulted in close‐to‐moderate‐size or moderate‐size treatment benefits regarding one or more of the relevant outcomes. The sensitivity analyses reduced these values substantially to similarly small values than what was observed for omega‐3 supplementation, even in the default set of analyses. When interpreting this pattern of reduced (due to sensitivity analyses) or even originally small effect sizes regarding the efficacy of dietary interventions in reducing aggression/antisocial behaviors, it is important to consider though that an effect size that is irrelevantly small for a clinician or trialist can be very significant from the public health perspective (Carey et al. 2023; Popper et al. 2017). That is, when a relatively simple and widely accessible intervention – such as nutritional interventions – has the potential to influence large segments of the population, then even a small effect can have major and socially relevant positive consequences. It is also possible that the use of aggregate data could mask significant variation between participants – that is, some participants may have remarkable benefit from an intervention, others might not be impacted by the intervention at all, and this could result in a small or moderate pooled effect size, when in reality none of the participants experienced small or moderate effects as individuals.

All in all, we agree with the conclusions of the authors of the most recent meta‐analysis focusing on the benefits of omega‐3 supplementation in terms of aggression reduction (Raine and Brodrick 2024). Together with these authors but extrapolating their point to broad‐spectrum nutritional interventions as well, we argue that while the evidence on such interventions to reduce aggression is not yet conclusive, these interventions are safe (especially compared to psychotropic medications), easy‐to‐implement (especially nutritional supplements), and cheap (especially on the societal level; for the individual, it is dependent on a large variety of variables). These characteristics make such interventions feasible and desirable (Hurt and Arnold 2014), especially as their benefits are very far from being aggression‐specific. Better nutritional status is the basis for both physical and mental health in general (Kaplan and Rucklidge 2021); and therefore, investment in nutritional interventions (either as improvement in overall diet quality or through supplementation with nutrients proven to be necessary for overall nervous system health) seems warranted.

In the case of such a complex phenomenon as aggression/antisocial behavior, manipulation of any single variable will not solve the entire problem, but each element of the complex picture can contribute to the overall solution. With the words of a probation officer arguing for the necessity of nutritional interventions in the correctional context, trying to correct a person's behavior in the absence of essential nutrients is like trying to ride a bicycle without filling the tires with air (Logan and Schoenthaler 2023). In the section on implementation, we reviewed a large number of variables in various contexts that can hinder efforts to improve nutritional status, but solutions to most of these challenges are available, supporting even large‐scale implementation.

### 7.2 Implications for Research

While we identified a relatively large number of studies in this systematic review, we have to acknowledge that research in this field is still in its infancy. Nutritional interventions are quite complex in themselves (e.g., different target nutrients with vastly different dosages), and they interact with numerous moderators (e.g., unknown idiosyncratic metabolic needs, medications, gut health influencing absorption, etc.). Due to a lack of the full understanding of the neurobiology of aggression, we also do not know for sure whether the very heterogeneous study populations (cf. young children with ADHD or autism vs. incarcerated youth) could be rightfully combined, that is, whether the neurobiological aspects of their aggressive/antisocial behaviors are identical, only similar or completely different and so whether it makes sense to investigate the same nutritional interventions in these populations. Further investigation is also needed into differential effects across individuals; for instance, subgroup analyses gave some indication that effect sizes may be more pronounced in males.

Consequently, much more research is needed in more homogenous populations with more homogenous nutritional interventions (both in terms of target nutrient, dosage,^11^ and intervention length) so more definitive conclusions can be drawn on to whom and what type of nutritional interventions are (most) effective in reducing aggressive/antisocial behaviors and offending.

An important future focus of study should be whether different forms of aggression (e.g., reactive‐, proactive‐, or self‐directed aggression) are similar or not in terms of their neurobiological correlates and responsiveness to nutritional interventions. While a few studies have been completed with an eye on these more subtle nuances (Raine et al. 2019; Raine et al. 2016; Raine et al. 2020), their number and conclusiveness are very limited. We also believe that it would be important to better understand *how* nutritional interventions may contribute to the reduction of aggressive/antisocial behaviors. One potential explanation is that better nutritional status reduces irritability and angry feelings (Buydens‐Branchey and Branchey 2008; Mehl‐Madrona et al. 2010), but it is also possible that the major mechanism of action is through improved executive functioning (i.e., capacity to regulate behavioral responses to frustration and anger) (Hansen et al. 2015; Muth and Park 2021; Patrick and Ames 2015).

An important inconsistency in the field, desperately needing further research, is related to the necessity of pre‐intervention nutrition level assessments. While some authors argue that nutritional supplementation should only be given to those displaying a measurable “absolute” deficiency (Schoenthaler et al. 1997), others argue that this is not feasible based on theoretical (Rucklidge et al. 2021) or empirical reasons (Rucklidge et al. 2019). The former group of experts argues that the aggression of participants who were treatment responders was related to suboptimal pre‐intervention nutrient levels. In contrast, proponents of the latter notion point out that due to the idiosyncrasy of individuals' nutritional needs (cf. the influence or stress, specific metabolic or gut microbiota characteristics, etc.), our current, relatively simplistic assessment protocols for nutrient levels are not necessarily helpful in determining who might benefit from nutrient supplementation (Kaplan et al. 2007). Considering the practical importance of this question, future studies should systematically collect information on whether pre‐intervention nutritional assessment is helpful in predicting treatment response and if yes, (1) whether the thresholds to consider are universal or somewhat population‐specific, and (2) whether pre‐intervention absolute nutrient values are sufficient to consider or the ratio of different nutrients compared to each other also needs attention (Stevens et al. 2003).

The literature on the efficacy of nutritional interventions in reducing aggression/antisocial behavior contains a relatively large number of crossover studies. However, our knowledge of exactly how much time it takes for different supplemented nutrients to disappear from the system of participants is limited (Gast et al. 2023). Therefore, the interpretability of data from such studies is often suboptimal; that is, we sometimes do not know if the lack of difference between the active and placebo phase comes from a true lack of intervention effect or the lingering effect of the intervention when the placebo phase follows the active phase. Accordingly, future authors need to be careful when making decisions on the length of the washout period and justify their choices when designing studies with crossover features.

Finally, we also strongly encourage future authors to follow standard reporting guidelines and accordingly, report both descriptive data and detailed statistical results, especially effect sizes, which are still often missing even from recent authors' study descriptions and interpretations of the clinical/practical relevance of their findings. The vast majority of the studies we had the opportunity to assess as part of this review only considered the statistical significance – and in an ideal scenario, the effect size – of the group‐level change in relation to a nutritional intervention. Even the highest‐quality studies paid no to minimal attention to clinical significance (Ogles et al. 2001), which deals with the homogeneity of treatment effects across the intervention recipients (changes on the individual level). It makes a big difference, however, whether an intervention results in a slight change in most participants versus leaves most participants unchanged but causes dramatic improvements in only a few cases. For instance, in a US prison study of young people who had offended and consumed a reduced‐sugar diet for 3 months, the number of serious incidents dropped by 47%. However, this reduction in the overall number of incidents was due to improvements in the behavior of only 16% of the 1382 participants (Schoenthaler 1983c). Systematic consideration and reporting of information on clinical significance in future studies would have high value both from a theoretical and practical perspective.

## Author Contributions

Barna Konkolÿ Thege conceptualized the study. Barna Konkolÿ Thege drafted the protocol with input from Eden A. Kinzel, Olivia Choy, and Jamie Hartmann‐Boyce. Electronic database searches were completed by Eden A. Kinzel, while other searches were conducted by Chaz Robitaille, Lujayn Mahmoud, Rameen Qamar, and Barna Konkolÿ Thege. Initial screening, screening for eligibility, data extraction, and risk of bias assessment were conducted by Chaz Robitaille, Lujayn Mahmoud, and Barna Konkolÿ Thege. Statistical analyses were conducted by Barna Konkolÿ Thege and Jamie Hartmann‐Boyce. The first draft of the manuscript was written by Barna Konkolÿ Thege, Rameen Qamar, and Olivia Choy, and all authors read and approved the final version.

## Conflicts of Interest

Olivia Choy was involved in the publication of two randomized controlled trials of omega‐3/vitamin D supplementation on young people who have offended and children with externalizing behavior disorders. The other authors declare no conflicts of interest.

## Plans for Updating This Review

This review will not be updated by the project team as the end of our funding period is June 2025. Teams interested in building on this review or contributing to updating it are encouraged to contact the corresponding author.

## Peer Review

The peer review history for this article is available at https://www.webofscience.com/api/gateway/wos/peer-review/10.1002/cl2.70059.

## Supporting information


**Supporting Information S1:** 1. Bibliographic details for all included and excluded studies. 2. All data extracted from eligible studies. 3. The exact data set used for the meta‐analyses. 4. Detailed results of the subgroup analyses.

**Supporting Information S2:** Final search strings of the electronic database searches.

## Data Availability

The data that support the findings of this study are available in the Supporting Information of this article.

## Endnotes

1 In one study, the vast majority of participants did not make it to the post‐cross‐over phase, and the age of those who did was above our eligibility threshold (Gast et al. 2023). In the other study, the design was a one‐way crossover, not allowing the comparison of an intervention and a control group at the end of the second stage, as members of both groups received the active intervention (Richardson and Montgomery 2005).

2 Treatment effect in the five studies with high risk of bias was *g* = −0.44 (95% CI = −0.63 to −0.24, *z* = −4.40, *p* < 0.001), which statistically significantly differed (*Q* = 4.93, *p* = 0.026) from the pooled effect of studies with low/moderate risk of bias.

3 Note that traditionally, a p‐value of 0.1 is considered as the threshold for statistical significance in relation to subgroup analysis in meta‐analyses due to the typically – here as well – low sample sizes in the subgroups. Consequently, the *p*‐value of 0.096 is considered as statistically significant. The term “remarkably” is intended to call attention to the magnitude of the effect size difference (Δ*g* = 0.44).

4 Treatment effect for the only outcome with high risk of bias was *g* = −0.30 (95% CI = −0.70 to 0.11, *z* = −1.44, *p* = 0.149), which did not differ statistically significantly (*Q* < 0.01, *p* = 0.930) from the pooled effect of studies with low/moderate risk of bias.

5 Treatment effect in the three studies with high risk of bias was *g* = −0.96 (95% CI = −1.85 to −0.06, *z* = −2.10, *p* = 0.036), which did not differ statistically significantly (*Q* = 1.86, *p* = 0.172) from the pooled effect of studies with low/moderate risk of bias.

6 Treatment effect in the nine studies with high risk of bias was *g* = −0.18 (95% CI = −0.37 to 0.02, *z* = −1.75, *p* = 0.081), which did not differ statistically significantly (*Q* = 0.48, *p* = 0.487) from the pooled effect of studies with low/moderate risk of bias.

7 Treatment effect in the two studies with high risk of bias was *g* = −0.63 (95% CI = −1.01 to −0.24, *z* = −3.20, *p* = 0.001), which did not differ statistically significantly (*Q* = 1.04, *p* = 0.309) from the pooled effect of studies with low/moderate risk of bias.

8 Naming this specific producer and their educational materials does not constitute an endorsement of this company or its products from the authors. The sole aim of naming a specific company's training materials is to help the reader get a better sense of what could be offered to improve healthcare professionals' knowledge base.

9 Naming this specific product does not constitute an endorsement from the authors. The sole aim of naming a specific product is to help the reader get a better sense of a relatively unusual and innovative approach to the question under study.

10 Intention‐to‐treat data were reported in most included studies. When such data were not available, this fact was considered and reported in the risk‐of‐bias assessments.

11 An excellent comparison of broad‐range micronutrient supplementation content and dosages across studies can be found in the supporting material of the work of Gast et al. (2023).
