# Supplementary material for: The Efficacy of Nutritional Interventions in Reducing Childhood/Youth Aggressive and Antisocial Behavior: A Mixed‐Methods Systematic Review and Meta‐Analysis
Source: Campbell Syst Rev. 2025 Aug 10;21(3):e70059. doi: 10.1002/cl2.70059 (PMC12335748; doi:10.1002/cl2.70059)
Supplement: Supplementary file 2 — Supporting Information S2: Final search strings of the electronic database searches. [file CL2-21-e70059-s001.docx]

| **[Ovid MEDLINE(R) ALL <1946 to February 22, 2024>](https://myaccess.library.utoronto.ca/login?url=http://ovidsp.ovid.com/ovidweb.cgi?T=JS&NEWS=N&PAGE=main&SHAREDSEARCHID=6xk6jLyG9Qw7sEPSq2o42x62A8WoosjuUzZbp2tGUrpZ4wHnjpSSs5My5J6ASRnCM)**  1 (aggress* adj5 (behav* or conduct* or disorder* or issue* or problem* or demeanor or act*)).tw,kf.  2 (aggressiveness or aggression*).tw,kf.  3 (hetero adj3 aggress*).tw,kf.  4 heteroaggress*.kf,tw.  5 (extraggress* or hyperaggress* or microaggress*).kf,tw.  6 externaliz*.kf,tw.  7 ((oppositional* adj4 (defy or defian*)) or ((defy or defian*) adj4 disorder*) or (oppositional* adj4 disorder*)).kf,tw.  8 (conduct adj3 (disorder* or issue* or problem*)).kf,tw.  9 (anti-social* or antisocial* or sadis* or sociopath* or psychopath*).kf,tw.  10 (deliquen* or disobey* or disobedien* or shoplift* or shop-lift* or vandal* or arson* or rob or robber* or burglar* or assault* or murder* or homicide* or stalk* or incivility or kidnap* or manslaughter* or harass* or recidiv* or reoffend* or offen*).kf,tw.  11 violen*.kf,tw.  12 (rape or raped or rapes or raping or rapist* or molest*).kf,tw.  13 (sex* adj3 (nonconsen* or non-consen* or violat* or dominan* or coerc* or abus*)).kf,tw.  14 (fight* or fought or combative*).kf,tw.  15 (((explos* or explod*) adj4 disorder*) or (intermittent* adj4 (explod* or explos*))).kf,tw.  16 (bully* or bulli* or cyberbull*).kf,tw.  17 aggression/  18 problem behavior/ or cyberbullying/ or bullying/  19 Conduct Disorder/ or "Disruptive, Impulse Control, and Conduct Disorders"/ or Antisocial Personality Disorder/  20 homicide/ or recidivism/ or sex offenses/ or rape/ or theft/ or violence/ or domestic violence/ or gun violence/ or intimate partner violence/ or physical abuse/ or workplace violence/ or Harassment, Non-Sexual/ or Sexual Harassment/ or firesetting behavior/ or emotional abuse/ or incivility/ or juvenile delinquency/ or stalking/ or criminal behavior/  21 1 or 2 or 3 or 4 or 5 or 6 or 7 or 8 or 9 or 10 or 11 or 12 or 13 or 14 or 15 or 16 or 17 or 18 or 19 or 20  22 (vitamin* or multivitamin* or provitamin* or previtamin*).kf,tw.  23 (nutri* or macronutri* or micronutri*).kf,tw.  24 (nutraceutic* or nutrapharm* or nutra-pharm*).kf,tw.  25 mineral*.kf,tw.  26 phytoceutic*.kf,tw.  27 (food or foods or feed or feeds or feeding or fed).kf,tw.  28 (diet or diets or dietary).kf,tw.  29 ((supplement or supplementing or supplemented or supplements) adj4 (eat* or ate or consum* or ingest* or drink* or drank or beverage* or administer* or give* or giving or gave or provid* or take* or taking or took or intak*)).kf,tw.  30 (vegetable* or fruit or fruits or meat or meats or pork or beef or chicken or poultry or venison or veal or fish or shellfish or seafood* or (corn adj3 syrup*) or dairy or milk or cheese* or wheat or nondairy or juice or juices or smoothie or smoothies or soda or sodas or coffee* or tea or teas or egg or eggs or water or dessert* or candy or candies or chocolate* or bread or breads or cereal* or tofu or sugary or prebiotic or prebiotics or pre-biotic or pre-biotics or probiotic or probiotics or pro-biotic or pro-biotics or sweeten* or unsweeten*).kf,tw.  31 dh.fs.  32 (plant-base* or plantbase* or vegetarian* or vegan* or lactoovoveg* or ovoveg* or lactoveg* or keto or ketogenic* or pescetarian* or paleo* or flexitarian* or carnivor* or raw or gluten free or mediterranean or (intermit* adj3 fast*) or macrobiotic or omnivor* or fodmap or pescoveg*).kf,tw.  33 (pufa or pufas or mufa or mufas or (omega adj3 ("3" or "6" or "9" or "12" or oil* or fat or acid*))).kf,tw.  34 ((herb* or eastern or Chinese or plant* or traditional or ancient or alternative or complementary or natural) adj4 (medic* or remed* or heal* or drug or drugs) adj4 (eat* or ate or consum* or ingest* or drink* or drank or beverage* or supplement or supplements or pill or pills or tablet* or capsule* or intak*)).kf,tw.  35 ((herb* or plant or plants) adj4 (product* or preparation* or therap* or treat* or extract*) adj4 (eat* or ate or consum* or ingest* or drink* or drank or beverage* or supplement or supplements or pill or pills or tablet* or capsule* or intak*)).kf,tw.  36 ((ethnopharm* or phytotherap* or phytomedicin* or ethnobotan* or ethnomedic* or botanical*) adj4 (eat* or ate or consum* or ingest* or drink* or drank or beverage* or supplement or supplements or pill or pills or tablet* or capsule* or intak*)).kf,tw.  37 ((5htp or (st adj3 john* adj3 wort*) or 5-htp or acid or acids or additive* or amino or antioxidant* or anti-oxidant* or arachidonic* or ascorb* or aspartame or biotin or boric or boron or caffein* or calciferol or calcitriol or calcium or camphor or carb or carbohydrate* or carbs or carnitin* or carotene or casein or cassava or cesium or chamomile or chlorid* or chlorin* or cholecalciferol or choline or chromium or cobalamin or cobalt or cobamid* or coenzyme q10 or colecalciferol or copper or creatin* or dehydroascorb* or docosahexaeno* or docosapentaeno* or eicosadieno* or eicosapentaeno* or eicosatetraeno* or eicosatrieno* or ergocalciferol* or erythritol or fat or fats or fatty or fiber or fibre or flax* or fluorid* or flurorin* or folacin or folate or folic or fructose or garlic or ginger or ginkgo or ginseng or glucosamine or glucose or glutamine or grain or grains or histidine or hydroxocobalamin* or hydroxymethylbutyrate or hydroxytryptophan or ingredient* or inositol or iodine or iron or isoleucine or jimson weed or khat or kratom or lactose or legume* or leucine or licorice or linoleic* or linolen* or lipid or lipids or liquorice or lysine or magnesium or manganese or melatonin or methionine or niacin or nicotinamide or nicotinic or nicotinuric or nickel or nut or nuts or oat or oats or oil or phenylalanine or phosphate* or potassium or protein or proteins or pyridoxine or retinaldehyde* or retinoic or retinoid or retinol or retinyl* or riboflavin or saccharin or salt* or selenium or sodium or soy or soya or soybean* or starch or sucrose or sugar* or sulfur or taurine or thiamin* or threonine or tin or tocopherol* or tocotrienol* or transfat* or triglycerid* or tri-glycerid* or tryptophan or ubiquinon* or valine or valpro* or whey or xylitol or yerba mate or yoghurt* or yogurt* or zinc) adj4 (eat* or ate or consum* or ingest* or drink* or drank or beverage* or supplement or supplements or pill or pills or tablet* or capsule* or intak*)).kf,tw.  38 ((sport* or electrolyte or soft or carbonat* or fizz* or energy) adj4 (drink or drinks or beverage* or water*)).kf,tw.  39 ((high or low or mid or moderate or less or more) adj4 (carb* or protein or proteins or fat or fats or fatty or calori*)).kf,tw.  40 ((processed or organic or junk or nonorganic or fermented or nonperishable or gmo or (genetically adj2 modified) or fortif*) adj4 (eat* or ate or consum* or ingest* or drink* or drank or beverage* or intak*)).kf,tw.  41 (trace elements/ or 24,25-dihydroxyvitamin d 3/ or 25-hydroxyvitamin d 2/ or calcifediol/ or cholecalciferol/ or cod liver oil/ or beta carotene/ or acetylcarnitine/ or biotin/ or folic acid/ or inositol/ or niacin/ or pyridoxal/ or pyridoxamine/ or pyridoxine/ or riboflavin/ or thiamine/ or synbiotics/ or food preservatives/ or plant preparations/ or plant extracts/ or flower essences/ or curare/ or drugs, chinese herbal/ or lecithins/ or plant oils/ or eucalyptus oil/ or rapeseed oil/ or castor oil/ or clove oil/ or linseed oil/ or palm oil/ or rice bran oil/ or sunflower oil/ or "tea tree oil"/ or Phytotherapy/ or Phytochemicals/ or lipids/ or fatty acids/ or eicosanoic acids/ or fatty acids, unsaturated/ or eicosanoids/ or arachidonic acids/ or arachidonic acid/ or complementary therapies/ or hydroxyeicosatetraenoic acids/ or 12-hydroxy-5,8,10,14-eicosatetraenoic acid/ or eicosapentaenoic acid/ or 5,8,11,14-eicosatetraynoic acid/ or 8,11,14-eicosatrienoic acid/ or fatty acids, essential/ or linoleic acids/ or linoleic acid/ or linolenic acids/ or alpha-linolenic acid/ or gamma-linolenic acid/ or fatty acids, monounsaturated/ or capsaicin/ or erucic acids/ or oleic acids/ or oleic acid/ or undecylenic acids/ or fatty acids, omega-3/ or docosahexaenoic acids/ or fatty acids, omega-6/ or linoleic acids, conjugated/ or sorbic acid/ or trans fatty acids/ or heptanoic acids/ or palmitic acids/ or palmitates/ or palmitic acid/ or stearic acids/ or stearates/ or triglycerides/ or "amino acids, peptides, and proteins"/ or proteins/ or antioxidants/ or ascorbic acid/ or ergothioneine/ or grape seed extract/ or lycopene/ or melatonin/ or quercetin/ or resveratrol/ or silymarin/ or thioctic acid/ or amino acids/ or histidine/ or isoleucine/ or leucine/ or lysine/ or methionine/ or phenylalanine/ or threonine/ or tryptophan/ or valine/ or zeta carotene/) and (eat* or ate or consum* or ingest* or drink* or drank or beverage* or intak*).kf,tw.  42 micronutrients/ or vitamins/ or vitamin a/ or vitamin d/ or vitamin e/ or vitamin k/ or vitamin k 1/ or vitamin k 2/ or vitamin k 3/ or vitamin u/ or provitamins/ or vitamin b complex/ or vitamin b 12/ or vitamin b 6/ or "diet, food, and nutrition"/ or beverages/ or artificially sweetened beverages/ or carbonated beverages/ or carbonated water/ or drinking water/ or energy drinks/ or fermented beverages/ or buttermilk/ or kefir/ or kombucha tea/ or koumiss/ or "fruit and vegetable juices"/ or milk/ or cultured milk products/ or whey/ or milk substitutes/ or soy milk/ or sugar-sweetened beverages/ or tea/ or teas, herbal/ or teas, medicinal/ or fermented foods/ or soy foods/ or food/ or candy/ or chocolate/ or spices/ or edible grain/ or whole grains/ or dairy products/ or whey proteins/ or dietary carbohydrates/ or dietary sugars/ or dietary sucrose/ or high fructose corn syrup/ or dietary fats/ or dietary fats, unsaturated/ or corn oil/ or cottonseed oil/ or olive oil/ or safflower oil/ or sesame oil/ or soybean oil/ or dietary fiber/ or prebiotics/ or dietary proteins/ or animal proteins, dietary/ or egg proteins, dietary/ or meat proteins/ or fish proteins, dietary/ or poultry proteins/ or shellfish proteins/ or milk proteins/ or plant proteins, dietary/ or fruit proteins/ or grain proteins/ or nut proteins/ or pea proteins/ or soybean proteins/ or dietary supplements/ or probiotics/ or yeast, dried/ or eggs/ or egg white/ or egg yolk/ or fast foods/ or flour/ or food ingredients/ or food additives/ or fat substitutes/ or flavoring agents/ or sodium chloride, dietary/ or sweetening agents/ or stevia/ or sucrose/ or sugars/ or xylitol/ or non-nutritive sweeteners/ or nutritive sweeteners/ or food, fortified/ or food, genetically modified/ or food, organic/ or food, preserved/ or frozen foods/ or food, processed/ or foods, specialized/ or food, formulated/ or infant food/ or fruit/ or functional food/ or honey/ or meat/ or meat products/ or poultry/ or poultry products/ or red meat/ or pork meat/ or seafood/ or fish products/ or fish flour/ or shellfish/ or nuts/ or raw foods/ or salads/ or seeds/ or vegetables/ or vegetable products/ or coffee/ or nutrition therapy/ or diet therapy/ or diet, carbohydrate loading/ or diet, carbohydrate-restricted/ or diet, high-protein low-carbohydrate/ or diet, ketogenic/ or diet, fat-restricted/ or diet, gluten-free/ or diet, high-protein/ or diet, mediterranean/ or diet, paleolithic/ or diet, protein-restricted/ or diet, reducing/ or diet, sodium-restricted/ or diet, vegetarian/ or diet, macrobiotic/ or diet, vegan/ or minerals/  43 22 or 23 or 24 or 25 or 26 or 27 or 28 or 29 or 30 or 31 or 32 or 33 or 34 or 35 or 36 or 37 or 38 or 39 or 40 or 41 or 42  44 young adult/ or infant/ or infant, newborn/ or infant, large for gestational age/ or infant, low birth weight/ or infant, small for gestational age/ or infant, very low birth weight/ or infant, extremely low birth weight/ or infant, postmature/ or infant, premature/ or infant, extremely premature/ or adolescent/ or child/ or child, abandoned/ or child, adopted/ or child, exceptional/ or child, gifted/ or "child of impaired parents"/ or child, foster/ or child, orphaned/ or child, unwanted/ or disabled children/ or child, preschool/ or homeless youth/ or minors/ or adolescent fathers/ or adolescent mothers/ or adolescent, hospitalized/ or adolescent, institutionalized/ or child, hospitalized/ or child, institutionalized/ or Only Child/ or Students/ or Schools/ or Universities/  45 (infan* or baby or babies or toddler* or preschool* or kindergar* or child* or girl* or boy* or kid or kids or pediatric* or paediatric* or prepubesc* or preteen* or junior high or juvenile* or youth* or pubescen* or teen* or adolescen* or under-age* or underage* or school* or highschool* or student* or (young adj3 (men or man or woman or women or male* or female* or person* or people* or population* or individual* or adult*)) or emerging adult* or early adult*).kf,tw.  46 ((college* or universit*) adj5 (women or woman or man or men or people* or person* or attend*)).kf,tw.  47 (undergrad* or graduate* or post-secondar* or postsecondar*).kf,tw.  48 44 or 45 or 46 or 47  49 21 and 43 and 48  [**Embase Classic+Embase <1947 to 2024 February 22>**](https://myaccess.library.utoronto.ca/login?url=http://ovidsp.ovid.com/ovidweb.cgi?T=JS&NEWS=N&PAGE=main&SHAREDSEARCHID=2bjWIiQK1PkSKjDrkVePTccIG6xuCRC1lxt7NbQGd6EusXFo0jl1pDEdAxmm28xji)  1 (aggress* adj5 (behav* or conduct* or disorder* or issue* or problem* or demeanor or act*)).kf,tw.  2 (aggressiveness or aggression*).kf,tw.  3 (hetero adj3 aggress*).kf,tw.  4 heteroaggress*.kf,tw.  5 (extraggress* or hyperaggress* or microaggress*).kf,tw.  6 externaliz*.kf,tw.  7 ((oppositional* adj4 (defy or defian*)) or ((defy or defian*) adj4 disorder*) or (oppositional* adj4 disorder*)).kf,tw.  8 (conduct adj3 (disorder* or issue* or problem*)).kf,tw.  9 (anti-social* or antisocial* or sadis* or sociopath* or psychopath*).kf,tw.  10 (deliquen* or disobey* or disobedien* or shoplift* or shop-lift* or vandal* or arson* or rob or robber* or burglar* or assault* or murder* or homicide* or stalk* or incivility or kidnap* or manslaughter* or harass* or recidiv* or reoffend* or offen*).kf,tw.  11 violen*.kf,tw.  12 (rape or raped or rapes or raping or rapist* or molest*).kf,tw.  13 (sex* adj3 (nonconsen* or non-consen* or violat* or dominan* or coerc* or abus*)).kf,tw.  14 (fight* or fought or combative*).kf,tw.  15 (((explos* or explod*) adj4 disorder*) or (intermittent* adj4 (explod* or explos*))).kf,tw.  16 (bully* or bulli* or cyberbull*).kf,tw.  17 acid attack (violence)/ or "externalization (behavior)"/ or abuse/ or acquaintance rape/ or aggression/ or aggressiveness/ or animal abuse/ or antisocial behavior/ or antisocial personality disorder/ or arson/ or assault/ or attempted rape/ or battered woman/ or battering/ or bullying/ or burglary/ or conduct disorder/ or corpse dismemberment/ or criminal behavior/ or cyberbullying/ or cybergrooming/ or cyberstalking/ or dating violence/ or delinquency/ or domestic violence/ or emotional abuse/ or emotional neglect/ or ethnic conflict/ or externalizing disorder/ or family violence/ or fighting/ or frotteurism/ or gun violence/ or harassment/ or homicide/ or incivility/ or infanticide/ or intermittent explosive disorder/ or juvenile delinquency/ or kidnapping/ or marital rape/ or microaggression/ or non-sexual harassment/ or online harassment/ or oppositional defiant disorder/ or partner violence/ or physical abuse/ or physical violence/ or problem behavior/ or property crime/ or psychopathy/ or pyromania/ or rape/ or recidivism/ or robbery/ or sadism/ or sadistic personality disorder/ or sadomasochism/ or sexual abuse/ or sexual bullying/ or sexual coercion/ or sexual crime/ or sexual exploitation/ or sexual harassment/ or sexual misconduct/ or sexual sadism disorder/ or sexual sadism/ or shoplifting/ or stalking/ or theft/ or vandalism/ or verbal hostility/ or violence/ or workplace violence/  18 1 or 2 or 3 or 4 or 5 or 6 or 7 or 8 or 9 or 10 or 11 or 12 or 13 or 14 or 15 or 16 or 17  19 (vitamin* or multivitamin* or provitamin* or previtamin*).kf,tw.  20 (nutri* or macronutri* or micronutri*).kf,tw.  21 (nutraceutic* or nutrapharm* or nutra-pharm*).kf,tw.  22 mineral*.kf,tw.  23 phytoceutic*.kf,tw.  24 (food or foods or feed or feeds or feeding or fed).kf,tw.  25 (diet or diets or dietary).kf,tw.  26 ((supplement or supplementing or supplemented or supplements) adj4 (eat* or ate or consum* or ingest* or drink* or drank or beverage* or administer* or give* or giving or gave or provid* or take* or taking or took or intak*)).kf,tw.  27 (vegetable* or fruit or fruits or meat or meats or pork or beef or chicken or poultry or venison or veal or fish or shellfish or seafood* or (corn adj3 syrup*) or dairy or milk or cheese* or wheat or nondairy or juice or juices or smoothie or smoothies or soda or sodas or coffee* or tea or teas or egg or eggs or water or dessert* or candy or candies or chocolate* or bread or breads or cereal* or tofu or sugary or prebiotic or prebiotics or pre-biotic or pre-biotics or probiotic or probiotics or pro-biotic or pro-biotics or sweeten* or unsweeten*).kf,tw.  28 (plant-base* or plantbase* or vegetarian* or vegan* or lactoovoveg* or ovoveg* or lactoveg* or keto or ketogenic* or pescetarian* or paleo* or flexitarian* or carnivor* or raw or gluten free or mediterranean or (intermit* adj3 fast*) or macrobiotic or omnivor* or fodmap or pescoveg*).kf,tw.  29 (pufa or pufas or mufa or mufas or (omega adj3 ("3" or "6" or "9" or "12" or oil* or fat or acid*))).kf,tw.  30 ((herb* or eastern or Chinese or plant* or traditional or ancient or alternative or complementary or natural) adj4 (medic* or remed* or heal* or drug or drugs) adj4 (eat* or ate or consum* or ingest* or drink* or drank or beverage* or supplement or supplements or pill or pills or tablet* or capsule* or intak*)).kf,tw.  31 ((herb* or plant or plants) adj4 (product* or preparation* or therap* or treat* or extract*) adj4 (eat* or ate or consum* or ingest* or drink* or drank or beverage* or supplement or supplements or pill or pills or tablet* or capsule* or intak*)).kf,tw.  32 ((ethnopharm* or phytotherap* or phytomedicin* or ethnobotan* or ethnomedic* or botanical*) adj4 (eat* or ate or consum* or ingest* or drink* or drank or beverage* or supplement or supplements or pill or pills or tablet* or capsule* or intak*)).kf,tw.  33 ((5htp or (st adj3 john* adj3 wort*) or 5-htp or acid or acids or additive* or amino or antioxidant* or anti-oxidant* or arachidonic* or ascorb* or aspartame or biotin or boric or boron or caffein* or calciferol or calcitriol or calcium or camphor or carb or carbohydrate* or carbs or carnitin* or carotene or casein or cassava or cesium or chamomile or chlorid* or chlorin* or cholecalciferol or choline or chromium or cobalamin or cobalt or cobamid* or coenzyme q10 or colecalciferol or copper or creatin* or dehydroascorb* or docosahexaeno* or docosapentaeno* or eicosadieno* or eicosapentaeno* or eicosatetraeno* or eicosatrieno* or ergocalciferol* or erythritol or fat or fats or fatty or fiber or fibre or flax* or fluorid* or flurorin* or folacin or folate or folic or fructose or garlic or ginger or ginkgo or ginseng or glucosamine or glucose or glutamine or grain or grains or histidine or hydroxocobalamin* or hydroxymethylbutyrate or hydroxytryptophan or ingredient* or inositol or iodine or iron or isoleucine or jimson weed or khat or kratom or lactose or legume* or leucine or licorice or linoleic* or linolen* or lipid or lipids or liquorice or lysine or magnesium or manganese or melatonin or methionine or niacin or nicotinamide or nicotinic or nicotinuric or nickel or nut or nuts or oat or oats or oil or phenylalanine or phosphate* or potassium or protein or proteins or pyridoxine or retinaldehyde* or retinoic or retinoid or retinol or retinyl* or riboflavin or saccharin or salt* or selenium or sodium or soy or soya or soybean* or starch or sucrose or sugar* or sulfur or taurine or thiamin* or threonine or tin or tocopherol* or tocotrienol* or transfat* or triglycerid* or tri-glycerid* or tryptophan or ubiquinon* or valine or valpro* or whey or xylitol or yerba mate or yoghurt* or yogurt* or zinc) adj4 (eat* or ate or consum* or ingest* or drink* or drank or beverage* or supplement or supplements or pill or pills or tablet* or capsule* or intak*)).kf,tw.  34 ((sport* or electrolyte or soft or carbonat* or fizz* or energy) adj4 (drink or drinks or beverage* or water*)).kf,tw.  35 ((high or low or mid or moderate or less or more) adj4 (carb* or protein or proteins or fat or fats or fatty or calori*)).kf,tw.  36 ((processed or organic or junk or nonorganic or fermented or nonperishable or gmo or (genetically adj2 modified) or fortif*) adj4 (eat* or ate or consum* or ingest* or drink* or drank or beverage* or intak*)).kf,tw.  37 high fat/high fructose diet/ or "high fat/high glucose diet"/ or "high fat/high sucrose diet"/ or "licorice (confectionary)"/ or "ready to use supplementary food"/ or "ready to use therapeutic food"/ or acidified milk/ or acidophilus milk/ or almond milk/ or aloe juice/ or alternate day fasting/ or amino acid intake/ or apple juice/ or artificial milk/ or artificially sweetened beverage/ or atherogenic diet/ or baby food/ or baked milk/ or beef/ or beetroot juice/ or bergamot juice/ or berry juice/ or bitter orange juice/ or blackcurrant juice/ or blueberry juice/ or boiled milk/ or brazil nut/ or buffalo milk/ or cactus pear juice/ or caffeine intake/ or calcium intake/ or caloric intake/ or camel milk/ or candy/ or canned food/ or carbohydrate diet/ or carbohydrate intake/ or carbonated beverage/ or carbonated water/ or cardamom/ or cariogenic diet/ or carrot juice/ or cashew apple juice/ or cheese/ or cherry juice/ or chicken meat/ or chocolate milk/ or chocolate/ or chokeberry juice/ or cholesterol diet/ or cholesterol intake/ or citrus juice/ or clam juice/ or coconut milk/ or coffee consumption/ or coffee/ or condensed milk/ or convenience food/ or cooked food/ or copper intake/ or cow milk/ or crab meat/ or cranberry juice/ or dairy cream/ or dairy product/ or diet/ or dietary fiber/ or dietary intake/ or dietary pattern/ or dietary reference intake/ or dietary supplement/ or donkey milk/ or dried food/ or drinking water/ or dry matter intake/ or eating habit/ or eating/ or egg powder/ or egg white/ or egg yolk/ or egg/ or electrolyte intake/ or elemental diet/ or energy drink/ or evaporated milk/ or fast food/ or fat content/ or fat intake/ or fat load/ or feeding behavior/ or fermented beverage/ or fermented milk drink/ or fiber intake/ or fish consumption/ or fish meal/ or fish meat/ or fish product/ or fish roe/ or fish sauce/ or flavored milk/ or fluid intake/ or fodmap diet/ or folate intake/ or food access/ or food grain/ or food intake/ or food/ or fortified food/ or frozen food/ or fructose intake/ or fruit consumption/ or fruit juice/ or fruit/ or fruitarian diet/ or functional food/ or genetically modified food/ or glucose intake/ or gluten free casein free diet/ or gluten free diet/ or gluten/ or goat milk/ or grape juice/ or grapefruit juice/ or health food/ or healthy diet/ or healthy food access/ or herbal tea/ or high calorie diet/ or high fiber diet/ or high fodmap diet/ or high potassium intake/ or high sodium intake/ or high-fructose diet/ or high-glucose diet/ or high-protein low-carbohydrate diet/ or high-sucrose diet/ or horse milk/ or infant feeding/ or inorganic nutrient/ or insect-based food/ or instant coffee/ or instant food/ or intermittent fasting/ or iodine intake/ or iron intake/ or iron restriction/ or junk food/ or ketogenic diet/ or lactoovovegetarian diet/ or lactose free diet/ or lactovegetarian diet/ or lamb meat/ or lemon juice/ or lime juice/ or lipid diet/ or low calorie diet/ or low carbohydrate diet/ or low fiber diet/ or low fodmap diet/ or low iodine diet/ or low residue diet/ or macrobiotic diet/ or macronutrient intake/ or macronutrient/ or magnesium intake/ or mandarin juice/ or mango juice/ or mate tea/ or meal size/ or meal skipping/ or meal/ or meat consumption/ or meat juice/ or meat protein/ or meat substitute/ or meat/ or medicinal tea/ or mediterranean diet/ or micronutrient intake/ or milk by origin/ or milk by preservation/ or milk powder/ or milk protein/ or milk substitute/ or milkshake/ or mind diet/ or mineral intake/ or mineral water/ or minimally processed food/ or multivitamin/ or nordic diet/ or nut/ or nutrient intake/ or nutrient solution/ or nutrient/ or nutrition/ or nutritional value/ or okinawan diet/ or orange juice/ or organic food/ or organic nutrient/ or ovovegetarian diet/ or paleolithic diet/ or pasteurized milk/ or peanut milk/ or pear juice/ or personalized nutrition/ or pescovegetarian diet/ or phosphate intake/ or phytonutrient/ or pickled food/ or pig milk/ or pine nut/ or pineapple juice/ or pistachio nut/ or plant nutrient/ or plant nutrition/ or plant-based milk/ or pomegranate juice/ or pork/ or portion size/ or potassium intake/ or potassium restriction/ or poultry egg/ or poultry meat/ or poultry product/ or preserved food/ or processed cereal/ or processed culinary ingredient/ or processed fish/ or processed food/ or processed fruit/ or processed meat/ or processed vegetable/ or protein diet/ or protein intake/ or rabbit meat/ or raw food diet/ or raw food/ or raw meat/ or raw milk/ or red meat/ or rice milk/ or salt intake/ or sea food/ or selenium intake/ or sheep milk/ or shellfish/ or smoked fish/ or smoked food/ or smoked meat/ or sodium appetite/ or sodium intake/ or sodium restriction/ or soft drink/ or soy food/ or soy sauce/ or soybean milk/ or sport nutrition/ or sports drink/ or staple food/ or strawberry juice/ or sugar confectionary/ or sugar intake/ or sugar-sweetened beverage/ or superfood/ or sweetened beverage/ or sweetened condensed milk/ or sweetened milk/ or tea consumption/ or tea/ or textured vegetable protein/ or time restricted feeding/ or tofu/ or tomato juice/ or turkey meat/ or uht milk/ or ultra-processed food/ or unhealthy diet/ or veal/ or vegan diet/ or vegetable consumption/ or vegetable juice/ or vegetable/ or vegetarian diet/ or venison/ or very low calorie diet/ or very low calorie ketogenic diet/ or vitamin b complex/ or vitamin b group/ or vitamin d/ or vitamin intake/ or vitamin k group/ or vitamin/ or water deprivation/ or water kefir/ or watermelon juice/ or western diet/ or whey protein/ or whey/ or white meat/ or whole food/ or wild meat/ or yak milk/ or yoghurt/ or zinc intake/  38 ("ascorbic acid 2 [3,4 dihydro 2,5,7,8 tetramethyl 2 (4,8,12 trimethyltridecyl) 2h 1 benzopyran 6 yl hydrogen phosphate] potassium"/ or "carbohydrates and carbohydrate derivatives"/ or 4 aminobenzoate potassium plus ascorbic acid plus hydrocortisone plus potassium salicylate/ or 5 hydroxytryptophan/ or alpha tocopherol derivative/ or alpha tocopherol nicotinate/ or alpha tocopherol plus ascorbic acid/ or alpha tocopherol plus ergocalciferol plus ergocalciferol plus retinol palmitate/ or alpha tocopherol succinate/ or alpha tocopherol/ or alpha tocotrienol/ or amino acid mixture plus carbohydrates plus lipids plus minerals plus vitamins/ or amino acid/ or antioxidant/ or arachidonic acid/ or ascorbate magnesium/ or ascorbic acid 2 octadecyl ether/ or ascorbic acid 2 octyl ether/ or ascorbic acid 2 phosphate/ or ascorbic acid 2 sulfate/ or ascorbic acid derivative/ or ascorbic acid plus bioflavonoid plus menadione/ or ascorbic acid plus bioflavonoid/ or ascorbic acid plus carbonyl iron plus cyanocobalamin plus folic acid/ or ascorbic acid plus cyanocobalamin plus ferrous fumarate plus folic acid plus intrinsic factor/ or ascorbic acid plus cyanocobalamin plus ferrous fumarate/ or ascorbic acid plus rutoside/ or ascorbic acid plus vitamin b complex/ or ascorbic acid/ or ascorbyl gamolenate/ or ascorbyl palmitate/ or ascorbyl stearate/ or beta tocopherol/ or biotin derivative/ or biotin/ or boron/ or calcitriol 26,23 lactone/ or calcitriol derivative/ or calcitriol/ or calcium ascorbate/ or calcium carbonate plus colecalciferol plus risedronic acid/ or calcium carbonate plus ferrous fumarate plus vitamin d/ or calcium phosphate dibasic plus cyanocobalamin plus ethinylestradiol plus methylphenidate plus methyltestosterone plus nicotinamide plus pyridoxine plus riboflavin plus thiamine/ or cesium/ or choline bitartrate plus cyanocobalamin plus folic acid plus inositol plus methionine/ or chromium derivative/ or chromium/ or cis fatty acid/ or citrate calcium plus mineral plus multivitamin/ or cobalamin derivative/ or cobalamin/ or cobalt derivative/ or cobalt/ or coconut oil/ or cod liver oil/ or conjugated linoleic acid/ or corn oil/ or corticotropin plus cyanocobalamin/ or cotton seed oil/ or cyanocobalamin plus cyanocobalamin co 57 plus cyanocobalamin co 58/ or cyanocobalamin plus cyanocobalamin co 57 plus intrinsic factor/ or cyanocobalamin plus dexpanthenol plus nikethamide plus pyridoxine plus riboflavin plus thiamine/ or cyanocobalamin plus folic acid plus iron polysaccharide/ or cyanocobalamin plus folic acid plus nicotinamide plus pyridoxine plus riboflavin plus pantothenic acid plus thiamine/ or cyanocobalamin plus folic acid/ or cyanocobalamin plus nicotinamide plus pyridoxine plus riboflavin plus thiamine/ or cyanocobalamin plus rescinnamine plus thiamine/ or cyanocobalamin plus tannin plus zinc acetate/ or cyanocobalamin/ or delta tocopherol/ or docosahexaenoic acid/ or doxylamine plus pyridoxine/ or edible oil/ or ergocalciferol derivative/ or ergocalciferol/ or essential amino acid/ or essential fatty acid/ or extra virgin olive oil/ or fat substitute/ or fat/ or fatty acid derivative/ or fatty acid/ or ferrous fumarate plus folic acid plus multivitamin/ or ferrous sulfate plus fluoride plus multivitamin/ or ferrous sulfate plus multivitamin/ or fish oil/ or fluoride plus mineral plus vitamin/ or fluoride plus vitamin/ or folic acid derivative/ or folic acid plus nicotinamide plus zinc oxide/ or folic acid/ or food dye/ or food preservative/ or gamma linolenic acid/ or gamma tocopherol/ or gamma tocotrienol/ or glutamine plus minerals plus vitamins/ or glutamine plus tributyrin plus minerals plus vitamins/ or herb/ or histidine/ or iodinated poppyseed oil/ or isoleucine/ or leucine/ or linoleic acid ethyl ester/ or linoleic acid/ or linolenic acid/ or lipid/ or long chain fatty acid/ or lysine/ or manganese derivative/ or manganese/ or meclozine plus pyridoxine/ or medium chain fatty acid/ or methionine/ or minerals plus multivitamins/ or nickel/ or nicotinamide derivative/ or nicotinamide/ or olive oil/ or omega 3 acid ethyl ester/ or omega 3 fatty acid ester/ or omega 3 fatty acid/ or omega 6 fatty acid/ or palm kernel oil/ or palm oil/ or pantothenate calcium plus pyridoxine plus riboflavin plus thiamine/ or pantothenic acid 4' phosphate/ or phenylalanine/ or phytochemical/ or plant medicinal product/ or polyunsaturated fatty acid/ or potassium derivative/ or potassium/ or prebiotic agent/ or primrose oil/ or probiotic agent/ or protein concentrate plus carbohydrates plus lipids plus minerals plus vitamins/ or protein concentrate plus carbohydrates plus minerals plus vitamins/ or protein concentrate plus minerals plus vitamins/ or protein/ or provitamin/ or pyridoxine derivative/ or pyridoxine plus thiamine/ or pyridoxine/ or rapeseed oil/ or red palm oil/ or resistant starch/ or retinal/ or retinoic acid derivative/ or retinoic acid/ or retinoid derivative/ or retinoid/ or retinol acetate/ or retinol derivative/ or retinol ester/ or retinol methyl ether/ or retinol palmitate/ or retinol stearate/ or retinol/ or riboflavin derivative/ or riboflavin/ or rice bran oil/ or safflower oil plus soybean oil/ or safflower oil/ or saturated fatty acid/ or sesame seed oil/ or short chain fatty acid/ or sodium derivative/ or sodium/ or soybean meal/ or soybean oil/ or starch/ or sugar alcohol/ or sugar/ or sunflower oil/ or sweetening agent/ or thiamine derivative/ or thiamine disulfide/ or thiamine phosphate/ or thiamine triphosphate/ or thiamine/ or threonine/ or tin derivative/ or tin/ or tocopherol derivative/ or tocopherol/ or trans fatty acid/ or tryptophan/ or ubiquinone/ or unsaturated fatty acid/ or valine/ or valproic acid/ or very long chain fatty acid/ or virgin olive oil/ or vitamin d derivative/ or vitamin mixture/ or volatile fatty acid/) and (eat* or ate or consum* or ingest* or drink* or drank or beverage* or intak*).kf,tw.  39 19 or 20 or 21 or 22 or 23 or 24 or 25 or 26 or 27 or 28 or 29 or 30 or 31 or 32 or 33 or 34 or 35 or 36 or 37 or 38  40 (infan* or baby or babies or toddler* or preschool* or kindergar* or child* or girl* or boy* or kid or kids or pediatric* or paediatric* or prepubesc* or preteen* or junior high or juvenile* or youth* or pubescen* or teen* or adolescen* or under-age* or underage* or school* or highschool* or student* or (young adj3 (men or man or woman or women or male* or female* or person* or people* or population* or individual* or adult*)) or emerging adult* or early adult*).kf,tw.  41 ((college* or universit*) adj5 (women or woman or man or men or people* or person* or attend*)).kf,tw.  42 (undergrad* or graduate* or post-secondar* or postsecondar*).kf,tw.  43 child of impaired parents/ or abandoned child/ or adolescence/ or adolescent behavior/ or adolescent father/ or adolescent health/ or adolescent mother/ or adolescent parent/ or adolescent/ or adopted child/ or baby/ or boy/ or brain damaged child/ or child health/ or child psychology/ or child/ or childhood/ or elementary student/ or foster child/ or gifted child/ or girl/ or handicapped child/ or high school student/ or high school/ or hospitalized adolescent/ or hospitalized child/ or institutionalized adolescent/ or institutionalized child/ or juvenile/ or kindergarten/ or middle school student/ or middle school/ or nursery school/ or orphaned child/ or preschool child/ or primary school/ or school child/ or school/ or single parent child/ or student/ or toddler/ or unwanted child/ or young adult/  44 40 or 41 or 42 or 43  45 18 and 39 and 44  46 child nutrition/ or adolescent nutrition/  47 18 and 46  48 45 or 47    [**APA PsycInfo <1806 to February Week 3 2024>**](https://myaccess.library.utoronto.ca/login?url=http://ovidsp.ovid.com/ovidweb.cgi?T=JS&NEWS=N&PAGE=main&SHAREDSEARCHID=1UTbz1pVBSYe1YLTWzYhALmmUwkY2sFC76dLAQqGnKOiDSRDqTs5EJsF4SkdWKFdj)  1 (aggress* adj5 (behav* or conduct* or disorder* or issue* or problem* or demeanor or act*)).tw.  2 (aggressiveness or aggression*).tw.  3 (hetero adj3 aggress*).tw.  4 heteroaggress*.tw.  5 (extraggress* or hyperaggress* or microaggress*).tw.  6 externaliz*.tw.  7 ((oppositional* adj4 (defy or defian*)) or ((defy or defian*) adj4 disorder*) or (oppositional* adj4 disorder*)).tw.  8 (conduct adj3 (disorder* or issue* or problem*)).tw.  9 (anti-social* or antisocial* or sadis* or sociopath* or psychopath*).tw.  10 (deliquen* or disobey* or disobedien* or shoplift* or shop-lift* or vandal* or arson* or rob or robber* or burglar* or assault* or murder* or homicide* or stalk* or incivility or kidnap* or manslaughter* or harass* or recidiv* or reoffend* or offen*).tw.  11 violen*.tw.  12 (rape or raped or rapes or raping or rapist* or molest*).tw.  13 (sex* adj3 (nonconsen* or non-consen* or violat* or dominan* or coerc* or abus*)).tw.  14 (fight* or fought or combative*).tw.  15 (((explos* or explod*) adj4 disorder*) or (intermittent* adj4 (explod* or explos*))).tw.  16 (bully* or bulli* or cyberbull*).tw.  17 acquaintance rape/ or aggressive behavior/ or aggressiveness/ or antisocial behavior/ or antisocial personality disorder/ or arson/ or attack behavior/ or behavior problems/ or bullying/ or coercion/ or conduct disorder/ or conflict/ or corruption/ or crime/ or criminal behavior/ or criminal offenders/ or cruelty/ or cyberbullying/ or dangerousness/ or dating violence/ or dishonesty/ or domestic violence/ or elder abuse/ or emotional abuse/ or explosive disorder/ or externalization/ or externalizing symptoms/ or family conflict/ or female criminal offenders/ or female delinquency/ or gender violence/ or gun violence/ or harassment/ or hate crimes/ or homicide/ or impulse control disorders/ or incest/ or infanticide/ or intimate partner violence/ or juvenile delinquency/ or kidnapping/ or kleptomania/ or male criminal offenders/ or male delinquency/ or mass murder/ or microaggression/ or oppositional defiant disorder/ or physical abuse/ or predelinquent youth/ or psychopathology/ or psychopathy/ or psychoticism/ or pyromania/ or rape/ or recidivism/ or riots/ or sadism/ or school violence/ or serial homicide/ or sex offenses/ or sex trafficking/ or sexual abuse/ or sexual coercion/ or sexual harassment/ or sexual sadism/ or sexual violence/ or shoplifting/ or stalking/ or teasing/ or theft/ or threat/ or vandalism/ or verbal abuse/ or violence/ or violent crime/ or virtual violence/ or workplace violence/  18 1 or 2 or 3 or 4 or 5 or 6 or 7 or 8 or 9 or 10 or 11 or 12 or 13 or 14 or 15 or 16 or 17  19 (vitamin* or multivitamin* or provitamin* or previtamin*).tw.  20 (nutri* or macronutri* or micronutri*).tw.  21 (nutraceutic* or nutrapharm* or nutra-pharm*).tw.  22 mineral*.tw.  23 phytoceutic*.tw.  24 (food or foods or feed or feeds or feeding or fed).tw.  25 (diet or diets or dietary).tw.  26 ((supplement or supplementing or supplemented or supplements) adj4 (eat* or ate or consum* or ingest* or drink* or drank or beverage* or administer* or give* or giving or gave or provid* or take* or taking or took or intak*)).tw.  27 (vegetable* or fruit or fruits or meat or meats or pork or beef or chicken or poultry or venison or veal or fish or shellfish or seafood* or (corn adj3 syrup*) or dairy or milk or cheese* or wheat or nondairy or juice or juices or smoothie or smoothies or soda or sodas or coffee* or tea or teas or egg or eggs or water or dessert* or candy or candies or chocolate* or bread or breads or cereal* or tofu or sugary or prebiotic or prebiotics or pre-biotic or pre-biotics or probiotic or probiotics or pro-biotic or pro-biotics or sweeten* or unsweeten*).tw.  28 (plant-base* or plantbase* or vegetarian* or vegan* or lactoovoveg* or ovoveg* or lactoveg* or keto or ketogenic* or pescetarian* or paleo* or flexitarian* or carnivor* or raw or gluten free or mediterranean or (intermit* adj3 fast*) or macrobiotic or omnivor* or fodmap or pescoveg*).tw.  29 (pufa or pufas or mufa or mufas or (omega adj3 ("3" or "6" or "9" or "12" or oil* or fat or acid*))).tw.  30 ((herb* or eastern or Chinese or plant* or traditional or ancient or alternative or complementary or natural) adj4 (medic* or remed* or heal* or drug or drugs) adj4 (eat* or ate or consum* or ingest* or drink* or drank or beverage* or supplement or supplements or pill or pills or tablet* or capsule* or intak*)).tw.  31 ((herb* or plant or plants) adj4 (product* or preparation* or therap* or treat* or extract*) adj4 (eat* or ate or consum* or ingest* or drink* or drank or beverage* or supplement or supplements or pill or pills or tablet* or capsule* or intak*)).tw.  32 ((ethnopharm* or phytotherap* or phytomedicin* or ethnobotan* or ethnomedic* or botanical*) adj4 (eat* or ate or consum* or ingest* or drink* or drank or beverage* or supplement or supplements or pill or pills or tablet* or capsule* or intak*)).tw.  33 ((5htp or (st adj3 john* adj3 wort*) or 5-htp or acid or acids or additive* or amino or antioxidant* or anti-oxidant* or arachidonic* or ascorb* or aspartame or biotin or boric or boron or caffein* or calciferol or calcitriol or calcium or camphor or carb or carbohydrate* or carbs or carnitin* or carotene or casein or cassava or cesium or chamomile or chlorid* or chlorin* or cholecalciferol or choline or chromium or cobalamin or cobalt or cobamid* or coenzyme q10 or colecalciferol or copper or creatin* or dehydroascorb* or docosahexaeno* or docosapentaeno* or eicosadieno* or eicosapentaeno* or eicosatetraeno* or eicosatrieno* or ergocalciferol* or erythritol or fat or fats or fatty or fiber or fibre or flax* or fluorid* or flurorin* or folacin or folate or folic or fructose or garlic or ginger or ginkgo or ginseng or glucosamine or glucose or glutamine or grain or grains or histidine or hydroxocobalamin* or hydroxymethylbutyrate or hydroxytryptophan or ingredient* or inositol or iodine or iron or isoleucine or jimson weed or khat or kratom or lactose or legume* or leucine or licorice or linoleic* or linolen* or lipid or lipids or liquorice or lysine or magnesium or manganese or melatonin or methionine or niacin or nicotinamide or nicotinic or nicotinuric or nickel or nut or nuts or oat or oats or oil or phenylalanine or phosphate* or potassium or protein or proteins or pyridoxine or retinaldehyde* or retinoic or retinoid or retinol or retinyl* or riboflavin or saccharin or salt* or selenium or sodium or soy or soya or soybean* or starch or sucrose or sugar* or sulfur or taurine or thiamin* or threonine or tin or tocopherol* or tocotrienol* or transfat* or triglycerid* or tri-glycerid* or tryptophan or ubiquinon* or valine or valpro* or whey or xylitol or yerba mate or yoghurt* or yogurt* or zinc) adj4 (eat* or ate or consum* or ingest* or drink* or drank or beverage* or supplement or supplements or pill or pills or tablet* or capsule* or intak*)).tw.  34 ((sport* or electrolyte or soft or carbonat* or fizz* or energy) adj4 (drink or drinks or beverage* or water*)).tw.  35 ((high or low or mid or moderate or less or more) adj4 (carb* or protein or proteins or fat or fats or fatty or calori*)).tw.  36 ((processed or organic or junk or nonorganic or fermented or nonperishable or gmo or (genetically adj2 modified) or fortif*) adj4 (eat* or ate or consum* or ingest* or drink* or drank or beverage* or intak*)).tw.  37 ("hydroxytryptophan (5-)"/ or "medicinal herbs and plants"/ or alternative medicine/ or amino acids/ or antioxidants/ or arachidonic acid/ or ascorbic acid/ or calcium ions/ or calcium/ or capsaicin/ or carbohydrates/ or chloride ions/ or choline/ or cobalt/ or copper/ or electrolytes/ or fatty acids/ or folic acid/ or food additives/ or glucose/ or histidine/ or hypericum perforatum/ or iron/ or lecithin/ or leucine/ or lipids/ or magnesium ions/ or magnesium/ or melatonin/ or metallic elements/ or methionine/ or nicotinamide/ or nicotinic acid/ or phenylalanine/ or potassium ions/ or potassium/ or proteins/ or sleep aids/ or sodium ions/ or sodium/ or sugars/ or tryptophan/ or zinc/) and (eat* or ate or consum* or ingest* or drink* or drank or beverage* or intak*).tw.  38 caffeine/ or dietary supplements/ or diets/ or eating behavior/ or energy drink/ or fast food/ or fluid intake/ or food intake/ or food/ or healthy eating/ or meat consumption/ or nutrition/ or vegan diet/ or vegetarian diet/ or vitamin therapy/ or vitamins/ or water intake/  39 19 or 20 or 21 or 22 or 23 or 24 or 25 or 26 or 27 or 28 or 29 or 30 or 31 or 32 or 33 or 34 or 35 or 36 or 37 or 38  40 (infan* or baby or babies or toddler* or preschool* or kindergar* or child* or girl* or boy* or kid or kids or pediatric* or paediatric* or prepubesc* or preteen* or junior high or juvenile* or youth* or pubescen* or teen* or adolescen* or under-age* or underage* or school* or highschool* or student* or (young adj3 (men or man or woman or women or male* or female* or person* or people* or population* or individual* or adult*)) or emerging adult* or early adult*).tw.  41 ((college* or universit*) adj5 (women or woman or man or men or people* or person* or attend*)).tw.  42 (undergrad* or graduate* or post-secondar* or postsecondar*).tw.  43 children of alcoholics/ or "illegitimacy (child)"/ or adolescent attitudes/ or adolescent behavior/ or adolescent characteristics/ or adolescent development/ or adolescent fathers/ or adolescent health/ or adolescent mothers/ or adolescent pregnancy/ or adolescent psychology/ or adopted children/ or boarding schools/ or charter schools/ or child attitudes/ or child behavior/ or child characteristics/ or child health/ or child psychology/ or childhood development/ or college athletes/ or college students/ or colleges/ or community college students/ or community colleges/ or early adolescence/ or early childhood development/ or education students/ or elementary school students/ or elementary schools/ or emerging adulthood/ or adolescent psychology/ or high school students/ or high schools/ or intermediate school students/ or junior college students/ or junior high school students/ or junior high schools/ or kindergarten students/ or kindergartens/ or late adolescence/ or middle school students/ or middle schools/ or military schools/ or nursery school students/ or nursery schools/ or nursing students/ or only children/ or orphans/ or preschool students/ or primary school students/ or puberty/ or rotc students/ or schools/ or special education students/ or student attitudes/ or student characteristics/ or students/ or technical schools/  44 40 or 41 or 42 or 43  45 18 and 39 and 44  46 adolescent psychopathology/ or child psychopathology/  47 39 and 46  48 45 or 47      [**AMED (Allied and Complementary Medicine) <1985 to October 2023>**](https://myaccess.library.utoronto.ca/login?url=http://ovidsp.ovid.com/ovidweb.cgi?T=JS&NEWS=N&PAGE=main&SHAREDSEARCHID=HM9obsOsIvX9NHwBeYTPcsGj4jSqp1n6jq1GTYzC6KitzoUBKNGVUZbJWui3eKLq) | |
| --- | --- |
| 1 | (aggress* adj5 (behav* or conduct* or disorder* or issue* or problem* or demeanor or act*)).tw. |
| 2 | (aggressiveness or aggression*).tw. |
| 3 | (hetero adj3 aggress*).tw. |
| 4 | heteroaggress*.tw. |
| 5 | (extraggress* or hyperaggress* or microaggress*).tw. |
| 6 | externaliz*.tw. |
| 7 | ((oppositional* adj4 (defy or defian*)) or ((defy or defian*) adj4 disorder*) or (oppositional* adj4 disorder*)).tw. |
| 8 | (conduct adj3 (disorder* or issue* or problem*)).tw. |
| 9 | (anti-social* or antisocial* or sadis* or sociopath* or psychopath*).tw. |
| 10 | (deliquen* or disobey* or disobedien* or shoplift* or shop-lift* or vandal* or arson* or rob or robber* or burglar* or assault* or murder* or homicide* or stalk* or incivility or kidnap* or manslaughter* or harass* or recidiv* or reoffend* or offen*).tw. |
| 11 | violen*.tw. |
| 12 | (rape or raped or rapes or raping or rapist* or molest*).tw. |
| 13 | (sex* adj3 (nonconsen* or non-consen* or violat* or dominan* or coerc* or abus*)).tw. |
| 14 | (fight* or fought or combative*).tw. |
| 15 | (((explos* or explod*) adj4 disorder*) or (intermittent* adj4 (explod* or explos*))).tw. |
| 16 | (bully* or bulli* or cyberbull*).tw. |
| 17 | aggression/ |
| 18 | violence/ or domestic violence/ or sexual abuse/ or sexual harassment/ or sex offenses/ or mental abuse/ or juvenile delinquency/ |
| 19 | 1 or 2 or 3 or 4 or 5 or 6 or 7 or 8 or 9 or 10 or 11 or 12 or 13 or 14 or 15 or 16 or 17 or 18 |
| 20 | (vitamin* or multivitamin* or provitamin* or previtamin*).tw. |
| 21 | (nutri* or macronutri* or micronutri*).tw. |
| 22 | (nutraceutic* or nutrapharm* or nutra-pharm*).tw. |
| 23 | mineral*.tw. |
| 24 | phytoceutic*.tw. |
| 25 | (food or foods or feed or feeds or feeding or fed).tw. |
| 26 | (diet or diets or dietary).tw. |
| 27 | ((supplement or supplementing or supplemented or supplements) adj4 (eat* or ate or consum* or ingest* or drink* or drank or beverage* or administer* or give* or giving or gave or provid* or take* or taking or took or intak*)).tw. |
| 28 | (vegetable* or fruit or fruits or meat or meats or pork or beef or chicken or poultry or venison or veal or fish or shellfish or seafood* or (corn adj3 syrup*) or dairy or milk or cheese* or wheat or nondairy or juice or juices or smoothie or smoothies or soda or sodas or coffee* or tea or teas or egg or eggs or water or dessert* or candy or candies or chocolate* or bread or breads or cereal* or tofu or sugary or prebiotic or prebiotics or pre-biotic or pre-biotics or probiotic or probiotics or pro-biotic or pro-biotics or sweeten* or unsweeten*).tw. |
| 29 | (plant-base* or plantbase* or vegetarian* or vegan* or lactoovoveg* or ovoveg* or lactoveg* or keto or ketogenic* or pescetarian* or paleo* or flexitarian* or carnivor* or raw or gluten free or mediterranean or (intermit* adj3 fast*) or macrobiotic or omnivor* or fodmap or pescoveg*).tw. |
| 30 | (pufa or pufas or mufa or mufas or (omega adj3 ("3" or "6" or "9" or "12" or oil* or fat or acid*))).tw. |
| 31 | ((herb* or eastern or Chinese or plant* or traditional or ancient or alternative or complementary or natural) adj4 (medic* or remed* or heal* or drug or drugs) adj4 (eat* or ate or consum* or ingest* or drink* or drank or beverage* or supplement or supplements or pill or pills or tablet* or capsule* or intak*)).tw. |
| 32 | ((herb* or plant or plants) adj4 (product* or preparation* or therap* or treat* or extract*) adj4 (eat* or ate or consum* or ingest* or drink* or drank or beverage* or supplement or supplements or pill or pills or tablet* or capsule* or intak*)).tw. |
| 33 | ((ethnopharm* or phytotherap* or phytomedicin* or ethnobotan* or ethnomedic* or botanical*) adj4 (eat* or ate or consum* or ingest* or drink* or drank or beverage* or supplement or supplements or pill or pills or tablet* or capsule* or intak*)).tw. |
| 34 | ((5htp or (st adj3 john* adj3 wort*) or 5-htp or acid or acids or additive* or amino or antioxidant* or anti-oxidant* or arachidonic* or ascorb* or aspartame or biotin or boric or boron or caffein* or calciferol or calcitriol or calcium or camphor or carb or carbohydrate* or carbs or carnitin* or carotene or casein or cassava or cesium or chamomile or chlorid* or chlorin* or cholecalciferol or choline or chromium or cobalamin or cobalt or cobamid* or coenzyme q10 or colecalciferol or copper or creatin* or dehydroascorb* or docosahexaeno* or docosapentaeno* or eicosadieno* or eicosapentaeno* or eicosatetraeno* or eicosatrieno* or ergocalciferol* or erythritol or fat or fats or fatty or fiber or fibre or flax* or fluorid* or flurorin* or folacin or folate or folic or fructose or garlic or ginger or ginkgo or ginseng or glucosamine or glucose or glutamine or grain or grains or histidine or hydroxocobalamin* or hydroxymethylbutyrate or hydroxytryptophan or ingredient* or inositol or iodine or iron or isoleucine or jimson weed or khat or kratom or lactose or legume* or leucine or licorice or linoleic* or linolen* or lipid or lipids or liquorice or lysine or magnesium or manganese or melatonin or methionine or niacin or nicotinamide or nicotinic or nicotinuric or nickel or nut or nuts or oat or oats or oil or phenylalanine or phosphate* or potassium or protein or proteins or pyridoxine or retinaldehyde* or retinoic or retinoid or retinol or retinyl* or riboflavin or saccharin or salt* or selenium or sodium or soy or soya or soybean* or starch or sucrose or sugar* or sulfur or taurine or thiamin* or threonine or tin or tocopherol* or tocotrienol* or transfat* or triglycerid* or tri-glycerid* or tryptophan or ubiquinon* or valine or valpro* or whey or xylitol or yerba mate or yoghurt* or yogurt* or zinc) adj4 (eat* or ate or consum* or ingest* or drink* or drank or beverage* or supplement or supplements or pill or pills or tablet* or capsule* or intak*)).tw. |
| 35 | ((sport* or electrolyte or soft or carbonat* or fizz* or energy) adj4 (drink or drinks or beverage* or water*)).tw. |
| 36 | ((high or low or mid or moderate or less or more) adj4 (carb* or protein or proteins or fat or fats or fatty or calori*)).tw. |
| 37 | ((processed or organic or junk or nonorganic or fermented or nonperishable or gmo or (genetically adj2 modified) or fortif*) adj4 (eat* or ate or consum* or ingest* or drink* or drank or beverage* or intak*)).tw. |
| 38 | nutrition/ or diet/ or caloric intake/ or energy intake/ or nutritional status/ or drinking behavior/ or feeding behavior/ or fasting/ or eating/ |
| 39 | food and beverages/ or beverages/ or coffee/ or milk/ or tea/ or food/ or exp cereals/ or condiments/ or dairy products/ or dietary fats/ or dietary fiber/ or dietary proteins/ or dietary supplements/ or food additives/ or probiotics/ or exp fruit/ or honey/ or meat/ or exp vegetables/ or diet fads/ or diet mediterranean/ or diet vegetarian/ or vegetarianism/ or exp vitamins/ |
| 40 | (exp "amino acids peptides and proteins"/ or exp carbohydrates/ or plant extracts/ or acetogenins/ or herbal drugs/ or exp drugs chinese herbal/ or kava/ or phytoestrogens/ or resins plant/ or exp lipids/ or exp inorganic chemicals/ or water/ or exp electrolytes/ or exp minerals/ or salts/ or exp metals/ or exp organic chemicals/ or exp plants edible/ or exp plants medicinal/ or herbalism/ or naturopathy/ or exp phytotherapy/ or exp traditional medicine/) and (eat* or ate or consum* or ingest* or drink* or drank or beverage* or intak*).tw. |
| 41 | 20 or 21 or 22 or 23 or 24 or 25 or 26 or 27 or 28 or 29 or 30 or 31 or 32 or 33 or 34 or 35 or 36 or 37 or 38 or 39 or 40 |
| 42 | (infan* or baby or babies or toddler* or preschool* or kindergar* or child* or girl* or boy* or kid or kids or pediatric* or paediatric* or prepubesc* or preteen* or junior high or juvenile* or youth* or pubescen* or teen* or adolescen* or under-age* or underage* or school* or highschool* or student* or (young adj3 (men or man or woman or women or male* or female* or person* or people* or population* or individual* or adult*)) or emerging adult* or early adult*).tw. |
| 43 | ((college* or universit*) adj5 (women or woman or man or men or people* or person* or attend*)).tw. |
| 44 | (undergrad* or graduate* or post-secondar* or postsecondar*).tw. |
| 45 | adolescent/ or adolescence/ or child/ or child gifted/ or child hospitalized/ or child preschool/ or disabled children/ or infant/ or pediatrics/ or students/ or schools/ or schools nursery/ or universities/ |
| 46 | 42 or 43 or 44 or 45 |
| 47 | 19 and 41 and 46 |
| 48 | Child nutrition/ |
| 49 | 19 and 48 |
| 50 | 47 or 49  [Cochrane](https://www-cochranelibrary-com.myaccess.library.utoronto.ca/advanced-search/search-manager?search=7398422)  (aggress* NEAR/4 (behav* or conduct* or disorder* or issue* or problem* or demeanor or act*)):ti,ab,kw  (aggressiveness or aggression*):ti,ab,kw  (hetero NEAR/2 aggress*):ti,ab,kw  heteroaggress*:ti,ab,kw  (extraggress* or hyperaggress* or microaggress*):ti,ab,kw  externaliz*:ti,ab,kw  ((oppositional* NEAR/3 (defy or defian*)) or ((defy or defian*) NEAR/3 disorder*) or (oppositional* NEAR/3 disorder*)):ti,ab,kw  (conduct NEAR/2 (disorder* or issue* or problem*)):ti,ab,kw  (anti-social* or antisocial* or sadis* or sociopath* or psychopath*):ti,ab,kw  (deliquen* or disobey* or disobedien* or shoplift* or shop-lift* or vandal* or arson* or rob or robber* or burglar* or assault* or murder* or homicide* or stalk* or incivility or kidnap* or manslaughter* or harass* or recidiv* or reoffend* or offen*):ti,ab,kw  violen*:ti,ab,kw  (rape or raped or rapes or raping or rapist* or molest*):ti,ab,kw  (sex* NEAR/2 (nonconsen* or non-consen* or violat* or dominan* or coerc* or abus*)):ti,ab,kw  (fight* or fought or combative*):ti,ab,kw  (((explos* or explod*) NEAR/3 disorder*) or (intermittent* NEAR/3 (explod* or explos*))):ti,ab,kw  (bully* or bulli* or cyberbull*):ti,ab,kw  [mh ^“aggression”]  [mh ^“problem behavior”] or [mh ^“cyberbullying”] or [mh ^“bullying”]  [mh ^“Conduct Disorder”] or [mh ^”Disruptive, Impulse Control, and Conduct Disorders”] or [mh ^“Antisocial Personality Disorder”]  [mh ^“homicide”] or [mh ^“recidivism”] or [mh ^“sex offenses”] or [mh ^“rape”] or [mh ^“theft”] or [mh ^“violence”] or [mh ^“domestic violence”] or [mh ^“gun violence”] or [mh ^“intimate partner violence”] or [mh ^“physical abuse”] or [mh ^“workplace violence”] or [mh ^“Harassment, Non-Sexual”] or [mh ^“Sexual Harassment”] or [mh ^“firesetting behavior”] or [mh ^“emotional abuse”] or [mh ^“incivility”] or [mh ^“juvenile delinquency”] or [mh ^“stalking”] or [mh ^“criminal behavior”]  #1 or #2 or #3 or #4 or #5 or #6 or #7 or #8 or #9 or #10 or #11 or #12 or #13 or #14 or #15 or #16 or #17 or #18 or #19 or #20  (vitamin* or multivitamin* or provitamin* or previtamin*):ti,ab,kw  (nutri* or macronutri* or micronutri*):ti,ab,kw  (nutraceutic* or nutrapharm* or nutra-pharm*):ti,ab,kw  mineral*:ti,ab,kw  phytoceutic*:ti,ab,kw  (food or foods or feed or feeds or feeding or fed):ti,ab,kw  (diet or diets or dietary):ti,ab,kw  ((supplement or supplementing or supplemented or supplements) NEAR/3 (eat* or ate or consum* or ingest* or drink* or drank or beverage* or administer* or give* or giving or gave or provid* or take* or taking or took or intak*)):ti,ab,kw  (vegetable* or fruit or fruits or meat or meats or pork or beef or chicken or poultry or venison or veal or fish or shellfish or seafood* or (corn NEAR/2 syrup*) or dairy or milk or cheese* or wheat or nondairy or juice or juices or smoothie or smoothies or soda or sodas or coffee* or tea or teas or egg or eggs or water or dessert* or candy or candies or chocolate* or bread or breads or cereal* or tofu or sugary or prebiotic or prebiotics or pre-biotic or pre-biotics or probiotic or probiotics or pro-biotic or pro-biotics or sweeten* or unsweeten*):ti,ab,kw  Any MeSH descriptor in all MeSH products and with qualifier(s): [diet therapy - DH]  (plant-base* or plantbase* or vegetarian* or vegan* or lactoovoveg* or ovoveg* or lactoveg* or keto or ketogenic* or pescetarian* or paleo* or flexitarian* or carnivor* or raw or gluten free or mediterranean or (intermit* NEAR/2 fast*) or macrobiotic or omnivor* or fodmap or pescoveg*):ti,ab,kw  (pufa or pufas or mufa or mufas or (omega NEAR/2 ("3" or "6" or "9" or "12" or oil* or fat or acid*))):ti,ab,kw  ((herb* or eastern or Chinese or plant* or traditional or ancient or alternative or complementary or natural) NEAR/3 (medic* or remed* or heal* or drug or drugs) NEAR/3 (eat* or ate or consum* or ingest* or drink* or drank or beverage* or supplement or supplements or pill or pills or tablet* or capsule* or intak*)):ti,ab,kw  ((herb* or plant or plants) NEAR/3 (product* or preparation* or therap* or treat* or extract*) NEAR/3 (eat* or ate or consum* or ingest* or drink* or drank or beverage* or supplement or supplements or pill or pills or tablet* or capsule* or intak*)):ti,ab,kw  ((ethnopharm* or phytotherap* or phytomedicin* or ethnobotan* or ethnomedic* or botanical*) NEAR/3 (eat* or ate or consum* or ingest* or drink* or drank or beverage* or supplement or supplements or pill or pills or tablet* or capsule* or intak*)):ti,ab,kw  (((st NEAR/2 john* NEAR/2 wort*) or acid or acids or additive* or amino or antioxidant* or anti-oxidant* or arachidonic* or ascorb* or aspartame or biotin or boric or boron or caffein* or calciferol or calcitriol or calcium or camphor or carb or carbohydrate* or carbs or carnitin* or carotene or casein or cassava or cesium or chamomile or chlorid* or chlorin* or cholecalciferol or choline or chromium or cobalamin or cobalt or cobamid* or colecalciferol or copper or creatin* or dehydroascorb* or docosahexaeno* or docosapentaeno* or eicosadieno* or eicosapentaeno* or eicosatetraeno* or eicosatrieno* or ergocalciferol* or erythritol or fat or fats or fatty or fiber or fibre or flax* or fluorid* or flurorin* or folacin or folate or folic or fructose or garlic or ginger or ginkgo or ginseng or glucosamine or glucose or glutamine or grain or grains or histidine or hydroxocobalamin* or hydroxymethylbutyrate or hydroxytryptophan or ingredient* or inositol or iodine or iron or isoleucine or jimson weed or khat or kratom or lactose or legume* or leucine or licorice or linoleic* or linolen* or lipid or lipids or liquorice or lysine or magnesium or manganese or melatonin or methionine or niacin or nicotinamide or nicotinic or nicotinuric or nickel or nut or nuts or oat or oats or oil or phenylalanine or phosphate* or potassium or protein or proteins or pyridoxine or retinaldehyde* or retinoic or retinoid or retinol or retinyl* or riboflavin or saccharin or salt* or selenium or sodium or soy or soya or soybean* or starch or sucrose or sugar* or sulfur or taurine or thiamin* or threonine or tin or tocopherol* or tocotrienol* or transfat* or triglycerid* or tri-glycerid* or tryptophan or ubiquinon* or valine or valpro* or whey or xylitol or yerba mate or yoghurt* or yogurt* or zinc) NEAR/3 (eat* or ate or consum* or ingest* or drink* or drank or beverage* or supplement or supplements or pill or pills or tablet* or capsule* or intak*)):ti,ab,kw  (("5htp" or "5-htp" or "coenzyme q10") NEAR/3 (eat* or ate or consum* or ingest* or drink* or drank or beverage* or supplement or supplements or pill or pills or tablet* or capsule* or intak*)):ti,ab,kw  ((sport* or electrolyte or soft or carbonat* or fizz* or energy) NEAR/3 (drink or drinks or beverage* or water*)):ti,ab,kw  ((high or low or mid or moderate or less or more) NEAR/3 (carb* or protein or proteins or fat or fats or fatty or calori*)):ti,ab,kw  ((processed or organic or junk or nonorganic or fermented or nonperishable or gmo or (genetically NEAR/1 modified) or fortif*) NEAR/3 (eat* or ate or consum* or ingest* or drink* or drank or beverage* or intak*)):ti,ab,kw  ([mh ^“trace elements”] or [mh ^“calcifediol”] or [mh ^“cholecalciferol”] or [mh ^“cod liver oil”] or [mh ^“beta carotene”] or [mh ^“acetylcarnitine”] or [mh ^“biotin”] or [mh ^“folic acid”] or [mh ^“inositol”] or [mh ^“niacin”] or [mh ^“pyridoxal”] or [mh ^“pyridoxamine”] or [mh ^“pyridoxine”] or [mh ^“riboflavin”] or [mh ^“thiamine”] or [mh ^“synbiotics”] or [mh ^“food preservatives”] or [mh ^“plant preparations”] or [mh ^“plant extracts”] or [mh ^“flower essences”] or [mh ^“curare”] or [mh ^“drugs, chinese herbal”] or [mh ^“lecithins”] or [mh ^“plant oils”] or [mh ^“eucalyptus oil”] or [mh ^“rapeseed oil”] or [mh ^“castor oil”] or [mh ^“clove oil”] or [mh ^“linseed oil”] or [mh ^“palm oil”] or [mh ^“rice bran oil”] or [mh ^“sunflower oil”] or [mh ^”tea tree oil”] or [mh ^“Phytotherapy”] or [mh ^“Phytochemicals”] or [mh ^“lipids”] or [mh ^“fatty acids”] or [mh ^“eicosanoic acids”] or [mh ^“fatty acids, unsaturated”] or [mh ^“eicosanoids”] or [mh ^“eicosapentaenoic acid”] or [mh ^“arachidonic acids”] or [mh ^“arachidonic acid”] or [mh ^“complementary therapies”] or [mh ^“hydroxyeicosatetraenoic acids”] or [mh ^“fatty acids, essential”] or [mh ^“linoleic acids”] or [mh ^“linoleic acid”] or [mh ^“linolenic acids”] or [mh ^“alpha-linolenic acid”] or [mh ^“gamma-linolenic acid”] or [mh ^“fatty acids, monounsaturated”] or [mh ^“capsaicin”] or [mh ^“erucic acids”] or [mh ^“oleic acids”] or [mh ^“oleic acid”] or [mh ^“undecylenic acids”] or [mh ^“docosahexaenoic acids”] or [mh ^“linoleic acids, conjugated”] or [mh ^“sorbic acid”] or [mh ^“trans fatty acids”] or [mh ^“heptanoic acids”] or [mh ^“palmitic acids”] or [mh ^“palmitates”] or [mh ^“palmitic acid”] or [mh ^“stearic acids”] or [mh ^“stearates”] or [mh ^“triglycerides”] or [mh ^”amino acids, peptides, and proteins”] or [mh ^“proteins”] or [mh ^“antioxidants”] or [mh ^“ascorbic acid”] or [mh ^“ergothioneine”] or [mh ^“grape seed extract”] or [mh ^“lycopene”] or [mh ^“melatonin”] or [mh ^“quercetin”] or [mh ^“resveratrol”] or [mh ^“silymarin”] or [mh ^“thioctic acid”] or [mh ^“amino acids”] or [mh ^“histidine”] or [mh ^“isoleucine”] or [mh ^“leucine”] or [mh ^“lysine”] or [mh ^“methionine”] or [mh ^“phenylalanine”] or [mh ^“threonine”] or [mh ^“tryptophan”] or [mh ^“valine”] or [mh ^“zeta carotene”]) and (eat* or ate or consum* or ingest* or drink* or drank or beverage* or intak*):ti,ab,kw  MeSH descriptor: [24,25-Dihydroxyvitamin D 3] this term only  MeSH descriptor: [25-Hydroxyvitamin D 2] this term only  MeSH descriptor: [12-Hydroxy-5,8,10,14-eicosatetraenoic Acid] this term only  MeSH descriptor: [5,8,11,14-Eicosatetraynoic Acid] this term only  MeSH descriptor: [8,11,14-Eicosatrienoic Acid] this term only  MeSH descriptor: [Fatty Acids, Omega-3] this term only  MeSH descriptor: [Fatty Acids, Omega-6] this term only  #43 or #44 or #45 or #46 or #47 or #48 or #49  (eat* or ate or consum* or ingest* or drink* or drank or beverage* or intak*):ti,ab,kw  #50 and #51  [mh ^“micronutrients”] or [mh ^“vitamins”] or [mh ^“vitamin a”] or [mh ^“vitamin d”] or [mh ^“vitamin e”] or [mh ^“vitamin k”] or [mh ^“vitamin u”] or [mh ^“provitamins”] or [mh ^“vitamin b complex”] or [mh ^”diet, food, and nutrition”] or [mh ^“beverages”] or [mh ^“artificially sweetened beverages”] or [mh ^“carbonated beverages”] or [mh ^“carbonated water”] or [mh ^“drinking water”] or [mh ^“energy drinks”] or [mh ^“fermented beverages”] or [mh ^“buttermilk”] or [mh ^“kefir”] or [mh ^“kombucha tea”] or [mh ^“koumiss”] or [mh ^”fruit and vegetable juices”] or [mh ^“milk”] or [mh ^“cultured milk products”] or [mh ^“whey”] or [mh ^“milk substitutes”] or [mh ^“soy milk”] or [mh ^“sugar-sweetened beverages”] or [mh ^“tea”] or [mh ^“teas, herbal”] or [mh ^“teas, medicinal”] or [mh ^“fermented foods”] or [mh ^“soy foods”] or [mh ^“food”] or [mh ^“candy”] or [mh ^“chocolate”] or [mh ^“spices”] or [mh ^“edible grain”] or [mh ^“whole grains”] or [mh ^“dairy products”] or [mh ^“whey proteins”] or [mh ^“dietary carbohydrates”] or [mh ^“dietary sugars”] or [mh ^“dietary sucrose”] or [mh ^“high fructose corn syrup”] or [mh ^“dietary fats”] or [mh ^“dietary fats, unsaturated”] or [mh ^“corn oil”] or [mh ^“cottonseed oil”] or [mh ^“olive oil”] or [mh ^“safflower oil”] or [mh ^“sesame oil”] or [mh ^“soybean oil”] or [mh ^“dietary fiber”] or [mh ^“prebiotics”] or [mh ^“dietary proteins”] or [mh ^“animal proteins, dietary”] or [mh ^“egg proteins, dietary”] or [mh ^“meat proteins”] or [mh ^“fish proteins, dietary”] or [mh ^“poultry proteins”] or [mh ^“shellfish proteins”] or [mh ^“milk proteins”] or [mh ^“plant proteins, dietary”] or [mh ^“fruit proteins”] or [mh ^“grain proteins”] or [mh ^“nut proteins”] or [mh ^“pea proteins”] or [mh ^“soybean proteins”] or [mh ^“dietary supplements”] or [mh ^“probiotics”] or [mh ^“yeast, dried”] or [mh ^“eggs”] or [mh ^“egg white”] or [mh ^“egg yolk”] or [mh ^“fast foods”] or [mh ^“flour”] or [mh ^“food ingredients”] or [mh ^“food additives”] or [mh ^“fat substitutes”] or [mh ^“flavoring agents”] or [mh ^“sodium chloride, dietary”] or [mh ^“sweetening agents”] or [mh ^“stevia”] or [mh ^“sucrose”] or [mh ^“sugars”] or [mh ^“xylitol”] or [mh ^“non-nutritive sweeteners”] or [mh ^“nutritive sweeteners”] or [mh ^“food, fortified”] or [mh ^“food, genetically modified”] or [mh ^“food, organic”] or [mh ^“food, preserved”] or [mh ^“frozen foods”] or [mh ^“food, processed”] or [mh ^“foods, specialized”] or [mh ^“food, formulated”] or [mh ^“infant food”] or [mh ^“fruit”] or [mh ^“functional food”] or [mh ^“honey”] or [mh ^“meat”] or [mh ^“meat products”] or [mh ^“poultry”] or [mh ^“poultry products”] or [mh ^“red meat”] or [mh ^“pork meat”] or [mh ^“seafood”] or [mh ^“fish products”] or [mh ^“fish flour”] or [mh ^“shellfish”] or [mh ^“nuts”] or [mh ^“raw foods”] or [mh ^”salads”] or [mh ^“seeds”] or [mh ^“vegetables”] or [mh ^“vegetable products”] or [mh ^“coffee”] or [mh ^“nutrition therapy”] or [mh ^“diet therapy”] or [mh ^“diet, carbohydrate loading”] or [mh ^“diet, carbohydrate-restricted”] or [mh ^“diet, high-protein low-carbohydrate”] or [mh ^“diet, ketogenic”] or [mh ^“diet, fat-restricted”] or [mh ^“diet, gluten-free”] or [mh ^“diet, high-protein”] or [mh ^“diet, mediterranean”] or [mh ^“diet, paleolithic”] or [mh ^“diet, protein-restricted”] or [mh ^“diet, reducing”] or [mh ^“diet, sodium-restricted”] or [mh ^“diet, vegetarian”] or [mh ^“diet, macrobiotic”] or [mh ^“diet, vegan”] or [mh ^“minerals”]  MeSH descriptor: [Vitamin K 1] this term only  MeSH descriptor: [Vitamin K 2] this term only  MeSH descriptor: [Vitamin K 3] this term only  MeSH descriptor: [Vitamin B 6] this term only  MeSH descriptor: [Vitamin B 12] this term only  #22 or #23 or #24 or #25 or #26 or #27 or #28 or #29 or #30 or #31 or #32 or #33 or #34 or #35 or #36 or #37 or #38 or #39 or #40 or #41 or #42 or #52 or #53 or #54 or #55 or #56 or #57 or #58  [mh ^“young adult”] or [mh ^“infant”] or [mh ^“infant, newborn”] or [mh ^“infant, large for gestational age”] or [mh ^“infant, low birth weight”] or [mh ^“infant, small for gestational age”] or [mh ^“infant, very low birth weight”] or [mh ^“infant, extremely low birth weight”] or [mh ^“infant, postmature”] or [mh ^“infant, premature”] or [mh ^“infant, extremely premature”] or [mh ^“adolescent”] or [mh ^“child”] or [mh ^“child, abandoned”] or [mh ^“child, adopted”] or [mh ^“child, exceptional”] or [mh ^“child, gifted”] or [mh ^”child of impaired parents”] or [mh ^“child, foster”] or [mh ^“child, orphaned”] or [mh ^“child, unwanted”] or [mh ^“disabled children”] or [mh ^“child, preschool”] or [mh ^“homeless youth”] or [mh ^“minors”] or [mh ^“adolescent fathers”] or [mh ^“adolescent mothers”] or [mh ^“adolescent, hospitalized”] or [mh ^“adolescent, institutionalized”] or [mh ^“child, hospitalized”] or [mh ^“child, institutionalized”] or [mh ^“Only Child”] or [mh ^“Students”] or [mh ^“Schools”] or [mh ^“Universities”]  (infan* or baby or babies or toddler* or preschool* or kindergar* or child* or girl* or boy* or kid or kids or pediatric* or paediatric* or prepubesc* or preteen* or junior high or juvenile* or youth* or pubescen* or teen* or adolescen* or under-age* or underage* or school* or highschool* or student* or (young NEAR/2 (men or man or woman or women or male* or female* or person* or people* or population* or individual* or adult*)) or emerging adult* or early adult*):ti,ab,kw  ((college* or universit*) NEAR/4 (women or woman or man or men or people* or person* or attend*)):ti,ab,kw  (undergrad* or graduate* or post-secondar* or postsecondar*):ti,ab,kw  #60 or #61 or #62 or #63  #21 and #59 and #64  **Scopus**  ( ( TITLE-ABS-KEY ( ( aggress* W/4 ( behav* OR conduct* OR disorder* OR issue* OR problem* OR demeanor OR act* ) ) ) ) OR ( TITLE-ABS-KEY ( ( aggressiveness OR aggression* ) ) ) OR ( TITLE-ABS-KEY ( ( hetero W/2 aggress* ) ) ) OR ( TITLE-ABS-KEY ( heteroaggress* ) ) OR ( TITLE-ABS-KEY ( ( extraggress* OR hyperaggress* OR microaggress* ) ) ) OR ( TITLE-ABS-KEY ( externaliz* ) ) OR ( TITLE-ABS-KEY ( ( ( oppositional* W/3 ( defy OR defian* ) ) OR ( ( defy OR defian* ) W/3 disorder* ) OR ( oppositional* W/3 disorder* ) ) ) ) OR ( TITLE-ABS-KEY ( ( conduct W/2 ( disorder* OR issue* OR problem* ) ) ) ) OR ( TITLE-ABS-KEY ( ( anti-social* OR antisocial* OR sadis* OR sociopath* OR psychopath* ) ) ) OR ( TITLE-ABS-KEY ( ( deliquen* OR disobey* OR disobedien* OR shoplift* OR shop-lift* OR vandal* OR arson* OR rob OR robber* OR burglar* OR assault* OR murder* OR homicide* OR stalk* OR incivility OR kidnap* OR manslaughter* OR harass* OR recidiv* OR reoffend* OR offen* ) ) ) OR ( TITLE-ABS-KEY ( violen* ) ) OR ( TITLE-ABS-KEY ( ( rape OR raped OR rapes OR raping OR rapist* OR molest* ) ) ) OR ( TITLE-ABS-KEY ( ( sex* W/2 ( nonconsen* OR non-consen* OR violat* OR dominan* OR coerc* OR abus* ) ) ) ) OR ( TITLE-ABS-KEY ( ( fight* OR fought OR combative* ) ) ) OR ( TITLE-ABS-KEY ( ( ( ( explos* OR explod* ) W/3 disorder* ) OR ( intermittent* W/3 ( explod* OR explos* ) ) ) ) ) OR ( TITLE-ABS-KEY ( ( bully* OR bulli* OR cyberbull* ) ) ) ) AND ( ( TITLE-ABS-KEY ( ( vitamin* OR multivitamin* OR provitamin* OR previtamin* ) ) ) OR ( TITLE-ABS-KEY ( ( nutri* OR macronutri* OR micronutri* ) ) ) OR ( TITLE-ABS-KEY ( ( nutraceutic* OR nutrapharm* OR nutra-pharm* ) ) ) OR ( TITLE-ABS-KEY ( mineral* ) ) OR ( TITLE-ABS-KEY ( phytoceutic* ) ) OR ( TITLE-ABS-KEY ( ( food OR foods OR feed OR feeds OR feeding OR fed ) ) ) OR ( TITLE-ABS-KEY ( ( diet OR diets OR dietary ) ) ) OR ( TITLE-ABS-KEY ( ( ( supplement OR supplementing OR supplemented OR supplements ) W/3 ( eat* OR ate OR consum* OR ingest* OR drink* OR drank OR beverage* OR administer* OR give* OR giving OR gave OR provid* OR take* OR taking OR took OR intak* ) ) ) ) OR ( TITLE-ABS-KEY ( ( vegetable* OR fruit OR fruits OR meat OR meats OR pork OR beef OR chicken OR poultry OR venison OR veal OR fish OR shellfish OR seafood* OR ( corn W/2 syrup* ) OR dairy OR milk OR cheese* OR wheat OR nondairy OR juice OR juices OR smoothie OR smoothies OR soda OR sodas OR coffee* OR tea OR teas OR egg OR eggs OR water OR dessert* OR candy OR candies OR chocolate* OR bread OR breads OR cereal* OR tofu OR sugary OR prebiotic OR prebiotics OR pre-biotic OR pre-biotics OR probiotic OR probiotics OR pro-biotic OR pro-biotics OR sweeten* OR unsweeten* ) ) ) OR ( TITLE-ABS-KEY ( ( plant-base* OR plantbase* OR vegetarian* OR vegan* OR lactoovoveg* OR ovoveg* OR lactoveg* OR keto OR ketogenic* OR pescetarian* OR paleo* OR flexitarian* OR carnivor* OR raw OR "gluten free" OR mediterranean OR ( intermit* W/2 fast* ) OR macrobiotic OR omnivor* OR fodmap OR pescoveg* ) ) ) OR ( TITLE-ABS-KEY ( ( pufa OR pufas OR mufa OR mufas OR ( omega W/2 ( "3" OR "6" OR "9" OR "12" OR oil* OR fat OR acid* ) ) ) ) ) OR ( TITLE-ABS-KEY ( ( ( herb* OR eastern OR chinese OR plant* OR traditional OR ancient OR alternative OR complementary OR natural ) W/3 ( medic* OR remed* OR heal* OR drug OR drugs ) W/3 ( eat* OR ate OR consum* OR ingest* OR drink* OR drank OR beverage* OR supplement OR supplements OR pill OR pills OR tablet* OR capsule* OR intak* ) ) ) ) OR ( TITLE-ABS-KEY ( ( ( herb* OR plant OR plants ) W/3 ( product* OR preparation* OR therap* OR treat* OR extract* ) W/3 ( eat* OR ate OR consum* OR ingest* OR drink* OR drank OR beverage* OR supplement OR supplements OR pill OR pills OR tablet* OR capsule* OR intak* ) ) ) ) OR ( TITLE-ABS-KEY ( ( ( ethnopharm* OR phytotherap* OR phytomedicin* OR ethnobotan* OR ethnomedic* OR botanical* ) W/3 ( eat* OR ate OR consum* OR ingest* OR drink* OR drank OR beverage* OR supplement OR supplements OR pill OR pills OR tablet* OR capsule* OR intak* ) ) ) ) OR ( TITLE-ABS-KEY ( ( ( "5htp" OR ( st W/2 john* W/2 wort* ) OR "5-htp" OR acid OR acids OR additive* OR amino OR antioxidant* OR anti-oxidant* OR arachidonic* OR ascorb* OR aspartame OR biotin OR boric OR boron OR caffein* OR calciferol OR calcitriol OR calcium OR camphor OR carb OR carbohydrate* OR carbs OR carnitin* OR carotene OR casein OR cassava OR cesium OR chamomile OR chlorid* OR chlorin* OR cholecalciferol OR choline OR chromium OR cobalamin OR cobalt OR cobamid* OR "coenzyme q10" OR colecalciferol OR copper OR creatin* OR dehydroascorb* OR docosahexaeno* OR docosapentaeno* OR eicosadieno* OR eicosapentaeno* OR eicosatetraeno* OR eicosatrieno* OR ergocalciferol* OR erythritol OR fat OR fats OR fatty OR fiber OR fibre OR flax* OR fluorid* OR flurorin* OR folacin OR folate OR folic OR fructose OR garlic OR ginger OR ginkgo OR ginseng OR glucosamine OR glucose OR glutamine OR grain OR grains OR histidine OR hydroxocobalamin* OR hydroxymethylbutyrate OR hydroxytryptophan OR ingredient* OR inositol OR iodine OR iron OR isoleucine OR "jimson weed" OR khat OR kratom OR lactose OR legume* OR leucine OR licorice OR linoleic* OR linolen* OR lipid OR lipids OR liquorice OR lysine OR magnesium OR manganese OR melatonin OR methionine OR niacin OR nicotinamide OR nicotinic OR nicotinuric OR nickel OR nut OR nuts OR oat OR oats OR oil OR phenylalanine OR phosphate* OR potassium OR protein OR proteins OR pyridoxine OR retinaldehyde* OR retinoic OR retinoid OR retinol OR retinyl* OR riboflavin OR saccharin OR salt* OR selenium OR sodium OR soy OR soya OR soybean* OR starch OR sucrose OR sugar* OR sulfur OR taurine OR thiamin* OR threonine OR tin OR tocopherol* OR tocotrienol* OR transfat* OR triglycerid* OR tri-glycerid* OR tryptophan OR ubiquinon* OR valine OR valpro* OR whey OR xylitol OR "yerba mate" OR yoghurt* OR yogurt* OR zinc ) W/3 ( eat* OR ate OR consum* OR ingest* OR drink* OR drank OR beverage* OR supplement OR supplements OR pill OR pills OR tablet* OR capsule* OR intak* ) ) ) ) OR ( TITLE-ABS-KEY ( ( ( sport* OR electrolyte OR soft OR carbonat* OR fizz* OR energy ) W/3 ( drink OR drinks OR beverage* OR water* ) ) ) ) OR ( TITLE-ABS-KEY ( ( ( high OR low OR mid OR moderate OR less OR more ) W/3 ( carb* OR protein OR proteins OR fat OR fats OR fatty OR calori* ) ) ) ) OR ( TITLE-ABS-KEY ( ( ( processed OR organic OR junk OR nonorganic OR fermented OR nonperishable OR gmo OR ( genetically W/1 modified ) OR fortif* ) W/3 ( eat* OR ate OR consum* OR ingest* OR drink* OR drank OR beverage* OR intak* ) ) ) ) ) AND ( ( TITLE-ABS-KEY ( ( infan* OR baby OR babies OR toddler* OR preschool* OR kindergar* OR child* OR girl* OR boy* OR kid OR kids OR pediatric* OR paediatric* OR prepubesc* OR preteen* OR "junior high" OR juvenile* OR youth* OR pubescen* OR teen* OR adolescen* OR under-age* OR underage* OR school* OR highschool* OR student* OR ( young W/2 ( men OR man OR woman OR women OR male* OR female* OR person* OR people* OR population* OR individual* OR adult* ) ) OR "emerging adult*" OR "early adult*" ) ) ) OR ( TITLE-ABS-KEY ( ( ( college* OR universit* ) W/4 ( women OR woman OR man OR men OR people* OR person* OR attend* ) ) ) ) OR ( TITLE-ABS-KEY ( ( undergrad* OR graduate* OR post-secondar* OR postsecondar* ) ) ) ) |
|  |  |
